# Development, implementation and evaluation of an evidence-based paediatric early warning system improvement programme: the PUMA mixed methods study

**DOI:** 10.1186/s12913-021-07314-2

**Published:** 2022-01-02

**Authors:** Davina Allen, Amy Lloyd, Dawn Edwards, Kerenza Hood, Chao Huang, Jacqueline Hughes, Nina Jacob, David Lacy, Yvonne Moriarty, Alison Oliver, Jennifer Preston, Gerri Sefton, Ian Sinha, Richard Skone, Heather Strange, Khadijeh Taiyari, Emma Thomas-Jones, Rob Trubey, Lyvonne Tume, Colin Powell, Damian Roland

**Affiliations:** 1 School of Healthcare Sciences, Cardiff University, Room 13.08, Eastgate House, Newport Road, Cardiff, CF24 0AB UK; 2 Centre for Trials Research, Cardiff University, Neuadd Meirionnydd, Heath Park, Cardiff, UK; 3 Children’s Services, Swansea Bay University Health Board, Swansea, UK; 4 Hull-York Medical School, University of Hull, Hull, UK; 5 Arrow Park Hospital, Wirral University Teaching NHS Foundation Trust, Wirral, UK; 6 Noah’s Ark Children’s Hospital for Wales, Cardiff and Vale University Health Board, Cardiff, UK; 7 Alder Hey Clinical Research Facility, Institute in the Park, Alder Hey Children’s NHS Foundation Trust, Eaton Rd, Liverpool, UK; 8 Alder Hey Children’s Hospital, Alder Hey Children’s NHS Foundation Trust, Eaton Rd, Liverpool, UK; 9 School of Health and Society, University of Salford, Manchester, UK; 10 Department of Emergency Medicine, Sidra Medicine, Doha, Qatar; 11 Division of Population Medicine, School of Medicine, Cardiff University, Cardiff, UK; 12 Paediatric Emergency Medicine, Leicester Academic (PEMLA) Group, Emergency Department, University of Leicester, Leicester, UK; 13 SAPPHIRE Group, Health Sciences, Leicester University, Leicester, UK

**Keywords:** Paediatric early warning systems, Healthcare improvement, Quality improvement

## Abstract

**Background:**

Paediatric mortality rates in the United Kingdom are amongst the highest in Europe. Clinically missed deterioration is a contributory factor. Evidence to support any single intervention to address this problem is limited, but a cumulative body of research highlights the need for a systems approach.

**Methods:**

An evidence-based, theoretically informed, paediatric early warning system improvement programme (PUMA Programme) was developed and implemented in two general hospitals (no onsite Paediatric Intensive Care Unit) and two tertiary hospitals (with onsite Paediatric Intensive Care Unit) in the United Kingdom. Designed to harness local expertise to implement contextually appropriate improvement initiatives, the PUMA Programme includes a propositional model of a paediatric early warning system, system assessment tools, guidance to support improvement initiatives and structured facilitation and support.

Each hospital was evaluated using interrupted time series and qualitative case studies. The primary quantitative outcome was a composite metric (adverse events), representing the number of children monthly that experienced one of the following: mortality, cardiac arrest, respiratory arrest, unplanned admission to Paediatric Intensive Care Unit, or unplanned admission to Higher Dependency Unit. System changes were assessed qualitatively through observations of clinical practice and interviews with staff and parents. A qualitative evaluation of implementation processes was undertaken.

**Results:**

All sites assessed their paediatric early warning systems and identified areas for improvement. All made contextually appropriate system changes, despite implementation challenges. There was a decline in the adverse event rate trend in three sites; in one site where system wide changes were organisationally supported, the decline was significant (ß = -0.09 (95% CI: − 0.15, − 0.05); *p* = < 0.001). Changes in trends coincided with implementation of site-specific changes.

**Conclusions:**

System level change to improve paediatric early warning systems can bring about positive impacts on clinical outcomes, but in paediatric practice, where the patient population is smaller and clinical outcomes event rates are low, alternative outcome measures are required to support research and quality improvement beyond large specialist centres, and methodological work on rare events is indicated. With investment in the development of alternative outcome measures and methodologies, programmes like PUMA could improve mortality and morbidity in paediatrics and other patient populations.

**Supplementary Information:**

The online version contains supplementary material available at 10.1186/s12913-021-07314-2.

## Background

Missed deterioration is a cause of sub-optimal care in hospital patients, and Track and Trigger Tools (TTT), also known as Early Warning Scores (EWS), are a popular response to this problem. Deterioration is often preceded by a period of physiological instability which, when recognised, provides an opportunity for earlier intervention, and improved outcome. TTTs consist of sequential recording and monitoring of physiological, clinical, and observational data. When a certain score or trigger is reached, this directs a clinical action including, but not limited to, altered frequency of observation, a senior clinical review or more appropriate treatment or management. In the adult population, TTTs are deployed in several countries (Australia, USA, Netherlands), and in the UK a national early warning score, developed by the Royal College of Physicians and endorsed by NHS England and NHS Improvement is widely used. The use of TTTs in paediatrics is more challenging, however, because of variation in accepted physiological parameters across the age range.

Paediatric mortality rates in the United Kingdom are amongst the highest in Europe [1]. The PUMA study was commissioned to develop, implement, and evaluate a Paediatric Track and Trigger Tool (PTTT) for national implementation. Three linked evidence reviews were undertaken to inform the intervention, these focused on i) tool validity, ii) effectiveness in reducing mortality and critical events [2], and the iii) impact of the wider clinical microsystem (i.e. work practices and relationships, culture; and socio-technical infrastructure) on TTT use [3]. The two reviews on validity and effectiveness [2] found that several PTTTs have been evaluated, although most are derived from a limited number of original parent tools. Although many PTTTs have been narrowly validated in single centres or specialist units, none have been validated across different settings and populations, and many have only been tested in theory and modelling, rather than through use in practice. There is moderate evidence that paediatric early warning system interventions may reduce unplanned transfers to a higher level of care, but corresponding reductions in hospital-wide or paediatric intensive care unit mortality have not been reported. No studies evaluated a whole systems approach to improving the detection and response to deterioration. The third review highlighted multiple failure points in paediatric early warning systems: lack of monitoring equipment, inadequate staffing, knowledge deficits, insufficient situational awareness, poor inter-professional communication, uncertain escalation policies, and cultures that deter escalation. Several interventions to address specific system weaknesses have been proposed and some evaluated, but there is limited evidence to recommend their use. Overall, the findings of the three reviews did not support an exclusive focus on PTTTs to address the problem of missed deterioration and indicated the need for approaches that focus on the wider clinical microsystem. As a result of the findings from the reviews, we revised the study aims from an exclusive focus on a PTTT, to the development of a system wide improvement programme: The PUMA (Paediatric early warning system Utilisation and Morbidity Avoidance) Programme [4].

## Methods

### Study design

The research was a prospective, mixed-methods, before-and-after study, with two work streams.

#### Work Stream 1

Development and implementation of an evidence-based paediatric early warning system improvement programme (the PUMA Programme), drawing on three systematic reviews of the literature [2, 3].

#### Work Stream 2

Prospective mixed methods evaluation of the PUMA Programme in four UK hospitals, with an embedded qualitative formative and summative process evaluation.

A patient and public involvement (PPI) group informed both work streams. An experienced PPI lead (Jenny Preston) co-ordinated parent involvement throughout the study to advise on the tool and implementation package development (Work Steam 1); information leaflets for research ethics purposes; the design of interview schedules and the data generation templates; and qualitative data analysis, particularly parent interviews and dissemination strategies (Work Stream 2).

The study protocol covering the development, implementation and evaluation has been published [4]. Ethics approval was granted on 13 April 2015 by the National Research Ethics Service Committee South West, registration number 15/SW/0084.

### Theoretical framework

The study was informed by translational mobilisation theory (TMT) [5] and normalisation process theory [6]. TMT is a sociological theory, which provides a framework for understanding and investigating the organisation of collaborative work practices in institutional contexts. It was used to systematically analyse the socio-material relationships in paediatric early warning systems and the conditioning effects of the local institutional contexts [5]. Normalisation process theory (NPT) is a theory of implementation, which focuses on the actions necessary to embed a new intervention into practice. It informed the development and evaluation of the implementation strategy.

### Work stream 1: development and IMplementation of the PUMA Programme

The PUMA Programme was developed from the findings of the three systematic reviews [2, 3] and founded on OUTCOME, a novel approach to improvement. Informed by TMT, NPT, and the Model for Improvement [7] (see Additional Material 1, for a summary of the theories that underpin OUTCOME). OUTCOME was developed as part of the study and is intended to overcome some of the weaknesses of orthodox approaches to health-care improvement, namely:

- Solutions are often identified before problems are properly understood [8–10].
- Interventions are implemented without an understanding of the local systems of work in which they must have their effects [6, 8].
- The desire for standardisation limits freedom to adapt to local context [11].
- When an intervention is imposed from outside the organisation, there is little ownership and limited opportunity to capitalise on local expertise [12].
- Service-led projects that do utilise local expertise often lack adequate evaluation and reportage, which precludes shared learning [13].
- The form of an intervention is often given more consideration than its function – with a tendency to give precedence to a tool that can be implemented over an adjustment to the system [5].
- Improvement efforts are often time-limited and not sustained over the longer-term [12].

OUTCOME comprises six principles and is designed to support local teams to bring about the changes necessary to achieve a desired outcome in context specific ways. The OUTCOME principles and their application in the development of the PUMA Programme are described below and summarised in Table 1.

**Table 1:** The OUTCOME Framework: principles, structures, theory, and application in the PUMA study

PRINCIPLES	STRUCTURES	THEORY	PUMA
Outcome-directed			
Improvement is directed towards achieving an agreed outcome or goal	Specification of the collective action to be targeted for improvement and its overarching goal.	TMT	The goal of the PUMA study was to improve collective action in relation to the afferent component of a paediatric early warning system, which detects deterioration and triggers timely and appropriate action, and excluded the efferent component, which consists of the people and resources providing a response
Functions-oriented			
Improvement is oriented towards the functions necessary to achieve the goal	Specification of the core components, mechanisms of action and their relationships necessary to achieve the overarching goal.	TMT	Collective action in detecting and acting in response to deterioration includes detection (monitoring, recording, interpreting), preparation (reviewing, planning) and action (escalation, evaluation).
System-focused			
Improvement is focused on the socio-material system required to enact the functions necessary to achieving the goal	Minimum standards required to achieve the goal across contexts are specified (e.g. socio-material resources - people, materials, knowledge, processes and mechanisms)	TMT	In PUMA the minimal standards for a system for detecting acting on deterioration was specified in propositional model structured around the 7 core functions
Context-specific			
Improvement is focused on the development of locally appropriate initiatives to achieve the goals	Tools developed to assess systems against the standard	TMT/NPT	Staff System Assessment Tool Family Feedback Tool
Locally–led			
Improvement capitalises on the expertise and knowledge of those delivering services	Five step process to support improvement: 1) Form an improvement team 2) Assess the system 3) Select and plan improvement initiatives 4) Implement and review initiatives 5) Sustain progress	NPT Model for Improvement.	Improvement guide Structured facilitation On-going support
Learning systems			
Improvement is sustained by the creation of a learning system to optimise outcomes through the application of system assessment tools, to keep systems under review, and structures for supporting local leadership.	System Assessment Tools to enable reflexive monitoring Framework to support improvement process drawn from the Model for Improvement.	TMT NPT	Improvement Guide provided guidance on repeating the system assessment every 12–24 months to reflexively monitor performance, select and plan initiatives and implement and review initiatives.

#### Principle 1: outcomes directed

The first principle of OUTCOME is that improvement is driven by an agreed outcome, rather than by predefined interventions. This reflects a growing concern that health-care improvement is often solution driven, rather than focused on improving practice. The emphasis on outcomes in the framework is informed by the concept of ‘projects, which is the primary unit of analysis in TMT and refers to the network of people and materials oriented to a shared goal. Thinking about improvement in terms of the associated project helps to define the boundaries of the initiative. The literature on the detection of deterioration identifies four integrated components which work together to provide a safety system for at-risk patients: (1) the afferent component which detects deterioration and triggers timely and appropriate action; (2) the efferent component which consists of the people and resources providing a response; (3) a process improvement component, which includes system auditing and monitoring; and (4) an administrative component focusing on organisational leadership and education required to implement and sustain the system [14]. In the PUMA study, the project of interest was the afferent component of a paediatric early warning system, which detects deterioration and triggers timely and appropriate action, and excluded the efferent component, which consists of the people and resources providing a response.

#### Principle 2: functions oriented

The second principle of OUTCOME is that improvement is oriented towards the functions necessary to achieve the goal. This requires specification of the primary mechanisms of action that are necessary in an overall process for the goal to be achieved. In the PUMA study, the core functions of an afferent early warning system were identified through the application of TMT to the systematic review and refined through discussions with clinicians to produce seven functions in total: monitor, record, interpret, review, prepare, escalate, and evaluate [3].

#### Principle 3: systems focused

The third principle of OUTCOME is that improvement is focused on the socio-material resources, processes and mechanisms needed to enact the essential functions for achieving the goal. This requires specification of the minimum system requirements and draws on the concept of the strategic action field in TMT. Strategic action fields provide the structures, organising logics, technologies and materials, and interpretative repertoires that condition projects of collective action [5].

In the PUMA study, the system standard was specified in a propositional model of minimal conceptual requirements organised around the seven functions of an afferent paediatric early warning system (PUMA Standard). The model drew together two kinds of evidence from the systematic review: evidence of the challenges that must be overcome in detecting and acting on deterioration and evidence on proposed and/or evaluated solutions to challenges. The propositional model was reviewed and refined by parents with experience of a child’s deterioration and by clinical experts on the PUMA study team.

#### Principle 4: context specific

The fourth principle of OUTCOME is that improvement is focused on the development of context- specific initiatives to achieve the goal. Proponents of change often favour top-down approaches to bring about improvements; yet the list of interventions and improvement efforts that flounder when spread or scaled up continues to grow, [11, 12, 15] in part because of failures to normalise and embed interventions into local contexts. Avoiding these pitfalls requires structures to support systematic and rigorous local improvement efforts in relation to a service standard. In addition to specification of the minimum system requirements to support an improvement project, OUTCOME also involves the development of associated assessment tools that can be deployed to improve understanding of the local system and identify areas for improvement.

In the PUMA study, in collaboration with expert clinicians and parents, two complementary assessment tools were developed from the PUMA Standard: a Staff System Assessment Tool (SSAT) and a Family Feedback Tool (FFT). The tools were designed to prompt wider discussion among the improvement team, to reach a shared understanding of the local afferent paediatric warning system and areas that might be targeted for improvement.

#### Principle 5: locally led

The fifth principle of OUTCOME is that improvement capitalises on the expertise and knowledge of those delivering services. This is intended to encourage local ownership of the improvement initiative. The PUMA Programme included the development of an improvement guide drawing on the Model for Improvement to support teams in driving their own improvement processes and designed to operationalise the core constructs of NPT and start-up and action planning workshops to support local leadership of the improvement process [7].

#### Principle 6: learning systems

The final principle of OUTCOME is the creation of a learning system around the improvement project, with participants attuned to system features with strong feedback loops [12]. Health-care systems are dynamic, and wider changes to the system may be consequential for an area of practice, resulting in ‘drift, [16] or the need for further adjustments to the system. OUTCOME deploys the use of assessment tools to keep systems under review, and structures for supporting local leadership. In the PUMA Programme, this was reflected in written guidance on how to ‘sustain progress’ which included system assessments every 12–24 months to reflexively monitor performance, select and review initiatives.

The PUMA Programme was implemented in two tertiary children’s hospitals (with on site Paediatric Intensive Care Unit (PICU)) and two general hospitals (with no on site PICU) in the UK between June 2016 and November 2017. Two sites had a PTTT in place for the duration of the study, two did not (Table 2).

**Table 2:** Summary of Study Sites

	Site 1	Site 2	Site 3	Site 4
**Type of hospital**	Tertiary	District General	Tertiary	District General
**Paediatric Track and Trigger Tool in use at baseline.**	Yes	Yes	No	No
**Paediatric Intensive Care Unit (PICU) on site**	Yes	No	Yes	No
**Number of paediatric in-patient wards**	12	1	8	2
**Number of beds (excluding PICU)**	337 in-patients, 15 HDU	32 in-patient, 2 HDU	61 in-patient, 4 HDU	38 in-patient, 7 HDU
**Case Study Ward for qualitative data collection**	Cardiac medical/surgical and neonatal	General paediatric (might want to specify medical/surgical)	Medical	Medical

### Work stream 2: evaluation of the PUMA Programme

The study deployed an interrupted time series (ITS) design, in conjunction with ethnographic case studies, which combined observations and qualitative interviews, to evaluate changes in practice and outcomes over time. Ethnographic methods were also deployed in a formative and summative evaluation of implementation processes.

### Quantitative evaluation

The quantitative evaluation tracked monthly aggregate outcomes across all in-patient wards at each site for a minimum of 40 months (May 2015 – October 2018). The purpose was to evaluate the effect of the intervention on trends in markers of in-patient deterioration over time. Sites were analysed as separate case studies.

### Outcome measures

We identified eight outcome measures commonly reported in the literature [2] for assessment of the effectiveness of paediatric early warning systems: mortality, cardiac arrest, respiratory arrest, unplanned admission to PICU, unplanned admission to High Dependency Unit (HDU), PICU reviews, other medical emergencies requiring immediate assistance and non-ICU patient bed days. Each outcome definition was agreed with sites (Additional Material 2), piloting work was conducted to ensure the feasibility of data collection, and then consistently applied across all sites.

The primary quantitative outcome was a composite outcome metric (‘adverse events’) representing the total number of children in a given month that experienced at least one of the following events: mortality, cardiac arrest, respiratory arrest, unplanned admission to PICU, or unplanned admission to HDU. Secondary outcomes including the five components of the composite primary outcome (mortality, cardiac arrest, respiratory arrest, unplanned admission to PICU, unplanned admission to High Dependency Unit (HDU)) and also PICU reviews, other medical emergencies requiring immediate assistance and non-ICU patient bed days were analysed separately. Monthly patient-bed days (see Additional Material 2 for definition) were collected to calculate the rate.

### Sample size calculation

A simulation-based approach [17] was used to calculate power, based on the original study aims to develop and evaluate a PTTT. Whilst the primary outcome was a composite measure, there was limited availability of data and therefore we took the conservative option of focussing on unplanned transfers to PICU for our estimations. Utilising historical data from two sites, the prevalence of unplanned transfers to PICU was 1%. Additionally, previous research indicated that implementation of paediatric calling criteria with a rapid response team could result in a risk ratio of 0.65 for total avoidable hospital mortality [18]. We assumed that the PUMA intervention might result in a similar risk ratio [19]. The estimated effect size, mean difference, and common standard deviation were 2.8, 2.0 and 0.7, respectively. We estimated that 24-months of observations (12 pre- and 12 post) would give 90% power for an effect size is of least 2 [17]. When the research aims were changed from the implementation of a PTTT to the implementation of the PUMA Programme, we retained the focus on collecting 12 months pre- and 12 months post-intervention but allowed 12 months for phase in of the intervention to give a total of 36 months. We were able to collect data for up to 6 more months retrospectively for the pre-intervention period. This gave 42 months of data and increased our sample size.

### Analysis

All outcomes were expressed as rates per 1000 patient bed-days. In two sites, we received only partial denominator data for certain months (e.g., patient bed days were only recorded for 25 out of 30 days). In these cases, a weighted average relative to the month size was used to impute missing bed numbers and calculate monthly bed-days.

An ITS approach [20] was used to analyse data over time. Aggregate monthly rates of mortality and morbidity outcomes were tracked for up to 18 months before, 12 months during, and 12 months after implementation. A segmented linear regression was fitted on data from each site using an autoregressive integrated moving average (ARIMA) [21] method to analyse the primary and secondary outcomes. The assumptions of linear regression were checked investigating residual plots. The Durbin-Watson statistic, autocorrelation and partial autocorrelation function were used to identify the order of autocorrelation and moving average.

The most common approach to ITS analysis is to compare trends across two separate time periods: a pre- and post-intervention phase. Typically, the intervention is discrete and time-bounded - such as implementation of a PTTT – and thus might be expected to have an immediate effect on the outcome. In this study we expected that implementation of the PUMA Programme would take longer, but that we might be able to observe gradual changes in measures of in-patient deterioration. Therefore, we decided a priori to investigate both the short-term effect of the PUMA Programme (two phases taking the start of implementation as the time of change) and the longer-term effect (three phases incorporating pre, during and post change). We also used impact models [22] that allowed immediate (level) and trend (slope) change after introducing or completing implementation. Any statistically significant change in either level or trend would imply that the intervention had demonstrated an effect on outcomes.

Some of the secondary outcomes, e.g. mortality, were rare either by nature or because of the relatively low number of children being seen at some sites. For such scenarios, it is neither easy to transform the time series into a stationary series nor to detect a trend. Depending on the number of zero count months, we either added an indicator variable into the model to account for the zero months effect or we combined data into two-monthly blocks and where possible the trajectory was modelled. Exploratory and sensitivity analyses were also conducted (for details please see Additional Material 3). All analyses were performed using statistical software (R v 3.5.2).

### Qualitative ethnographic evaluation

#### Data generation

In each site, single ward case studies were undertaken to evaluate changes in the paediatric early warning systems. In the pre- and post-implementation phases, data were generated through observation of practice and semi-structured interviews with clinicians, managers, and families. Data were collected and analysed from observations (446 h) and interviews (*n* = 193) across the four sites (Table 3).

**Table 3:** Qualitative data collection for each case study

Site	Pre-implementation data collection (March 2015 to October 2016)	Post-implementation data collection (November 2017 to October 2018)
Site 1	•-54 h of observation •-8 x staff interviews •-13 x family/carer interviews	•-58 h of observation •-13 x staff interviews •-7 x family/carer interviews
Site 2	•-44 h of observation •-13 x staff interviews •-10 x family/carer interviews	•-53 h of observation •-19 x staff interviews •-9 x family/carer interviews
Site 3	•-78 h of observation •-15 x staff interviews •-8 x family/carer interviews	•-51 h of observation •-11 x staff interviews •-10 x family/carer interviews
Site 4	•-70 h of observation •-17 x staff interviews •-7 x family/carer interviews	•-38 h of observation •-23 x staff interviews •-10 x family/carer interviews

Data generation was informed by Translational Mobilisation Theory [5] and Normalisation Process Theory [6], which directed attention to the socio-material network of actors (people, processes, technologies and artefacts) and their relationships in paediatric early warning systems. Observations were conducted at different times of day/night and on different days of the week, including weekends, to ensure that a range of time periods were covered. We focused on what participants did, the tools they used, the concepts they deployed and the factors that facilitated and constrained action [23]. Observations were recorded in low inference field notes which documented in concrete terms what was said and what happened without interpretation and were later word-processed. Interviews were digitally recorded with consent. Field notes, interview transcripts, and documents were uploaded into Computer Supported Qualitative Data Analysis Software (Altas/ti) and coded for ease of retrieval and management.

### Analysis

Concrete descriptions of pre- and post-implementation paediatric early warning systems were developed for each ward and independently assessed by researchers using the PUMA Programme Staff System Assessment Tool (see page 38 of Additional Material 4). Each component of the system was scored from 0 to 10, with 10 indicating the existence of requirements fully aligned with the PUMA Standard and 0 indicating the absence of requirements. Cross-case analysis was undertaken to understand the relationship between the implementation of the PUMA Programme, local context, mechanisms, and outcomes.

### Implementation process evaluation

A parallel process evaluation explored teams’ experiences of implementing the PUMA Programme. The process evaluation focused on the delivery and response to the PUMA Programme, and barriers and facilitators to implementation. Data were generated through observation of facilitated sessions and meetings with site Principal Investigators (PI) (*n* = 5); semi-structured interviews with PIs (*n* = 7), clinical staff and improvement teams; records of telephone facilitation discussions (*n* = 40); analyses of documents - minutes of improvement team meetings and implementation activity.

### Analysis

Concurrent formative process evaluation analysis identified adjustments to the PUMA Programme required to facilitate implementation processes and the necessary modifications undertaken in a process of reciprocal learning between the research team and site PIs. The summative process evaluation analysis was thematic, focusing on delivery and response to the core components of the PUMA Programme, understanding of the OUTCOME approach, barriers to change and implementation, facilitators of change and implementation, and sustainability.

## Results

### Workstream 1: development and implementation of the PUMA Programme

**Fig. 1:**
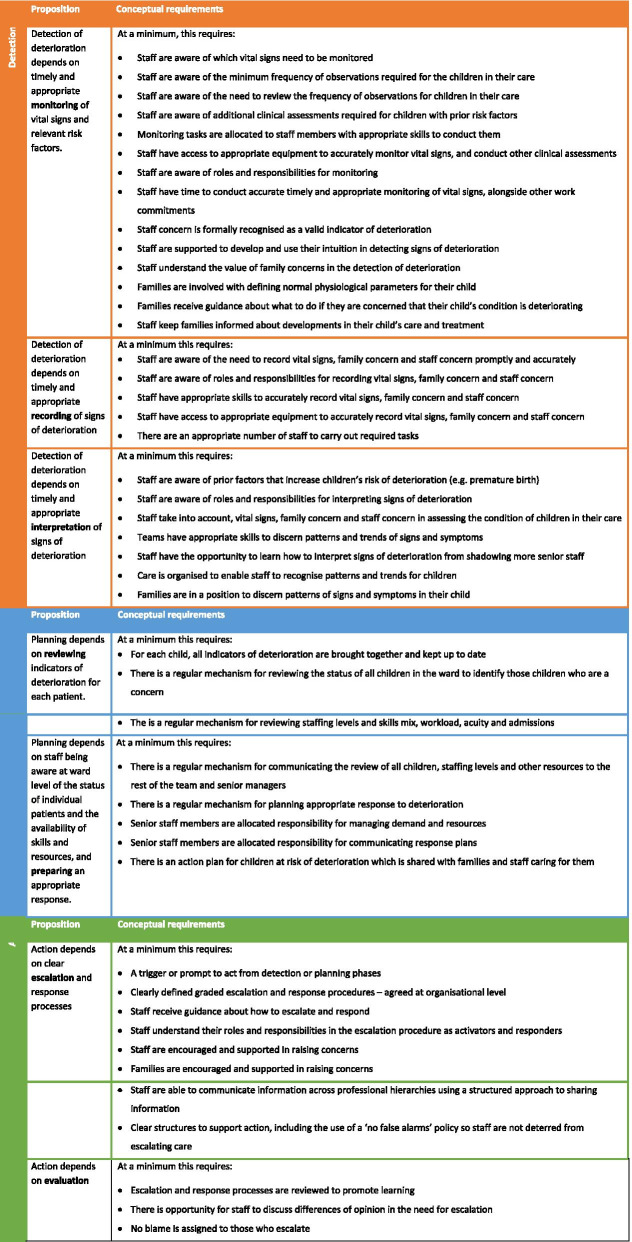
The core components of a paediatric early warning system: the puma standard

**Fig. 2:**
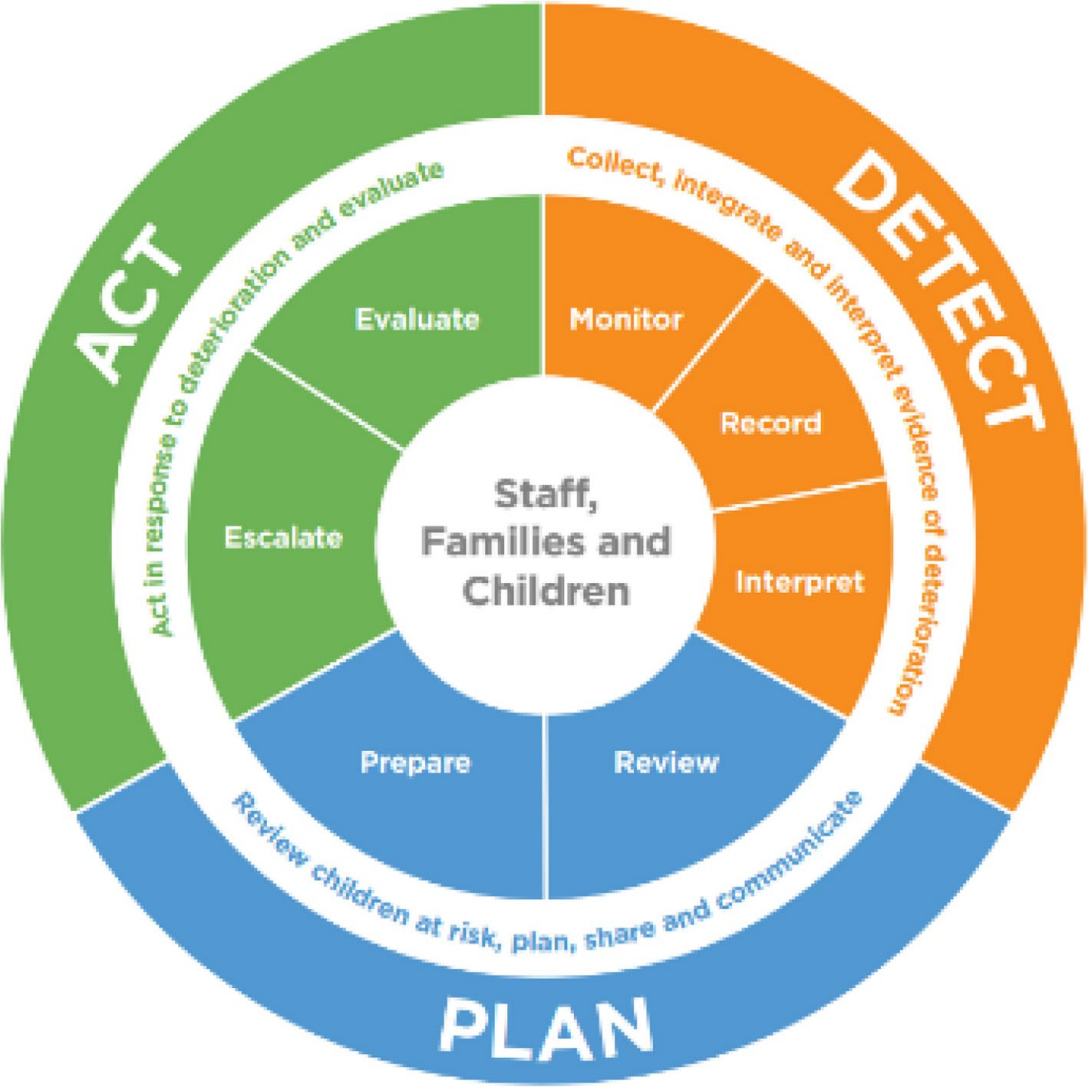
The core components of a paediatric early warning system: the puma wheel

The PUMA Programme comprised of:

- PUMA Standard: an evidence-based and theoretically informed propositional model of a paediatric early warning system organised around the seven functions of an afferent paediatric early warning system (Fig. 1)
- PUMA Wheel: A visual schematic of the PUMA Standard (Fig. 2)
- Paediatric early warning system assessment tools: Staff System Assessment Tool (SSAT) and the Family Feedback Tool (FFF)
- Manualised implementation guidance to support improvement initiatives based on a five-step process (see Additional Material 4):

1. form an improvement team.
2. assess the system.
3. select and plan improvement initiatives.
4. implement and review initiatives.
5. sustain progress.

- Face-to-face structured facilitation and ongoing support (see Table 4).
- Materials to support implementation
- Structured worksheets
- Power point slide pack for local dissemination

**Table 4:** Summary of support and resources provided for each of the five improvement steps

Improvement Step	Facilitated workshop	Materials and resources provided to PIs	Additional facilitation strategies
1. Form an improvement team	‘Set-up’ session	Instructions and worksheets Power Point slides to introduce PUMA to others	Implementation support phone calls between site PIs and PUMA study researcher (offered fortnightly)
2. Assess the system	‘Set-up’ session	Instructions and worksheets PUMA Standard and PUMA wheel PUMA system assessment tools (SSAT and FFT)	Implementation support phone calls between site PIs and PUMA study researcher (offered fortnightly)
3. Select and plan improvement initiatives	‘Action planning’ session	Instructions and worksheets	Implementation support phone calls between site PIs and PUMA study researcher (offered fortnightly)
4. Implement and review initiatives	‘Action planning’ session	Instructions and worksheets	Implementation support phone calls between site PIs and PUMA study researcher (offered fortnightly) Implementation support meetings (phone and face to face) between site PIs and PUMA study team
5. Sustain Progress		Instructions	

The PUMA Programme provided a framework and resources to support local teams to assess their paediatric early warning systems, identify areas for improvement, and decide locally how these would be addressed in each site. It provided a standardised approach across different settings, but still enabled those responsible for implementing interventions to select solutions they believed would work within the local context. The start-up meeting covered OUTCOME principles, the PUMA Standard, the importance of engaging clinical teams in the improvement process, and instruction on how to administer the system assessment tools and collate results. The Action Planning meeting involved a facilitated discussion about initiatives that could be used to address identified areas for improvement. Members of the PUMA study team (1x Consultant Paediatrician and 1x Implementation Scientist) delivered the start-up and action planning sessions and provided on-going support.

**Fig. 3:**
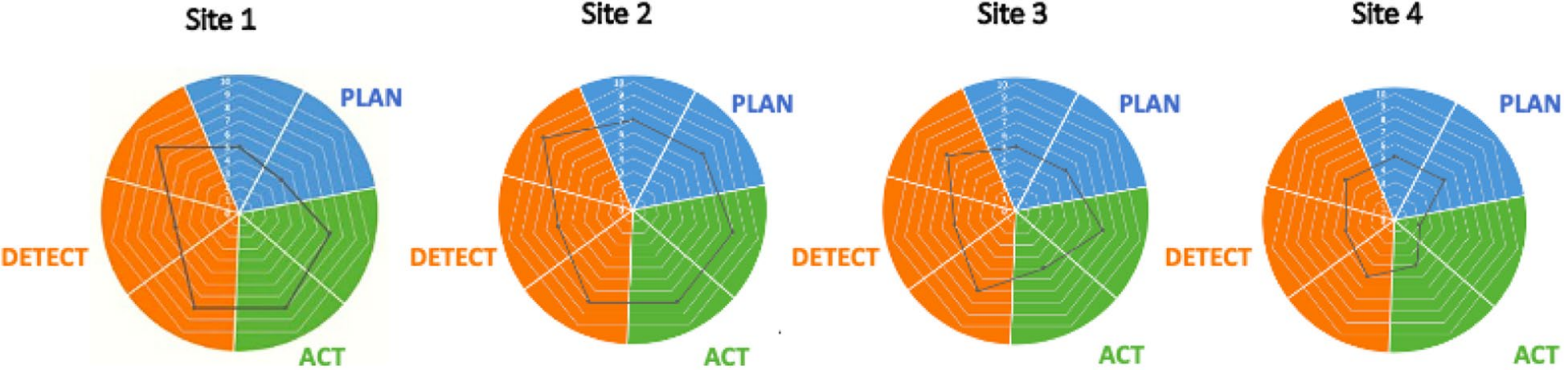
Strengths and weakness of paediatric early warning systems pre-implementation

All sites formed an improvement team of local clinicians and managers, which oversaw system assessment, the identification of weaknesses in the system, and the selection, implementation, and review of improvement initiatives. Assessment of each paediatric early warning system using the PUMA Staff System Assessment Tool revealed how well each system was functioning against the core system components, outlined in the PUMA Standard. Each site had its own fingerprint of strengths and weaknesses [Fig. 3] and contextual differences (patient populations, technological and physical infrastructures, PICU access) which shaped the selection of initiatives and implementation processes. Once sites had identified areas for improvement, they were guided through a process of selecting appropriate improvement initiatives. Local teams led the improvement process in each site.

Findings from the concurrent formative process evaluation led to modifications of the PUMA Programme. The PUMA Standard was refined to provide a more easily accessible version of the original with these changes reflected in adjustments to the PUMA Wheel and Staff System Assessment Tool. The original version of the Family Feedback Tool generated little information of value, with high scores being achieved on all measures. The Family Feedback Tool was subsequently revised and expanded (the new version was co-developed by the PUMA study team and the Patient and Public Involvement Group); an additional number of free-text questions were included, and the language used was clarified.

The PUMA Programme was designed to be implemented by local improvement teams with minimal external facilitation or support. However, over the lifetime of the study this was increased in recognition of the fact that the PUMA Programme resources were being refined and developed in parallel with implementation. Support took the form of individual telephone and/or email-mediated support and site-specific in-person meetings. All PIs either attended or contributed to the face-to-face meetings, and two sites chose to use facilitated telephone calls, during which a PUMA study team member provided tailored support, reviewing, and explaining the intended aims and improvement steps of the PUMA Programme, and assisting with problem-solving in relation to specific initiatives. In response to site PI feedback, additional information was added to the Implementation Guide.

### Qualitative evaluation

#### Improvement initiatives

All sites selected initiatives and made changes to their paediatric early warning systems aligned with the PUMA Standard [Table 5]. Many of the initiatives identified were intended to address issues for which existing interventions were either unavailable or inappropriate, and often involved multiple small interventions that adjusted and harmonised existing processes. In some cases, the team used the PUMA Programme as a vehicle for implementing changes that had been under consideration for some time, for example the new Standard Operating Process for on-call medical team handover at night and the weekend, selected at Site 1. Sites also selected different initiatives to address similar issues. In Site 2 improving awareness of at-risk children was addressed through nursing-medical safety huddles, and in Site 3 through minor adjustment to nursing-medical communications. Teams also found alternatives if initial plans could not be implemented: Site 2 abandoned the development a joint medical-nursing handover sheet but introduced a structured approach to nursing handover.

**Table 5:** Summary of embedded site initiatives against the PUMA Standard

	Proposition	Site 1 initiatives	Site 2 initiatives	Site 3 initiatives	Site 4 initiatives
**DETECT**	Detection of deterioration depends on timely and appropriate **monitoring, recording and interpretation** of vital signs and relevant risk factors.	1. Developed a tool to encourage family engagement 2. Retraining on recognition and response to deterioration including NICE sepsis screening for front-line clinical staff	No relevant initiative	No relevant initiative	1. Observation policy updated and disseminated 2. Posters and cards for staff used to signpost abnormal thresholds for vital signs 3. Observation charts updated to include normal age-related thresholds disseminated 4. Inventory of equipment conducted
**PLAN**	Planning depends on **reviewing** indicators of deterioration for each patient, staff being aware at ward level of the status of individual patients and the availability of skills and resources, and **preparing** an appropriate response.	3. Implemented standard operating procedures (SOP) for out of hours working for on-call medical teams – prioritising sickest children (hospital-wide)	1. Initially planned to introduce second daily huddle, but it was not deemed possible. More frequent telephone calls between the ward and Paediatric Assessment Unit (PAU) introduced and the two areas now share a rotation of Band 6 nurses. A safety huddle takes place at 9 am on the main ward now seems to have taken on the momentum for addressing what the second daily huddle initially set out to do. 2. Initially planned joint handover sheets, using Situation Background Assessment Recommendation (SBAR), but was not deemed possible. Nurses’ handover sheet changed to SBAR.	1. Introduced site board to display ‘4Ss’ (sickest patients, bed status, safeguarding issues and staffing). 2. Senior nurses now phone through to doctors’ handover if they have any concerns about a particular patient	5. Plans to establish a staff training course on situational awareness were amended; situational awareness now included in statutory training days
**ACT**	Action depends on clear **escalation** and response and **evaluation** processes.			3. Introduction of new escalation policy	6. Escalation policy reviewed and disseminated

### Implementation trajectories

There were different implementation trajectories in each site, reflecting several factors. First, it depended on the specific initiatives selected and whether these were relatively quick fixes or minor adjustments to existing processes, or whether they required more investment in development work, such as agreeing a new escalation policy (Sites 3 & 4). Second, it reflected the scale of work undertaken to embed the interventions, which related to organisational size and complexity. With only one ward, implementation at Site 2 was relatively straightforward. For the larger sites, the process was more difficult and required extensive engagement work and decisions about which initiatives should be implemented across the whole organisation, and which could be left to the local determination of wards. Third, it reflected the capacity of the improvement teams. The single site PI in Site 4 provided strong leadership for implementation, and delegated responsibility for leading on specific initiatives to identified individuals. But an unplanned absence from work led to a loss of momentum during the implementation phase, highlighting the potential risks of investing leadership exclusively in one person. In Site 3, staff turnover made sustaining an improvement team challenging, and most of the initiatives were progressed exclusively by the site PIs. Membership of the improvement team in Site 1 also fluctuated, and, at this site, the energy of PIs was taken up by the requirement to oversee large-scale changes relating to a regulatory requirement. In Site 2, there was a clearly defined implementation/improvement team that took on responsibility for different initiatives, which meant that some of the initiatives were implemented quickly. Fourth, it reflected wider organisational support for the improvement programme. Only Site 1 had a high level of organisational support for their initiatives, as these aligned with regulatory mandated changes arising from a critical incident.

### Changes to paediatric early warning systems

All sites brought about improvements in reviewing sick children and planning for action so that there was a shared understanding of children at risk. Several sites addressed equipment shortages (Sites 3 & 4). All sites implemented initiatives to involve parents more systematically in detecting and acting upon deterioration but with limited success.

Some initiatives were implemented but never embedded in practice and some initiatives were never implemented (for a summary of initiatives proposed, implemented, and embedded see Additional Material 5). In several cases, initiatives required the negotiation of organisational barriers beyond the sphere of influence of improvement teams. For example, in Sites 2 and 4 interventions to support professional development were not implemented as staff could not be released from clinical areas. Implementing all selected initiatives was not possible within available timescales.

At the close of the study improvement work continued in several sites.

### Paediatric early warning system dynamics

The study findings highlighted the dynamic qualities of paediatric early warning systems. For example, across the sites, improvement initiatives strengthened some components of the system, but weakened others. For example, the introduction of an electronic early warning system in Site 2 strengthened medical access to patient data, but disrupted nursing work as there were insufficient computers available to allow nurses to enter vital signs, leading to a delay between monitoring and recording activity. Finally shifting wider contextual factors impacted on the functioning of early warning systems in all sites. For example, Site 4 was involved in wider organisational restructuring which impacted on governance approval processes for a new escalation policy, and a critical incident in Site 1 led to a series of hospital-wide mandated changes aligned with the PUMA Standard, following recommendations in a Care Quality Commission (CQC) report, with a level of organisational sponsorship not apparent in the other sites [For a summary of changes in the study sites, see Table 6].

**Table 6:** Contextual factors impacting on paediatric early warning systems during implementation

	Site 1	Site 2	Site 3	Site 4
**Key organisational-level changes to the paediatric early warning system**	·Response to critical incident and CQC inspections led to several trust-mandated changes (see non-PUMA initiatives)	Nothing to report	Study Chief Investigator with senior clinical position at site left post (no longer present as reminder to frontline staff of PUMA/associated initiatives) · High level of staff turnover · Changes to PICU and HDU admissions thresholds – increased likelihood of admission/referral	Hospital involved in wider organisational-level restructuring; some impact on initiatives which required governance and institutional approvals.
**Key ward-level changes to the paediatric early warning system**	· Increase in number of qualified nursing staff · Loss of some experienced nursing staff · Introduction of consistent 24/7 band 6/shift coordinator cover · Additional, more effective mobile computers · 2 senior nurses training to become ANPs	· New manager joins ward and reduces HDU transfers, through greater focus on needs of individual patient rather than care plan. Many patients who would previously have been admitted to HDU cared for on the ward	· Nursing handover/team organisation changed; nurses allocated to one section of ward, received handover for patients in that section only · ANPs no longer included within medical team · Medical team increased from 8 to 10-person rota, increased capacity to cope with sickness/training absence · Improvements to monitoring equipment; additional Optiflows & saturation monitors, central monitoring station.	Nothing to report
**Additional changes during post-implementation period**	Nothing to report	· Loss experienced nursing staff · Increase in number of band 6 nurses · Increased use of agency staff	Nothing to report	Nothing to report

**Fig. 4:**
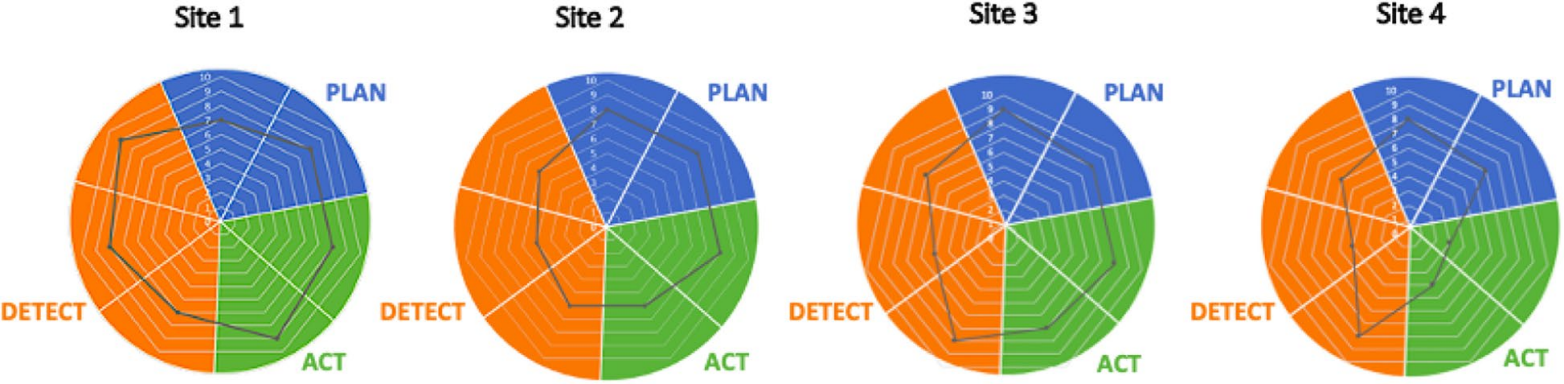
Strengths and weakness of paediatric early warning systems post-implementation

The paediatric early warning system in each site was assessed in the post-implementation period and demonstrated improvements in most components of the system (Fig. 4). Table 7 summarises the positive (+) and negative (−) changes to the paediatric early warning systems in each site.

**Table 7:** Positive and negative changes to paediatric early warning system - post implementation

	Site 1	Site 2	Site 3 (Two medical wards only)	Site 4
Detect	**+** Electronic PTTT normalised **+** Additional computers, more timely data entry **+** SEPSIS-6 pathway normalised **+** All senior nursing staff APLS trained **+** All staff PEWS/SEPSIS-6 trained **+** New escalation policy in use. Provided explicit guidance on: shift coordinator’s seniority and position within escalation pathway; care requirements and typical vital signs of cardiac patients; when/how to escalate based on PTTT score and key vital signs **+** Newly-qualified staff valued PTTT score **-** Some loss of senior nursing staff	**-** New electronic PTTT: junior staff struggled to recall schedule of observations; able to view recent vital signs data only; lack of computers prevented timely data input. **-** Loss of experienced nursing staff, increased need for support of junior staff.	**+** small number of SHINE posters distributed in general medical wards only **+** improvements to monitoring equipment **-** SHINE posters not utilised by families/staff	**+** inventory of equipment conducted; access to monitoring equipment improved, mobile observation trolleys appropriately stocked. **+** laminated observation/escalation guidelines included within every patient file **+** posters/cards provided easily-accessible information on vital signs parameters, cards frequently and routinely used **+** colour-coded observations chart highlighted abnormal vital signs thresholds **+** storage and management of patient files improved **-** Low staff awareness of formal observation/escalation policy
Plan	**+** Safety huddle introduced; nursing team situational awareness improved **+** More regular shift coordinator attendance at evening board round **-** Challenges with ward round remained: some conducted away from patient bedside, shift coordinators ‘pulled away’ due to competing demands/patient caseload	**+** New medical handover sheet included all patients, increased awareness **+** Safety huddle allowed rapid identification of children at risk of deterioration, aided communication of key messages, bed management issues and safeguarding concerns. Enhanced awareness of ward/patient status, facilitated appropriate medical review, introduced and normalised language of ‘watcher’ across all staff groups **+** New nursing handover utilised SBAR; quicker, more succinct **+** Electronic scoring system increased doctors’ ability to review patients away from the ward, led to closer working between ward and PAU. **+**Ward manager/deputy attended post ward-round meetings **-** Ward manager less frequent attendance at medical handover, loss of communication	**+** nurses introduced whiteboard to help ensure that content of safety briefing remained clear/concise in general medical wards only **+** shorter handover improved nurses’ concentration **+** new ‘4Ss whiteboard’ utilised during doctors’ handover improved shared situational awareness; information on bed status and sickest patients delivered by senior nurse immediately prior to handover, improved planning.	**+** Increased situational awareness amongst team; handover content improved (5Ss utilised). **+** At-risk children consistently given ‘watcher’ status **+** Whiteboard updated and utilised regularly by nursing and medical staff
Act	**+** Inter-team communication improved via consistent day/night shift coordinator cover and clarification of escalation pathway. **+** Senior nursing staff updated training on response to deterioration (via APLS)	**-** New PTTT: information on escalation pathway less accessible **-** Increased use of agency staff, unfamiliar with escalation procedure **-** Newer/less experienced staff reluctant to escalate directly to medical team **-** Doctors review patient status away from ward, less able/likely to respond to nurse concern	**+** Staff had high level of awareness of escalation process in medical wards only **+** Appointment of new PICU consultants led to changed thresholds, increased likelihood of admission/acceptance.	**+** quicker escalation/transfer of at-risk children to HDU beds

### Quantitative evaluation

**Fig. 5:**
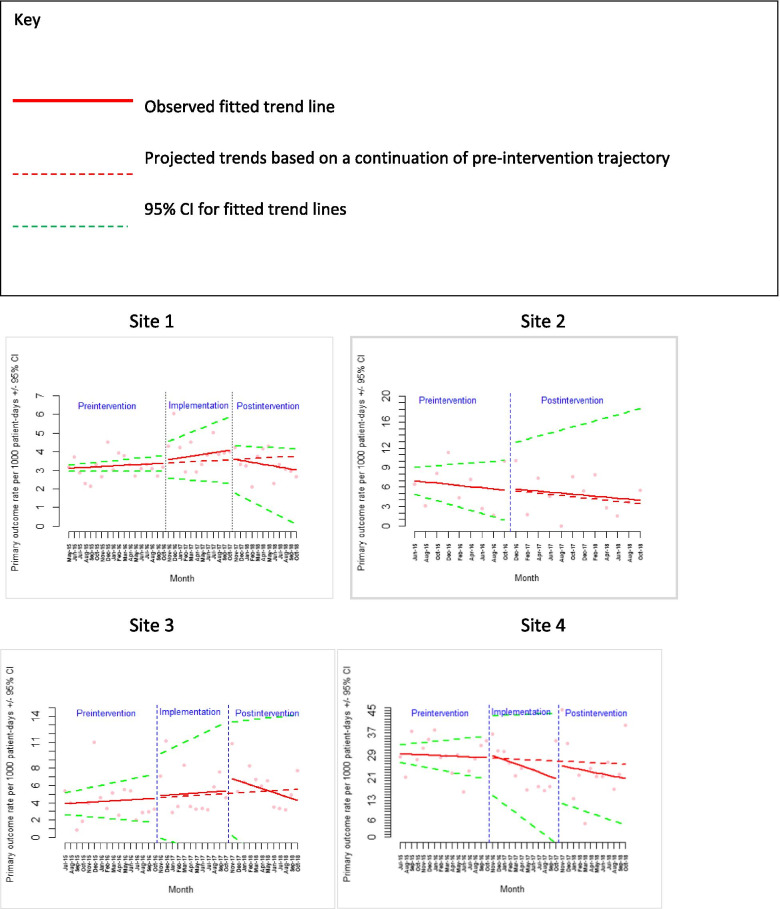
Scatter plots for primary outcome in each of the four sites with fitted line from segmented linear regression

Data were collected on eight outcome measures for 42 months. Modelling the impact of the PUMA Programme on quantitative outcomes was challenging. Although mortality, cardiac arrest, respiratory arrest, and unplanned admission to PICU/HDU have been commonly used in combination to assess paediatric early warning systems, in practice they occur relatively infrequently, and this was apparent in the smaller general hospitals with fewer patients, which are rarely included in this type of study. Figure 5 shows the fitted trend lines for pre-intervention, implementation, and post-intervention rates of adverse events, per 1000 patient bed-days, with estimates and *p*-values shown in Tables 8, 9, 10 and 11. Overall, they show a mixed picture across the four sites, with wide confidence intervals illustrating the challenges in assessing trends in outcomes with low event rates. For Site 2 the numbers were so low, that it was not possible to model all three periods and therefore a two-stage model with implementation and post-intervention combined.

**Table 8:** Estimates from segmented linear regression for adverse events in Site 1

Outcome	Estimate, ß (95% CI)	*P* Value	Interpretation
Adverse events			
Intercept	3.08 (2.93, 3.24)	< 0.00001	
Pre-intervention trend	0.02 (0.00, 0.03)	0.04	Adverse events were very gradually but significantly increasing during this period. Given the low overall rates the clinical impact of this increase is difficult to determine.
Change in slope (implementation period vs. pre-intervention period)	0.03 (−0.03, 0.09)	0.29	There was a trend towards an increasing rate of adverse events (against the expected trend) but this was not significant. The wide confidence intervals mean the trend could have been in either direction should a greater sample size have been available.
Immediate change in level (implementation period vs. pre-intervention period)	0.15 (− 0.34, 0.64)	0.55	
Change in slope (post-intervention period vs. implementation period)	-0.09 (−0.15, − 0.05)	< 0.001	Adverse event rates decreased by nearly 10% in this period, compared to the implementation period, which was statistically significant.
Immediate change in level (post-intervention period vs. implementation period)	−0.43 (−1.03, 0.17)	0.16

**Table 9:** Estimates from segmented linear regression for adverse events in Site 2

Outcome	Estimate, ß (95% CI)	*P* Value	Interpretation
Adverse events			
Intercept	3.08 (2.93, 3.24)		
Pre-intervention trend	−0.17 (− 0.49, 0.17)	0.29	There is a trend (non-significant) for reducing events but the paucity of them occurring (in relation to raw numbers) makes it difficult to draw concrete conclusions.
Change in slope (implementation period vs. pre-intervention period)	0.02 (−0.30, 0.33)	0.98	The trend does not appear to change but the confidence limits around this are large.
Immediate change in level (implementation period vs. pre-intervention period)	0.29 (−1.74, 2.32)	0.78

**Table 10:** Estimate from segmented linear regression for adverse events in Site 3

Outcome	Estimate, ß (95% CI)	*P* Value	Interpretation
Adverse events			
Intercept	3.27 (2.12, 4.42)		
Pre-intervention trend	0.04 (−0.06, 0.15)	0.42	There is a trend towards increasing event rates although this is not significant.
Change in slope (implementation period vs. pre-intervention period)	0.01 (−0.16, 0.18)	0.92	The event rate doesn’t change but given the wide confidence intervals it is difficult to be precise about whether this is a true effect.
Immediate change in level (implementation period vs. pre-intervention period)	0.21 (−1.55, 1.97)	0.81	
Change in slope (post-intervention period vs implementation period)	−0.27 (− 0.47, − 0.07)	0.01	The trend significantly reduced over this period (although the overall number of events per patients day increases)
Immediate change in level (post-intervention period vs. implementation period)	1.98 (−0.22, 4.18)	0.09

**Table 11:** Estimates from segmented linear regression for adverse events in Site 4

Outcome	Estimate, ß (95% CI)	*P* Value	Interpretation
Adverse events			
Intercept	29.69 (26.89, 32.49)		
Pre-intervention trend	−0.10 (−0.40, 0.21)	0.55	There was no apparent significant trend in the overall adverse event rate.
Change in slope (implementation phase vs pre-intervention phase)	−0.64 (−1.15, − 0.13)	0.02	There was a significant deviation in the event rate during the implementation phase which probably represents a real clinical impact.
Immediate change in level (implementation period vs. pre-intervention period)	1.57 (−4.05, 7.18)	0.59	
Change in slope (post-intervention phase vs implementation phase)	0.32 (−0.29, 0.93)	0.31	This trend was maintained but was not significantly different from the implementation phase.
Immediate change in level (post-intervention period vs. implementation period)	0.32 (−0.29, 0.93)	0.31	

ITS and qualitative findings were triangulated for each site. Site 1 implemented multiple organisational level changes aligned with the PUMA Standard, mandated in response to a critical Care Quality Commission (CQC) report, which were associated with significant improvements in adverse event trends in the post-intervention phase relative to implementation phase (ß = -0.09 (95% CI: − 0.15, − 0.05); *p* = < 0.001) [Fig. 5]. Several other quantitative findings appeared to relate to qualitative data. Site 4 implemented several organisational level system changes at an early stage in the study, which coincided with a decreased slope in adverse event rates during the implementation phase relative to the pre-intervention trend (ß = -0.64 (95% CI: − 1.15, − 0.13); *p* = 0.02). Site 2 introduced a safety huddle and electronic recording, which strengthened some aspects of the local system and weakened others. There was no significant ‘interruption’ to the adverse event rate after implementing the PUMA Programme (ß = 0.02 (95% CI: − 0.30, 0.33); *p* = 0.98), which continued to gently decrease in line with pre-intervention trends. Very early in the pre-intervention period, a new ward manager implemented a strategy to reduce HDU transfers, which may have contributed to declining event rates over the study period. Site 3 made several improvements in certain wards, but no organisational level changes. There was a significant downward slope in the adverse event rate trends observed in the post-intervention phase relative to the implementation period (ß = -0.27 (95% CI: − 0.47, − 0.07); *p* = 0.01), but the overall event rate did not decrease. This mixed pattern of findings may have been clearer if we had continued to collect data over a longer period.

### Implementation process evaluation

Improvement team members embraced the OUTCOME principles underpinning the PUMA Programme to different degrees, but all considered the system assessment process to have value. Discussing results and agreeing how to rank their system against the PUMA Standard was regarded as important. They also proposed that the system assessment made the process of improvement easier, as it allowed them to engage staff groups from an early stage, providing on-the-ground expertise and evidence of areas for improvement:

It wasn’t just [site leads] plucking out what did we want to take forward, this is what everybody on the team has said needs improving.

Yet while teams reported strong ownership of the improvement process, they required encouragement to develop local approaches to system problems rather than reaching for off-the-shelf solutions. Teams did not have specialist quality improvement skills nor dedicated time to undertake improvement work, which impacted on progress and team stability. Implementation was challenging in all sites and highlighted the need for organisational sponsorship for improvement programmes.

## Discussion

The PUMA Programme was developed to facilitate local improvements to paediatric early warning systems oriented to a common standard. Cumulative research highlights the need for a systems approach to improve the detection and response to deterioration in hospitalised patients. Hitherto no frameworks have existed to support system level improvement.

Our findings highlight the impacts of the PUMA Programme on clinical outcomes when system level change is organisationally mandated (Site 1) but also the challenges of locally led improvement in the absence of organisational sponsorship (Sites 2, 3 and 4). While the PUMA Programme was designed to support context-appropriate approaches to improving paediatric early warning systems, the findings point to several areas where common standards have value. First, clinical expertise is a component of any paediatric early warning system, and staff turnover has potentially disruptive effects. Several sites (2 and 4) identified the need for education and training in their improvement initiatives, yet it was only in Site 1 where training was organisationally mandated that these initiatives became embedded in practice and staff were released from clinical work to attend. Professional development should be a critical component of all systems and mandated multidisciplinary training considered. Second, in several sites a lack of access to appropriate equipment was identified as impacting negatively on the system – this ranged from appropriate monitoring equipment to access to computers for data entry. A process to ensure the correct equipment is available and functioning is a prerequisite of any paediatric early warning system irrespective of the singular features of local context. Third, all sites recognised the importance of involving parents in detecting and acting on deterioration but had limited success in implementing changes to the system. Parental involvement in the detection of deterioration is difficult to address outside of wider strategies to facilitate parental involvement in children’s care. Fourth, by observing over time, the study highlighted the dynamic qualities of paediatric early warning systems, the impacts of internal and external contextual changes, and the distributed costs and benefits of change for participants. This points to the need for regular assessment of system functioning as part of a continuous improvement culture.

To our knowledge no studies have robustly assessed the impact of interventions to improve paediatric early warning *systems*. While a large randomised controlled trial of a specific score has recently been reported, this focused on patient outcomes rather than wider system change [24]. Most other studies have examined the feasibility or validation of scores, rather than systems, and have been heterogeneous in their design and reproducibility [2]. Our results are in keeping with other cohort studies [25] which demonstrated improvements over time regardless of interventions. The robust mechanism with which we looked at a variety of outcomes also meant that some of the gains seen in single outcome measure studies were not realised [26].

Determining the impact of the PUMA Programme using quantitative measures of in-patient deterioration was challenging. First, implementation was a process rather than a discrete event, creating challenges for the ITS. The ‘implementation period’ was conceptualised as 12 months for analytic purposes, in practice this likely varied between sites and was less well defined than in some intervention studies. Second, the commissioning brief related to interventions to reduce mortality and so our primary outcome (‘adverse events’) was a composite measure that included mortality and other related clinical metrics. The decision to use a composite metric for the primary outcome mirrors other single-site effectiveness studies of paediatric early warning system interventions [24]. It was largely a pragmatic decision, reflecting the low event rates of individual clinical outcomes such as mortality and arrests in hospitalised children. Even using this composite outcome, incorporating unplanned HDU and PICU transfers, we observed several zero months in our smallest DGH. Low event rates for key outcome metrics in DGHs point to the difficulty in assessing changes over time in smaller hospitals, and a key reason paediatric early warning systems research is dominated by studies conducted in large specialist centres.

Mortality is significantly lower in children than in adult in-patient settings, [27] here is an ongoing decline in child mortality, [25] and even in-patient deterioration is a relatively infrequent occurrence [24]. Analytic approaches to rare event modelling, such as Bayesian Belief Networks, could be adapted from other fields to support the focus on preventing these events, however a clear assessment of potential is required. The literature on rare events requires clear causal pathways and the complexity of child deterioration and death may not be amenable to such approaches. New methodologies are required.

Including HDU and PICU transfers as markers of in-patient deterioration is common in the literature, but not without difficulty. As we demonstrated in the qualitative work, use varies in response to other system pressures or changes in clinical practices of senior staff. Our findings lend weight to debates about the appropriateness of downstream individual level outcome measures in this field and point to the need to reach agreement on up-stream indicators of paediatric early warning system performance. These may include inter alia measures of process, culture, parental involvement, and staff situational awareness. While these are worthy of future study, at the inception of this study, adequate up-stream indicators of paediatric early warning system performance did not exist. The PUMA Standard offers a valuable framework for progressing the development of alternative metrics, through consensus methods, such as a Delphi Study.

## Conclusions

System level change to improve paediatric early warning systems can bring about positive impacts on clinical outcomes, but in paediatric practice, where the patient population is smaller and clinical outcomes event rates are low alternative outcome measures are required to support research and quality improvement beyond large specialist centres, and methodological work on rare events is indicated.

Paediatric early warning systems are dynamic, and their functioning is influenced by wider contextual changes. The PUMA Programme offers structures to support regular assessment, learning and local improvement.

The PUMA Programme offers a new approach to improving the detection and response to deterioration in the in-patient paediatric context by focusing on the whole system. With appropriate organisational support, the PUMA Programme has value as a framework for continuous improvement of paediatric early warning systems across diverse national and international contexts, including developing healthcare systems. The OUTCOME approach to improvement, has the potential to be used more widely.

## Supplementary Information

**Additional file 1: Table 1**. Summary of theories that inform OUTCOME.

**Additional file 2.** Summary of outcomes used as proxies for in-patient deterioration.

**Additional file 3.** Summary of exploratory and sensitivity analyses.

**Additional file 4.**

**Additional file 5.** Summary of paediatric early warning system improvement initiatives across all case studies.

## Data Availability

The quantitative datasets used and/or analysed during the current study are available from the corresponding author on reasonable request.

## Abbreviations

EWS: Early Warning Score
HDU: High Dependency Unit
ITS: Interrupted Time Series
NPT: Normalisation Theory
PAU: Paediatric Assessment Unit
PICU: Paediatric Intensive Care Unit
PTTT: Paediatric Track and Trigger Tool
PUMA: Paediatric early warning system: Utilisation and Mortality Avoidance
SOP: Standard Operating Procedure
TMT: Translational Mobilisation Theory
TTT: Track and Trigger Tool

## References

[1] Wolfe I, Cass H, Thompson MJ, Craft A, Peile E, Wiegersma PA (2011). Improving child health services in the UK: insights from Europe and their implications for the NHS reforms. BMJ.

[2] Trubey R, Huang C, Lugg-Widger FV, Hood K, Allen D, Edwards D (2019). Validity and effectiveness of paediatric early warning systems and track and trigger tools for identifying and reducing clinical deterioration in hospitalised children: A systematic review. BMJ Open.

[3] Jacob N, Moriarty Y, Lloyd A, Mann M, Tume LN, Sefton G (2019). Optimising paediatric afferent component early warning systems: a hermeneutic systematic literature review and model development. BMJ Open.

[4] Thomas-Jones E, Lloyd A, Roland D, Sefton G, Tume L, Hood K (2018). A prospective, mixed-methods, before and after study to identify the evidence base for the core components of an effective Paediatric early warning system and the development of an implementation package containing those core recommendations for use in et. BMC Pediatr.

[5] Allen D, May C. Organizing Practice and Practicing Organization: An Outline of Translational Mobilization Theory. SAGE Open. 2017. 10.1177/2158244017707993.

[6] May CR, Mair F, Finch T, MacFarlane A, Dowrick C, Treweek S (2009). Development of a theory of implementation and integration: Normalization Process Theory. Implement Sci.

[7] Langley G, Moen RD, Nolan KM, Nolan TW, Norman CL, Provost LP. The Improvement Guide: Wiley; 2009.

[8] May C, Finch T, Mair F, Ballini L, Dowrick C, Eccles M (2007). Understanding the implementation of complex interventions in health care: the normalization process model. BMC Health Serv Res.

[9] Dixon-Woods M, Leslie M, Tarrant C, Bion J (2013). Explaining Matching Michigan: An ethnographic study of a patient safety program. Implement Sci.

[10] Leape LL (2014). The Checklist Conundrum.

[11] May CR, Johnson M, Finch T. Implementation, context and complexity. Implement Sci. 2016;11:141. 10.1186/s13012-016-0506-3. 10.1186/s13012-016-0506-3 PMC5069794 27756414

[12] Braithwaite J (2018). Changing how we think about healthcare improvement. BMJ.

[13] Reed JE, Card AJ (2016). The problem with plan-do-study-act cycles. BMJ Qual Saf.

[14] Jagt EW (2013). Improving pediatric survival from resuscitation events: the role and organization of hospital-based rapid response systems and code teams. Curr Pediatr Rev.

[15] Stephens TJ, Peden CJ, Pearse RM, Shaw SE, Abbott TEF, Jones EL, et al. Improving care at scale: process evaluation of a multi-component quality improvement intervention to reduce mortality after emergency abdominal surgery (EPOCH trial). Implementation Sci. 2018;13:142. 10.1186/s13012-018-0823-9. 10.1186/s13012-018-0823-9 PMC6233578 30424818

[16] Berg M, Hughes JF, Prinz W, Rodden T, Schmidt K (1997). On distribution, drift and the electronic medical record: some tools for a sociology of the formal. Proceedings of the fifth European conference on computer-supported cooperative work.

[17] Zhang F, Wagner AK, Ross-Degnan D (2011). Simulation-based power calculation for designing interrupted time series analyses of health policy interventions. J Clin Epidemiol.

[18] Tibballs J, Kinney S (2009). Reduction of hospital mortality and of preventable cardiac arrest and death on introduction of a pediatric medical emergency team. Pediatr Crit care Med a J Soc Crit Care Med World Fed Pediatr Intensive Crit Care Soc.

[19] Tibballs J, Kinney S, Duke T, Oakley E, Hennessy M (2005). Reduction of paediatric in-patient cardiac arrest and death with a medical emergency team: preliminary results. Arch Dis Child.

[20] Gottman J. Time-series Analysis. A Comprehensive Introduction for Social Scientists. By J. M. Gottman. (Pp. 400; illustrated; £18.50.) Cambridge University Press: Cambridge. 1981. Psychol Med [Internet]. 2009/07/09. 1982;12(3):682. Available from: https://www.cambridge.org/core/article/timeseries-analysis-a-comprehensive-introduction-for-social-scientists-by-j-m-gottman-pp-400-illustrated-1850-cambridge-university-press-cambridge-1981/978C3168F2DB87E154E599F81BA2AF7E

[21] Nelson BK (1998). Statistical methodology: V. time series analysis using autoregressive integrated moving average (ARIMA) models. Acad Emerg Med.

[22] Lopez Bernal J, Soumerai S, Gasparrini A (2018). A methodological framework for model selection in interrupted time series studies. J Clin Epidemiol.

[23] Spradley JP (1980). Participant observation.

[24] Parshuram CS, Dryden-Palmer K, Farrell C, Gottesman R, Gray M, Hutchison JS (2018). Effect of a pediatric early warning system on all-cause mortality in hospitalized pediatric patients: the EPOCH randomized clinical trial. JAMA.

[25] Joffe AR, Anton NR, Burkholder SC (2011). Reduction in hospital mortality over time in a hospital without a pediatric medical emergency team: limitations of before-and-after study designs. Arch Pediatr Adolesc Med.

[26] Tucker KM, Brewer TL, Baker RB, Demeritt B, Vossmeyer MT (2009). Prospective evaluation of a pediatric inpatient early warning scoring system. J Spec Pediatr Nurs.

[27] Roland D (2017). Paediatric early warning systems: myths and muses. Paediatr Child Health (Oxford).

