# Supplementary material for: Development, implementation and evaluation of an evidence-based paediatric early warning system improvement programme: the PUMA mixed methods study
Source: BMC Health Serv Res. 2022 Jan 2;22:9. doi: 10.1186/s12913-021-07314-2 (PMC8722056; doi:10.1186/s12913-021-07314-2)
Supplement: Supplementary file 1 — Additional file 1: Table 1. Summary of theories that inform OUTCOME. [file 12913_2021_7314_MOESM1_ESM.docx]

Additional MAterial 1

**Table 1: summary of theories that inform OUTCOME**

|  | **Overview** | **Core Constructs** | **Application** |
| --- | --- | --- | --- |
| **Translational Mobilisation Theory (TMT)** | TMT is a theory of collective action, which focuses on the goal of a particular system of work, the elements of context that are most salient to enacting the goal, and the mechanisms by which that may be achieved. | *Project*: What is done in collective action  *Strategic Action Field*: Where collective action is done  *Mechanisms*: How collective action is done | TMT is a relatively new theory and this is the first time it has been deployed for quality improvement purposes, where it provides a logical scaffolding to link theories and insights from Implementation Science and Quality Improvement. |
| **Normalisation Process Theory (NPT)** | NPT is a theory of implementation which shares the domain assumptions of TMT and may be used to inform the support required to enable context-appropriate solutions to be selected and embedded. | *Coherence*: Agreeing on the premise and value of operationalizing a new set of practices  *Cognitive participation*: Building and sustaining a community around the new set of practices  *Collective Action*: Working collectively to implement a new set of practices  *Reflexive monitoring*: Reviewing and appraising the new set of practices | NPT is traditionally used by Implementation Science researchers and focuses on the work that is done around an intervention or new set of activities to embed them into routine practice. |
| **Model for Improvement** | The model outlines five steps for improvement: forming the team, setting aims, establishing measures, selecting changes and testing changes using Plan Do Study Act cycles. | Three fundamental questions form the foundation of this approach:  (1) What are we trying to accomplish?  (2) How will we know that a change is an improvement?  (3) What changes can we make that will result in improvement? | The Model for Improvement is traditionally used in Quality Improvement programmes as a framework for developing, testing and implementing changes. |
