# Supplementary material for: Development, implementation and evaluation of an evidence-based paediatric early warning system improvement programme: the PUMA mixed methods study
Source: BMC Health Serv Res. 2022 Jan 2;22:9. doi: 10.1186/s12913-021-07314-2 (PMC8722056; doi:10.1186/s12913-021-07314-2)
Supplement: Supplementary file 2 — Additional file 2. Summary of outcomes used as proxies for in-patient deterioration. [file 12913_2021_7314_MOESM2_ESM.docx]

Additional Material 2: Summary of outcomes used as proxies for in-patient deterioration

Summary of outcomes used as proxies for in-patient deterioration

| **Outcome** | **Agreed definition** |
| --- | --- |
| Mortality | All-cause mortality among any child admitted to the hospital children’s ward, HDU or PICU.  Excludes:   - Children brought in dead to A&E |
| Cardiac arrest | A child admitted to the hospital’s children’s ward or HDU who subsequently had a cardiac arrest. |
| Respiratory arrest | A child admitted to the hospital’s children’s ward or HDU who subsequently had a respiratory arrest. |
| Unplanned admission to PICU | A child who has an unplanned admission to a PICU bed from the hospital’s children’s ward(s) or HDU.  Excludes:   - Children admitted directly to PICU, either from A&E within the hospital or from another hospital - Admissions from outside of the ward (e.g., A&E, theatre) - Admissions that were elective or planned in advance - Admissions directly from operating room/sleep lab - Admissions where need for ICU care is attributed to need to recover from sedation |
| Unplanned admission to HDU | A child who has an unplanned admission to a designated/funded HDU bed from the hospital’s children’s ward(s).  Excludes:   - Children admitted directly to HDU from A&E or from another hospital - Children admitted to HDU from PICU - Admissions from outside of the ward (e.g., A&E, theatre) - Admissions that were elective or planned in advance - Admissions directly from operating room/sleep lab - Admissions where need for HDU care is attributed to need to recover from sedation |
| PICU reviews | A child admitted to the hospital’s children’s ward or HDU who is reviewed by an internal member of PICU staff (Tertiary) or who is the subject of a phone call to external PICU (DGH) for advice, regardless of whether review leads to a PICU admission. |
| Other medical emergency requiring immediate assistance | A child admitted to the hospital’s children’s ward or HDU who subsequently required an arrest call/code for any emergency other than a cardiac or respiratory arrest. |
|  |  |
| Non-ICU patient days (16 years or under) | The total number of occupied bed days over the month.  Calculated by daily census (typically midnight) of inpatients on the hospital’s children’s ward(s) and HDU.  Excludes children over 16 and children on ICU wards. |
