# Supplementary material for: Development, implementation and evaluation of an evidence-based paediatric early warning system improvement programme: the PUMA mixed methods study
Source: BMC Health Serv Res. 2022 Jan 2;22:9. doi: 10.1186/s12913-021-07314-2 (PMC8722056; doi:10.1186/s12913-021-07314-2)
Supplement: Supplementary file 3 — Additional file 3. Summary of exploratory and sensitivity analyses. [file 12913_2021_7314_MOESM3_ESM.docx]

Additional MAterial 3: Summary of exploratory and sensitivity analyses

**Exploratory analysis**

Primary outcomes from each hospital were analysed using common approach, i.e. data from implementation period was excluded from the analysis and changes in level and slope were examined by comparing data from the pre- and post-implementation period. Additionally, we examined changes in the level and slope of trajectory by fitting the ITS model at each individual month of the implementation period; resulting in 12 separate models per site. The reason for the latter analysis was to assess the pattern of changes in level and slope from the start of implementation phase until the end, given the potential for the different local initiatives to exert their effects over different time periods in different sites.

**Sensitivity Analysis**

As the outcomes are counts, segmented Poisson regression might be considered appropriate. Therefore, segmented Poisson regression was also used to analyse primary outcomes as a sensitivity analysis. Zero inflated segmented Poisson regression was used if there are many zero counts in primary outcome. Of note, Poisson regression has memoryless characteristic which means there is no need to model residuals to account for autocorrelation. However, the interpretation of the results is not as straight forward as linear regression. With regards to formulation, there is two big differences with the one introduced in equation (1) which is the required link function is log function rather than identity function. Additionally, log(person-bed days) needs to be added to the left side of the equation as an offset term.

**Results**

**Site 1**

**Exploratory Analysis 1**

This analysis showed a pre-intervention upward trajectory in adverse events (ß =0.02 CI: 0.00, 0.03), compared to a downward trend in adverse events in the post-intervention period which was significantly different to the pre-intervention trajectory (ß =-0.09, CI: -0.16, -0.01, p=0.03).

| 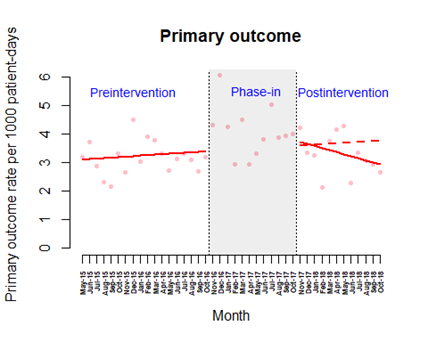 |
| --- |

**Exploratory Analysis 2**

In this analysis, we explored the effect of choosing different cut-points for the pre- and post-intervention periods, across each of the twelve months of the implementation period. This created 13 separate models, with different pre- and post-intervention inflection points. Each of these cut-off points showed a significant downward trajectory for post-intervention trends of adverse events, relative to a slight upward trend in the pre-intervention period.

Table 27: Site 1 exploratory analysis 2

|  |  | Estimate | 95% CI | | P-value |
| --- | --- | --- | --- | --- | --- |
| October 16 | Intercept | 3.06 | 2.72 | 3.41 | <0.00001 |
|  | Preintervention Trend | 0.01 | -0.02 | 0.05 | 0.50 |
|  | Immediate effect of intervention (change in level) | 0.85 | 0.39 | 1.31 | <0.001 |
|  | Change in slope (post-intervention period vs. pre-intervention period) | -0.05 | -0.09 | -0.02 | <0.01 |
| November 16 | Intercept | 2.95 | 2.68 | 3.22 | <0.00001 |
|  | Preintervention Trend | 0.03 | 0.00 | 0.05 | <0.05 |
|  | Immediate effect of intervention (change in level) | 0.76 | 0.38 | 1.15 | <0.001 |
|  | Change in slope (post-intervention period vs. pre-intervention period) | -0.07 | -0.10 | -0.05 | <0.01 |
| December 16 | Intercept | 2.96 | 2.50 | 3.43 | <0.00001 |
|  | Preintervention Trend | 0.02 | -0.02 | 0.06 | 0.27 |
|  | Immediate effect of intervention (change in level) | 0.65 | 0.01 | 1.29 | 0.05 |
|  | Change in slope (post-intervention period vs. pre-intervention period) | -0.07 | -0.12 | -0.02 | 0.01 |
| January 17 | Intercept | 3.07 | 2.94 | 3.20 | <0.00001 |
|  | Preintervention Trend | 0.02 | 0.01 | 0.03 | 0.01 |
|  | Immediate effect of intervention (change in level) | 0.76 | 0.62 | 0.91 | <0.00001 |
|  | Change in slope (post-intervention period vs. pre-intervention period) | -0.07 | -0.09 | -0.06 | <0.00001 |
| February 17 | Intercept | 3.07 | 2.94 | 3.20 | <0.00001 |
|  | Preintervention Trend | 0.02 | 0.01 | 0.03 | <0.00001 |
|  | Immediate effect of intervention (change in level) | 0.75 | 0.61 | 0.91 | 0.01 |
|  | Change in slope (post-intervention period vs. pre-intervention period) | -0.08 | -0.09 | -0.06 | <0.00001 |
| March 17 | Intercept | 2.91 | 2.68 | 3.13 | <0.00001 |
|  | Preintervention Trend | 0.03 | 0.01 | 0.05 | 0.01 |
|  | Immediate effect of intervention (change in level) | 0.57 | 0.15 | 0.98 | <0.00001 |
|  | Change in slope (post-intervention period vs. pre-intervention period) | -0.09 | -0.11 | -0.07 | <0.00001 |
| April 17 | Intercept | 3.00 | 2.88 | 3.12 | <0.00001 |
|  | Preintervention Trend | 0.02 | 0.01 | 0.04 | <0.00001 |
|  | Immediate effect of intervention (change in level) | 0.68 | 0.47 | 0.91 | <0.00001 |
|  | Change in slope (post-intervention period vs. pre-intervention period) | -0.10 | -0.11 | -0.09 | <0.00001 |
| May 17 | Intercept | 2.99 | 2.87 | 3.10 | <0.00001 |
|  | Preintervention Trend | 0.03 | 0.01 | 0.04 | <0.00001 |
|  | Immediate effect of intervention (change in level) | 0.68 | 0.41 | 0.94 | <0.00001 |
|  | Change in slope (post-intervention period vs. pre-intervention period) | -0.11 | -0.13 | -0.06 | <0.00001 |
| June 17 | Intercept | 2.94 | 2.83 | 3.06 | <0.00001 |
|  | Preintervention Trend | 0.03 | 0.02 | 0.04 | <0.00001 |
|  | Immediate effect of intervention (change in level) | 0.58 | 0.26 | 0.90 | <0.00001 |
|  | Change in slope (post-intervention period vs. pre-intervention period) | -0.12 | -0.14 | -0.10 | <0.00001 |
| July 17 | Intercept | 2.87 | 2.70 | 3.03 | <0.00001 |
|  | Preintervention Trend | 0.04 | 0.02 | 0.04 | <0.00001 |
|  | Immediate effect of intervention (change in level) | 0.25 | -0.19 | 0.70 | 0.27 |
|  | Change in slope (post-intervention period vs. pre-intervention period) | -0.12 | -0.15 | -0.08 | <0.00001 |
| August 17 | Intercept | 2.86 | 2.70 | 3.01 | <0.00001 |
|  | Preintervention Trend | 0.04 | 0.03 | 0.05 | 0.01 |
|  | Immediate effect of intervention (change in level) | 0.11 | -0.36 | 0.58 | 0.64 |
|  | Change in slope (post-intervention period vs. pre-intervention period) | -0.11 | -0.16 | -0.07 | <0.00001 |
| September 17 | Intercept | 2.87 | 2.78 | 3.21 | <0.00001 |
|  | Preintervention Trend | 0.04 | 0.03 | 0.04 | 0.01 |
|  | Immediate effect of intervention (change in level) | 0.12 | -0.33 | 0.57 | 0.60 |
|  | Change in slope (post-intervention period vs. pre-intervention period) | -0.13 | -0.18 | -0.09 | <0.00001 |
| October 17 | Intercept | 2.82 | 2.68 | 2.97 | <0.00001 |
|  | Preintervention Trend | 0.04 | 0.03 | 0.05 | <0.00001 |
|  | Immediate effect of intervention (change in level) | -0.31 | -0.80 | 0.19 | 0.23 |
|  | Change in slope (post-intervention period vs. pre-intervention period) | -0.10 | -0.16 | -0.04 | <0.00001 |

**Sensitivity analysis**

In this section, the results of fitting Poisson regression on primary outcome are presented. The results from Poisson regression are fairly similar to those from linear regression. Even though change in slope (post-intervention period vs. implementation period) is in the same direction, it is no longer significant.

Table 28: Site 1 sensitivity analysis

|  | Estimate | 95% CI | | P-value |
| --- | --- | --- | --- | --- |
| Intercept | -5.78 | -6.00 | -5.56 | <0.00001 |
| Trend preintervention | 0.00 | -0.02 | 0.02 | 0.83 |
| Immediate effect after start of intervention (change in level) | 0.18 | -0.16 | 0.52 | 0.30 |
| Change in slope (implementation period vs. pre-intervention period) | 0.00 | -0.04 | 0.04 | 0.98 |
| Immediate effect of intervention (change in level) | -0.05 | -0.38 | 0.28 | 0.78 |
| Change in slope (post-intervention period vs. implementation period) | -0.02 | -0.07 | 0.03 | 0.33 |

**Site 2**

**Exploratory Analysis 1**

No exploratory analysis is possible due to small sample size.

**Exploratory Analysis 2**

For this site, because of small sample size we only could fit seven monthly models. No significant trend was observed.

Table 29: Site 2 exploratory analysis 2

|  |  | Estimate | 95% CI | | P-value |
| --- | --- | --- | --- | --- | --- |
| October 16 | Intercept | 6.73 | 5.18 | 8.28 | 0.00 |
|  | Trend preintervention | -0.09 | -0.35 | 0.16 | 0.49 |
|  | Immediate effect of intervention (change in level) | -0.47 | -2.52 | 1.58 | 0.66 |
|  | Change in trend post-intervention | -0.01 | -0.29 | 0.27 | 0.93 |
| December 16 | Intercept | 5.63 | 1.02 | 10.23 | 0.02 |
|  | Trend preintervention | 0.16 | -0.59 | 0.90 | 0.66 |
|  | Immediate effect of intervention (change in level) | -2.87 | -8.76 | 3.03 | 0.32 |
|  | Change in trend post-intervention | -0.15 | -1.13 | 0.84 | 0.76 |
| February 17 | Intercept | 6.92 | 6.07 | 7.77 | 0.00 |
|  | Trend preintervention | -0.13 | -0.26 | -0.01 | 0.05 |
|  | Immediate effect of intervention (change in level) | -0.04 | -1.30 | 1.23 | 0.95 |
|  | Change in trend post-intervention | 0.00 | -0.17 | 0.16 | 0.98 |
| April 17 | Intercept | 6.91 | 5.66 | 8.16 | 0.00 |
|  | Trend preintervention | -0.13 | -0.30 | 0.05 | 0.16 |
|  | Immediate effect of intervention (change in level) | -0.21 | -2.39 | 1.97 | 0.85 |
|  | Change in trend post-intervention | 0.02 | -0.30 | 0.33 | 0.92 |
| June 17 | Intercept | 6.69 | 5.54 | 7.83 | 0.00 |
|  | Trend preintervention | -0.09 | -0.24 | 0.05 | 0.23 |
|  | Immediate effect of intervention (change in level) | -1.19 | -3.57 | 1.19 | 0.34 |
|  | Change in trend post-intervention | 0.14 | -0.26 | 0.54 | 0.49 |
| August 17 | Intercept | 7.02 | 5.94 | 8.10 | 0.00 |
|  | Trend preintervention | -0.15 | -0.28 | -0.02 | 0.04 |
|  | Immediate effect of intervention (change in level) | 0.06 | -2.71 | 2.82 | 0.97 |
|  | Change in trend post-intervention | 0.02 | -0.54 | 0.58 | 0.96 |
| October 17 | Intercept | 6.95 | 5.93 | 7.98 | 0.00 |
|  | Trend preintervention | -0.14 | -0.25 | -0.02 | 0.03 |
|  | Immediate effect of intervention (change in level) | -0.40 | -3.89 | 3.08 | 0.82 |
|  | Change in trend post-intervention | 0.12 | -0.75 | 0.99 | 0.79 |

**Sensitivity Analysis**

I report the results of fitting zero-inflated Poisson regression on primary outcome using original data, i.e. not two monthly data. Similar to the primary analysis of primary outcome, Zero-inflated Segmented Poisson regression does not show any significant trend change in either of pre-intervention, implementation, or post-intervention phases. Additionally, by every unit increase in month, the log odds of inflated zero increases by 0.37 which is not significant.

Table 30: Site 2 sensitivity analysis

|  | Estimate | 95% CI | | P-value |
| --- | --- | --- | --- | --- |
| Intercept | -5.01 | -5.61 | -4.41 | < 0.00001 |
| Trend preintervention | -0.01 | -0.06 | 0.05 | 0.80 |
| Immediate effect after start of intervention (change in level) | 0.28 | -0.61 | 1.17 | 0.54 |
| Change in trend phase-in | -0.04 | -0.17 | 0.08 | 0.49 |
| Immediate effect of intervention (change in level) | 0.18 | -0.78 | 1.15 | 0.71 |
| Change in trend post-intervention | 0.08 | -0.06 | 0.22 | 0.26 |
| Log odds of inflated zero | 0.37 | -0.01 | 0.75 | 0.05 |

**Site 3**

**Exploratory Analysis 1**

In this analysis, the downward trend in adverse events in the post-intervention period did not reach significance when compared to the pre-intervention trajectory (ß =-0.20, CI: -0.65, 0.25, p=0.37).

| 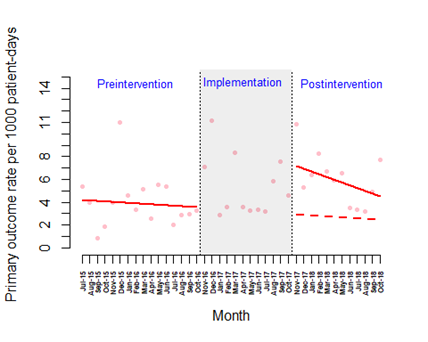 |
| --- |

**Exploratory Analysis 2**

In the second analysis, we explored the effect of choosing different cut-points for the pre- and post-intervention periods, across each of the twelve months of the implementation period. This created 13 separate models, with different pre- and post-intervention inflection points. From June 2017 there was a significant downward trajectory for post-intervention trends of adverse events, relative to a slight upward trend in the pre-intervention period.

Table 31: Site 3 exploratory analysis 2

|  |  | Estimate | 95% CI | | P-value |
| --- | --- | --- | --- | --- | --- |
| October 16 | Intercept | 3.00 | 1.71 | 4.28 | <0.00001 |
|  | Trend preintervention | 0.05 | -0.07 | 0.18 | 0.41 |
|  | Immediate effect of intervention (change in level) | 0.25 | -1.21 | 1.72 | 0.74 |
|  | Change in trend post-intervention | 0.01 | -0.12 | 0.14 | 0.88 |
| November 16 | Intercept | 3.06 | 1.90 | 4.23 | <0.00001 |
|  | Trend preintervention | 0.04 | -0.06 | 0.15 | 0.43 |
|  | Immediate effect of intervention (change in level) | 0.48 | -0.90 | 1.85 | 0.50 |
|  | Change in trend post-intervention | 0.01 | -0.11 | 0.13 | 0.86 |
| December 16 | Intercept | 2.90 | 1.81 | 3.99 | <0.00001 |
|  | Trend preintervention | 0.06 | -0.03 | 0.16 | 0.21 |
|  | Immediate effect of intervention (change in level) | 0.17 | -1.17 | 1.51 | 0.80 |
|  | Change in trend post-intervention | 0.00 | -0.11 | 0.11 | 0.98 |
| January 17 | Intercept | 2.90 | 1.81 | 3.99 | <0.00001 |
|  | Trend preintervention | 0.06 | -0.03 | 0.16 | 0.21 |
|  | Immediate effect of intervention (change in level) | 0.17 | -1.17 | 1.51 | 0.80 |
|  | Change in trend post-intervention | 0.00 | -0.11 | 0.11 | 0.98 |
| February 17 | Intercept | 3.04 | 2.06 | 4.01 | <0.00001 |
|  | Trend preintervention | 0.04 | -0.03 | 0.12 | 0.28 |
|  | Immediate effect of intervention (change in level) | 0.79 | -0.52 | 2.09 | 0.24 |
|  | Change in trend post-intervention | -0.01 | -0.11 | 0.09 | 0.84 |
| March 17 | Intercept | 2.95 | 2.02 | 3.88 | <0.00001 |
|  | Trend preintervention | 0.05 | -0.02 | 0.12 | 0.14 |
|  | Immediate effect of intervention (change in level) | 0.64 | -0.65 | 1.93 | 0.34 |
|  | Change in trend post-intervention | -0.02 | -0.12 | 0.08 | 0.68 |
| April 17 | Intercept | 2.96 | 2.07 | 3.84 | <0.00001 |
|  | Trend preintervention | 0.05 | -0.01 | 0.12 | 0.10 |
|  | Immediate effect of intervention (change in level) | 0.78 | -0.48 | 2.05 | 0.23 |
|  | Change in trend post-intervention | -0.04 | -0.14 | 0.07 | 0.50 |
| May 17 | Intercept | 3.01 | 2.17 | 3.85 | <0.00001 |
|  | Trend preintervention | 0.05 | -0.01 | 0.11 | 0.09 |
|  | Immediate effect of intervention (change in level) | 1.07 | -0.15 | 2.30 | 0.09 |
|  | Change in trend post-intervention | -0.06 | -0.16 | 0.04 | 0.28 |
| June 17 | Intercept | 3.09 | 2.29 | 3.89 | <0.00001 |
|  | Trend preintervention | 0.05 | 0.00 | 0.10 | 0.07 |
|  | Immediate effect of intervention (change in level) | 1.38 | 0.16 | 2.61 | 0.03 |
|  | Change in trend post-intervention | -0.09 | -0.20 | 0.02 | 0.11 |
| July 17 | Intercept | 3.10 | 2.31 | 3.88 | <0.00001 |
|  | Trend preintervention | 0.05 | 0.00 | 0.10 | 0.04 |
|  | Immediate effect of intervention (change in level) | 1.49 | 0.22 | 2.76 | 0.03 |
|  | Change in trend post-intervention | -0.12 | -0.24 | -0.01 | 0.04 |
| August 17 | Intercept | 3.17 | 2.43 | 3.92 | <0.00001 |
|  | Trend preintervention | 0.05 | 0.01 | 0.09 | 0.02 |
|  | Immediate effect of intervention (change in level) | 1.80 | 0.50 | 3.10 | 0.01 |
|  | Change in trend post-intervention | -0.18 | -0.30 | -0.05 | 0.01 |
| September 17 | Intercept | 3.10 | 2.38 | 3.83 | <0.00001 |
|  | Trend preintervention | 0.06 | 0.02 | 0.10 | 0.01 |
|  | Immediate effect of intervention (change in level) | 1.73 | 0.36 | 3.10 | 0.02 |
|  | Change in trend post-intervention | -0.21 | -0.36 | -0.06 | 0.01 |
| October 17 | Intercept | 3.12 | 2.41 | 3.82 | <0.00001 |
|  | Trend preintervention | 0.06 | 0.02 | 0.10 | <0.00001 |
|  | Immediate effect of intervention (change in level) | 1.92 | 0.48 | 3.36 | 0.01 |
|  | Change in trend post-intervention | -0.27 | -0.45 | -0.10 | <0.00001 |

**Sensitivity analysis**

Apart from the preintervention level and level change in post-intervention, the results from Poisson regression are fairly similar to those from linear regression. Even though change in slope (post-intervention period vs. implementation period) is in the same direction, it is no longer significant .

Table 32: Site 3 sensitivity analysis

|  | Estimate | 95% CI | | P-value |
| --- | --- | --- | --- | --- |
| Intercept | -5.59 | -5.99 | -5.20 | <0.00001 |
| Trend preintervention | 0.00 | -0.04 | 0.04 | 0.93 |
| Immediate effect after start of intervention (change in level) | 0.29 | -0.27 | 0.84 | 0.31 |
| Change in trend phase-in | 0.00 | -0.07 | 0.07 | 0.99 |
| Immediate effect of intervention (change in level) | 0.22 | -0.40 | 0.84 | 0.49 |
| Change in trend post-intervention | -0.01 | -0.08 | 0.06 | 0.69 |

**Site 4**

**Exploratory Analyses**

This analysis showed a slight downward trajectory in adverse events during the pre-intervention period that was not significant (ß =-0.14 CI: -1.12, 0.85; p=0.78). In the post-intervention period, there was a slight downward change in the post-intervention slope compared with the trajectory but this was not significant (ß =-0.10, CI: -1.90, 1.71; p=0.91)

| 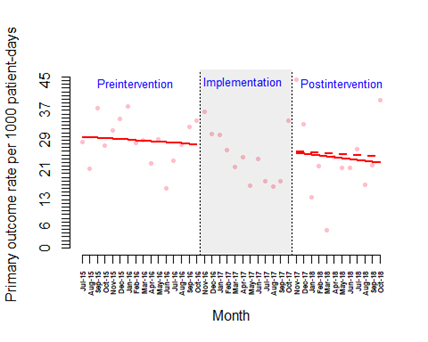 |
| --- |

**Exploratory Analysis 2**

There was a small but non-significant change in post-intervention trend in all the models.

Table 33: Site 4 exploratory analysis 2

|  |  | Estimate | 95% CI | | P-value |
| --- | --- | --- | --- | --- | --- |
| October 16 | Intercept | 28.42 | 25.62 | 31.22 | <0.00001 |
|  | Trend preintervention | 0.07 | -0.22 | 0.36 | 0.64 |
|  | Immediate effect of intervention (change in level) | -3.27 | -6.36 | -0.19 | 0.04 |
|  | Change in trend post-intervention | -0.26 | -0.54 | 0.01 | 0.07 |
| November 16 | Intercept | 29.21 | 26.92 | 31.50 | <0.00001 |
|  | Trend preintervention | -0.03 | -0.25 | 0.18 | 0.77 |
|  | Immediate effect of intervention (change in level) | -3.20 | -6.07 | -0.33 | 0.04 |
|  | Change in trend post-intervention | -0.13 | -0.34 | 0.08 | 0.24 |
| December 16 | Intercept | 29.47 | 27.37 | 31.57 | <0.00001 |
|  | Trend preintervention | -0.06 | -0.25 | 0.12 | 0.50 |
|  | Immediate effect of intervention (change in level) | -3.30 | -6.11 | -0.50 | 0.03 |
|  | Change in trend post-intervention | -0.07 | -0.27 | 0.13 | 0.49 |
| January 17 | Intercept | 29.47 | 27.37 | 31.57 | <0.00001 |
|  | Trend preintervention | -0.06 | -0.25 | 0.12 | 0.50 |
|  | Immediate effect of intervention (change in level) | -3.37 | -6.21 | -0.53 | 0.03 |
|  | Change in trend post-intervention | -0.07 | -0.27 | 0.13 | 0.49 |
| February 17 | Intercept | 29.47 | 27.37 | 31.57 | <0.00001 |
|  | Trend preintervention | -0.06 | -0.25 | 0.12 | 0.50 |
|  | Immediate effect of intervention (change in level) | -3.37 | -6.21 | -0.53 | 0.03 |
|  | Change in trend post-intervention | -0.07 | -0.27 | 0.13 | 0.49 |
| March 17 | Intercept | 29.51 | 27.40 | 31.62 | <0.00001 |
|  | Trend preintervention | -0.07 | -0.26 | 0.12 | 0.48 |
|  | Immediate effect of intervention (change in level) | -3.42 | -6.31 | -0.53 | 0.03 |
|  | Change in trend post-intervention | -0.07 | -0.27 | 0.13 | 0.52 |
| April 17 | Intercept | 29.53 | 27.40 | 31.66 | <0.00001 |
|  | Trend preintervention | -0.07 | -0.26 | 0.12 | 0.48 |
|  | Immediate effect of intervention (change in level) | -3.47 | -6.42 | -0.51 | 0.03 |
|  | Change in trend post-intervention | -0.06 | -0.27 | 0.14 | 0.54 |
| May 17 | Intercept | 29.57 | 27.41 | 31.72 | <0.00001 |
|  | Trend preintervention | -0.07 | -0.26 | 0.12 | 0.47 |
|  | Immediate effect of intervention (change in level) | -3.49 | -6.52 | -0.46 | 0.03 |
|  | Change in trend post-intervention | -0.06 | -0.28 | 0.16 | 0.58 |
| June 17 | Intercept | 29.61 | 27.43 | 31.79 | <0.00001 |
|  | Trend preintervention | -0.08 | -0.27 | 0.12 | 0.45 |
|  | Immediate effect of intervention (change in level) | -3.50 | -6.62 | -0.38 | 0.03 |
|  | Change in trend post-intervention | -0.06 | -0.29 | 0.18 | 0.63 |
| July 17 | Intercept | 29.65 | 27.44 | 31.85 | <0.00001 |
|  | Trend preintervention | -0.08 | -0.28 | 0.12 | 0.44 |
|  | Immediate effect of intervention (change in level) | -3.50 | -6.70 | -0.30 | 0.04 |
|  | Change in trend post-intervention | -0.06 | -0.31 | 0.20 | 0.67 |
| August 17 | Intercept | 29.71 | 27.48 | 31.93 | <0.00001 |
|  | Trend preintervention | -0.08 | -0.29 | 0.12 | 0.42 |
|  | Immediate effect of intervention (change in level) | -3.47 | -6.76 | -0.18 | 0.05 |
|  | Change in trend post-intervention | -0.05 | -0.33 | 0.23 | 0.73 |
| September 17 | Intercept | 29.77 | 27.53 | 32.01 | <0.00001 |
|  | Trend preintervention | -0.09 | -0.29 | 0.11 | 0.39 |
|  | Immediate effect of intervention (change in level) | -3.42 | -6.79 | -0.05 | 0.05 |
|  | Change in trend post-intervention | -0.04 | -0.35 | 0.27 | 0.79 |
| October 17 | Intercept | 31.29 | 29.71 | 32.87 | <0.00001 |
|  | Trend preintervention | -0.25 | -0.35 | -0.15 | <0.00001 |
|  | Immediate effect of intervention (change in level) | -2.61 | -7.90 | 2.68 | 0.34 |
|  | Change in trend post-intervention | 0.48 | -0.22 | 1.19 | 0.19 |

Sensitivity analysis

Similar to the segmented linear regression, segmented Poisson regression did not showed any significant trend in Swansea dataset.

Table 34: Site 4 sensitivity analysis

|  | Estimate | 95% CI | | P-value |
| --- | --- | --- | --- | --- |
| Intercept | -3.50 | -3.72 | -3.28 | <0.00001 |
| Trend preintervention | 0.00 | -0.03 | 0.02 | 0.70 |
| Immediate effect after start of intervention (change in level) | 0.14 | -0.21 | 0.50 | 0.43 |
| Change in trend phase-in | -0.04 | -0.09 | 0.01 | 0.11 |
| Immediate effect of intervention (change in level) | 0.35 | -0.09 | 0.79 | 0.12 |
| Change in trend post-intervention | 0.03 | -0.03 | 0.09 | 0.39 |
