# Supplementary material for: Development, implementation and evaluation of an evidence-based paediatric early warning system improvement programme: the PUMA mixed methods study
Source: BMC Health Serv Res. 2022 Jan 2;22:9. doi: 10.1186/s12913-021-07314-2 (PMC8722056; doi:10.1186/s12913-021-07314-2)
Supplement: Supplementary file 4 — Additional file 4. [file 12913_2021_7314_MOESM4_ESM.pdf]

Implementation **Guide**

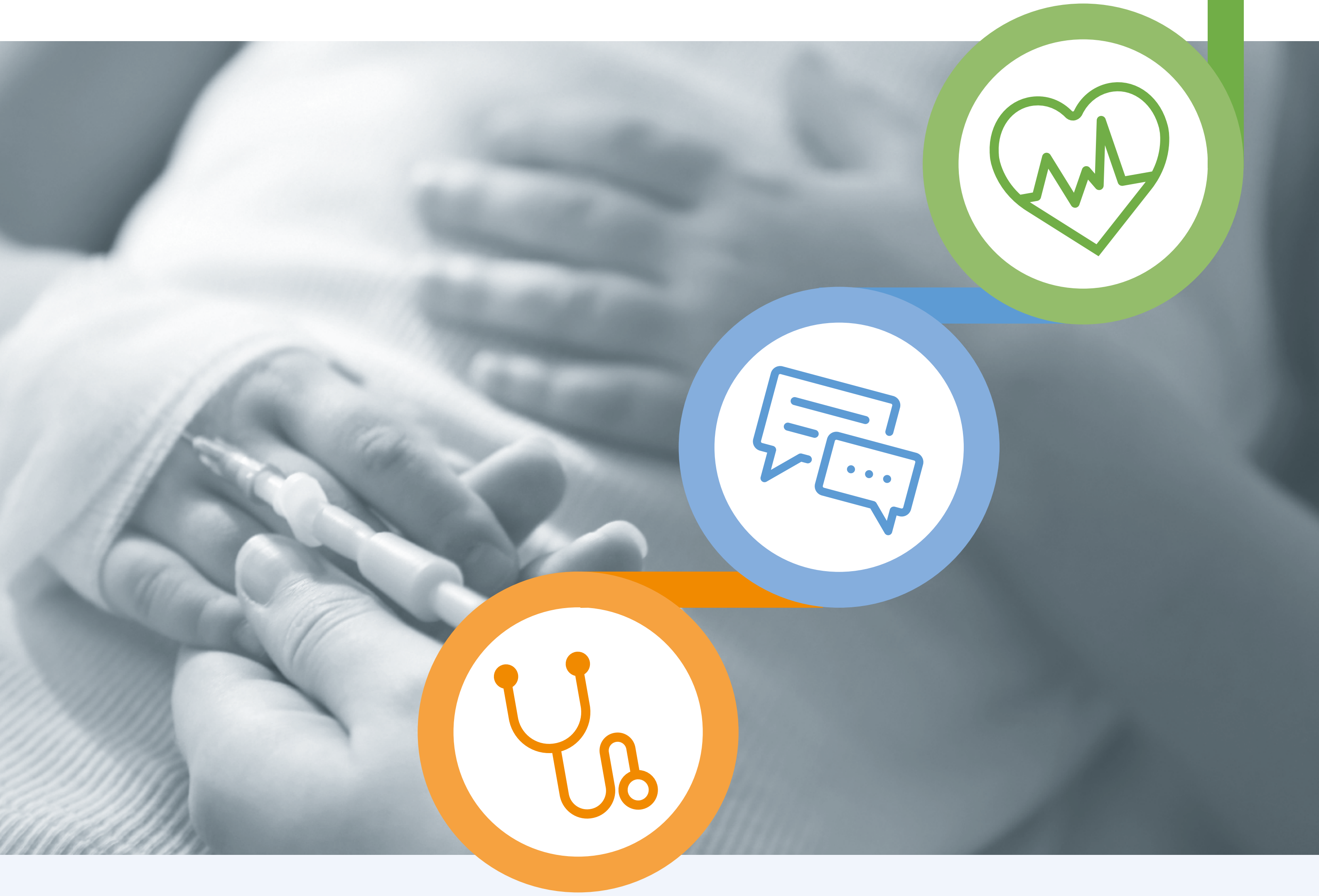

PUMA Programme

An Evidence Based Paediatric Early Warning System Improvement Programme

PUMA

The PUMA Programme is a product of the PUMA study, which was commissioned by the National Institute for Health Research. The PUMA study was led by the School of Healthcare Sciences and the Centre for Trials Research at Cardiff University in collaboration with the University of Leicester, the University of Liverpool and the University of Salford.

Cardiff University / Prifysgol Caerdydd — Centre for Trials Research / Canolfan Ymchwil Treialon

Cardiff University / Prifysgol Caerdydd — School of Healthcare Sciences / Ysgol y Gwyddorau Gofal Iechyd

NHS National Institute for Health Research

University of Liverpool

University of Leicester

University of Salford Manchester

To use or evaluate any of the content of this guide you will need to apply for a license from Cardiff University.

Contents

Who should use this Guide **4**
How to use this Guide **5**

The Puma Programme **6**
Introduction 7
PUMA Wheel 8
PUMA approach to embedding change in practice 10
What is the evidence for the PUMA Programme? 11

Implementing the PUMA Programme **13**
Step 1 Form your Improvement Team 14
Step 2 Assess your system 16
Step 3 Plan your improvement initiatives 20
Step 4 Implement and review initiatives 28
Step 5 Sustain progress 32

Appendices **33**
Appendix 1 The core components of a Paediatric Early Warning System: The PUMA Standard 34
Appendix 2 System assessment tool 38
Appendix 3 Family feedback form 53
Appendix 4 What others have done 56

Worksheets **62**
Worksheet 1 Your essential Improvement Team members 63
Worksheet 2 System assessment checklist 64
Worksheet 3 Summary assessment and radar 65
Worksheet 4 Action planning 68
Worksheet 5 Implementing your initiatives 69
Worksheet 6 Reviewing your initiatives 70

Who should use this Guide

This Guide should be used by healthcare improving and clinical managers who are interested in improving their paediatric early warning system.

It can be used to implement change at different levels, from a single ward to a whole organisation.

How to use this Guide

This Guide describes the PUMA Programme and outlines the steps involved in implementation.

Each participating clinical area should be provided with at least one copy of the Guide.

There are optional worksheets to help you to complete each step; these can be found on pages 62-71. Print and complete multiple copies if required.

Tips are indicated by this symbol and will help you to avoid some common pitfalls in implementing change.

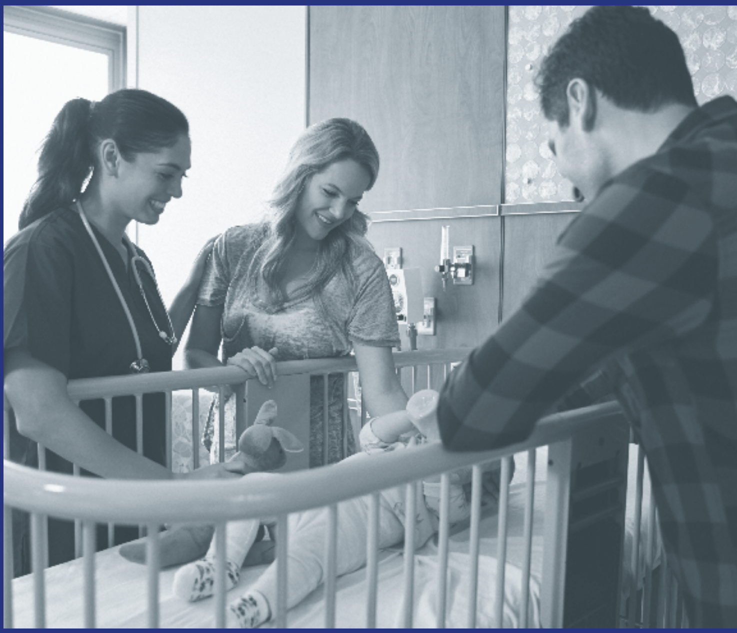

The PUMA Programme

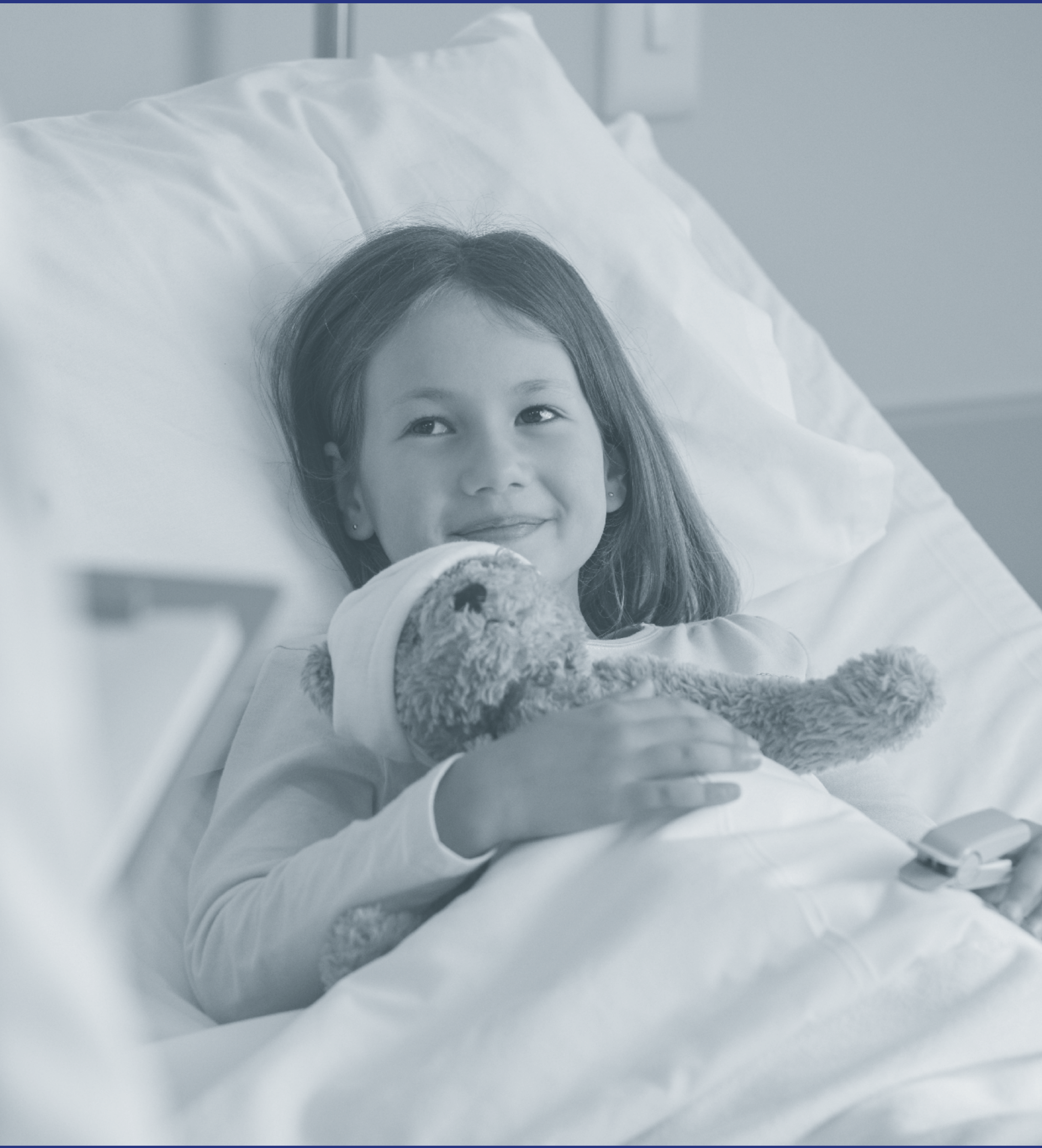

In 2011 a research study compared the child health outcomes and death rate in the UK with other European countries. It was worrying that UK measures of child health were amongst the worst in Europe. The PUMA Programme is an evidence-based approach to improving paediatric patient safety in hospital.

The aim of the Programme is to improve your system for detecting and responding to deterioration by helping you to:

- Assess your system and identify opportunities for improvement
- Select and implement initiatives that will function in your setting
- Embed initiatives in routine practice
- Review initiatives
- Sustain progress

The PUMA Programme has two key elements:

1. An evidence-based model of how an effective system to detect and respond to deterioration should function (The PUMA Standard)
2. An evidence-based approach to embedding and sustaining change in practice

PUMA Wheel

The PUMA Wheel (Figure 1) is a visual summary of the PUMA Standard which can be found in Appendix 1.

There are three key parts to the system: detect, plan and act.

To effectively **DETECT** signs of deterioration you need to accurately monitor, record and interpret signs of deterioration.	This evidence needs to be collated and shared to enable the team to **PLAN** to act, using available skills and resources.	**ACT** in response to deterioration and evaluate.

Throughout this document, we will use these three colours to highlight where activities fit within the PUMA Wheel.

Figure 1 PUMA Wheel

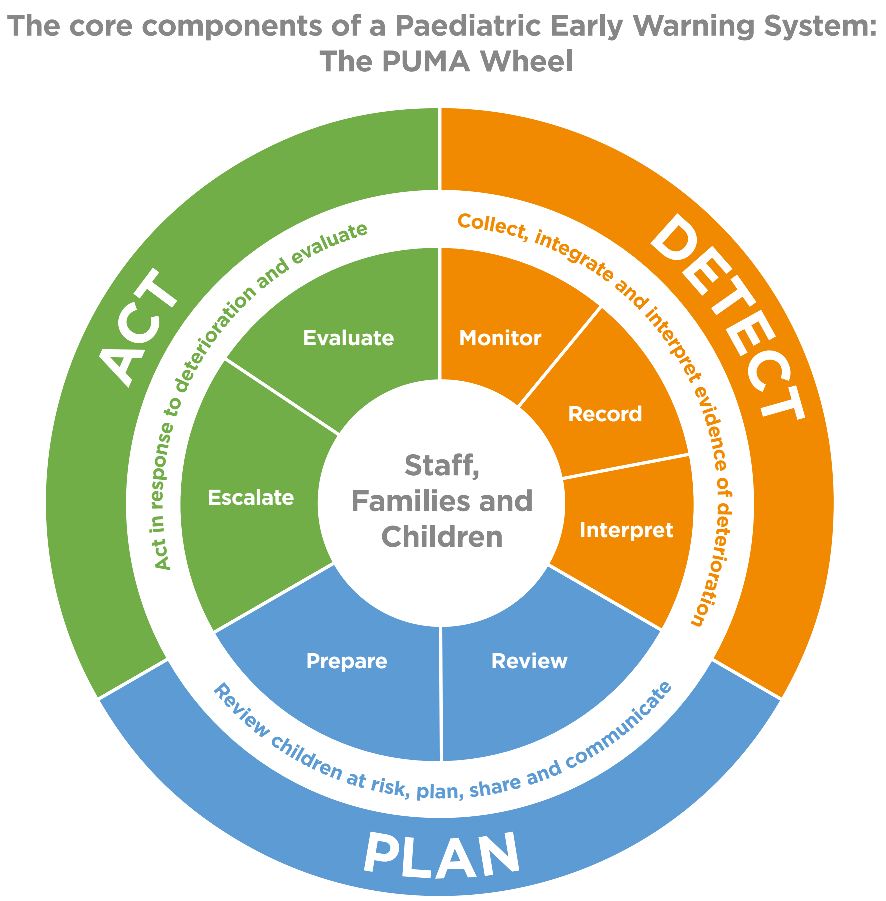

**DETECT**<br>*Collect, integrate and interpret evidence of deterioration*	**PLAN**<br>*Review children at risk, plan, share and communicate*	**ACT**<br>*Act in response to deterioration and evaluate*
**Monitor**<br>Mechanisms to ensure key vital signs (core indicators appropriate for all children and additional indicators for patients specific groups) are monitored reliably with appropriate frequency and accuracy.	**Review**<br>Mechanisms to ensure indicators of deterioration are reviewed to identify children who are a concern.	**Escalate**<br>Mechanisms to ensure clear escalation and response processes.
**Record**<br>Mechanisms to ensure key vital signs and other indicators of deterioration are recorded reliably with appropriate frequency and accuracy.	**Prepare**<br>Mechanisms to ensure staff are aware at ward level of the status of in individual patients and the availability of resources to plan an appropriate response.	**Evaluate**<br>Mechanisms to ensure escalation and response processes are evaluated.
**Interpret**<br>Mechanisms to ensure key vital signs and other indicators of deterioration are interpreted reliably with appropriate frequency and accuracy.		

PUMA Programme approach to embedding change in practice

The PUMA Programme starts with some basic, evidence-based, assumptions about how to embed change in practice:

1 Problems should be defined before solutions are selected

Before determining whether or not a solution is the right one, it is important to to accurately define current operations, constraints and goals.

2 Systems are complex

How a system performs might be affected by a multitude of factors, differing across wards and organisations.

3 Interventions should be adapted to context

Context affects the efficacy of an intervention: what works in one setting may not work in others.

4 Improvement requires local expertise

Top down approaches that do not enable teams to adapt to their own clinical contexts can be problematic. New processes need to be integrated into existing processes by people who understand the context.

5 The function of an intervention is more important than it's form

Improvement efforts need to focus on the active ingredients of an intervention – what it does, rather than what it looks like.

6 Improving systems is a continuous process

Processes for reviewing, assessing and improving your system need to be embedded in routine practice. It's important to recognise that there are often multiple ways of achieving the same goal, so if one intervention doesn't work you should try another.

What is the evidence for the PUMA Programme?

Five key sources of evidence have been used to develop the PUMA Programme. These can be seen below in Figure 2:

1. Three systematic reviews on Paediatric Track and Trigger Tools (PTTTs) and systems:

Q1: How well validated are existing PTTTs and their component parts?

Q2: How effective are PTTTs and wider systems at reducing mortality and critical events?

Q3: What are the contextual factors associated with successful/unsuccessful systems (with or without PTTT)?

2. An emerging body of evidence on implementation and improvement in healthcare

3. Social theories

4. Clinical expertise

5. Patient and Public Involvement

Figure 2 Developing the PUMA Programme

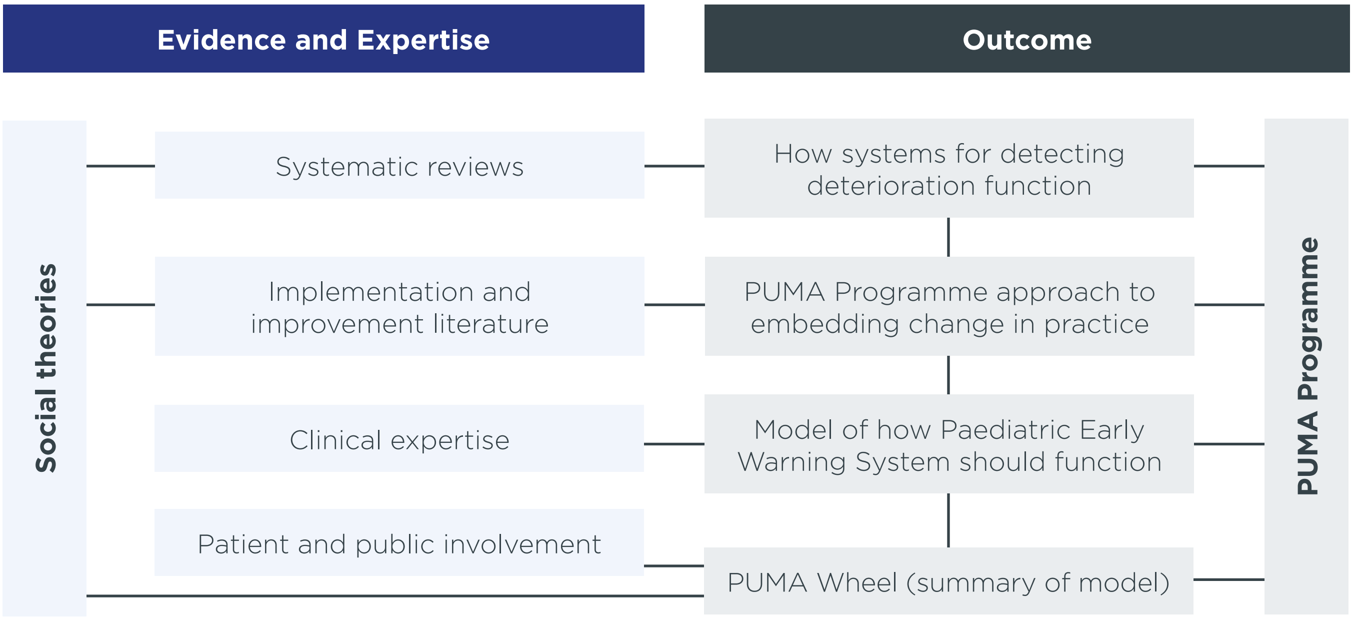

The systematic reviews showed that there are a great number of different Paediatric Track and Trigger Tools (PTTT) currently being used in paediatric units, but only a handful of these had been validated in a research setting.

There were few effectiveness papers on validated tools (some tools were never implemented in practice) and all had some methodological limitations. Most effectiveness papers reported simultaneous introduction of system changes (e.g. calling criteria and dedicated response team), making it difficult to delineate the impact of the PTTT from the impact of other system changes.

The evidence from the systematic reviews pointed to multiple failure points in the system, which were focused particularly around three key elements:

Collect, integrate and interpret evidence of deterioration **(detect)**

Review children at risk, plan, share and communicate **(plan)**

Act in response to deterioration and evaluate **(act)**

These three elements have become the three components of the PUMA Wheel. The majority of suggested solutions in the literature were context specific (i.e. may not work in all settings).

Learning from practice

The PUMA Programme was developed by academics, clinicians and patients and implemented and evaluated in four hospitals across the UK, and subsequently implemented in a further three sites, as part of a research study.

Implementing the PUMA Programme in seven different hospitals helped us to gain a better understanding about how important it is to standardise the process of assessing and improving systems, rather than standardising the interventions. Each system has a different "fingerprint"; there is variation in the way each system functions, leading to necessary differences in the selection of solutions. Throughout the Implementation Guide you will find reference to the experience of the seven sites.

Implementing the PUMA Programme

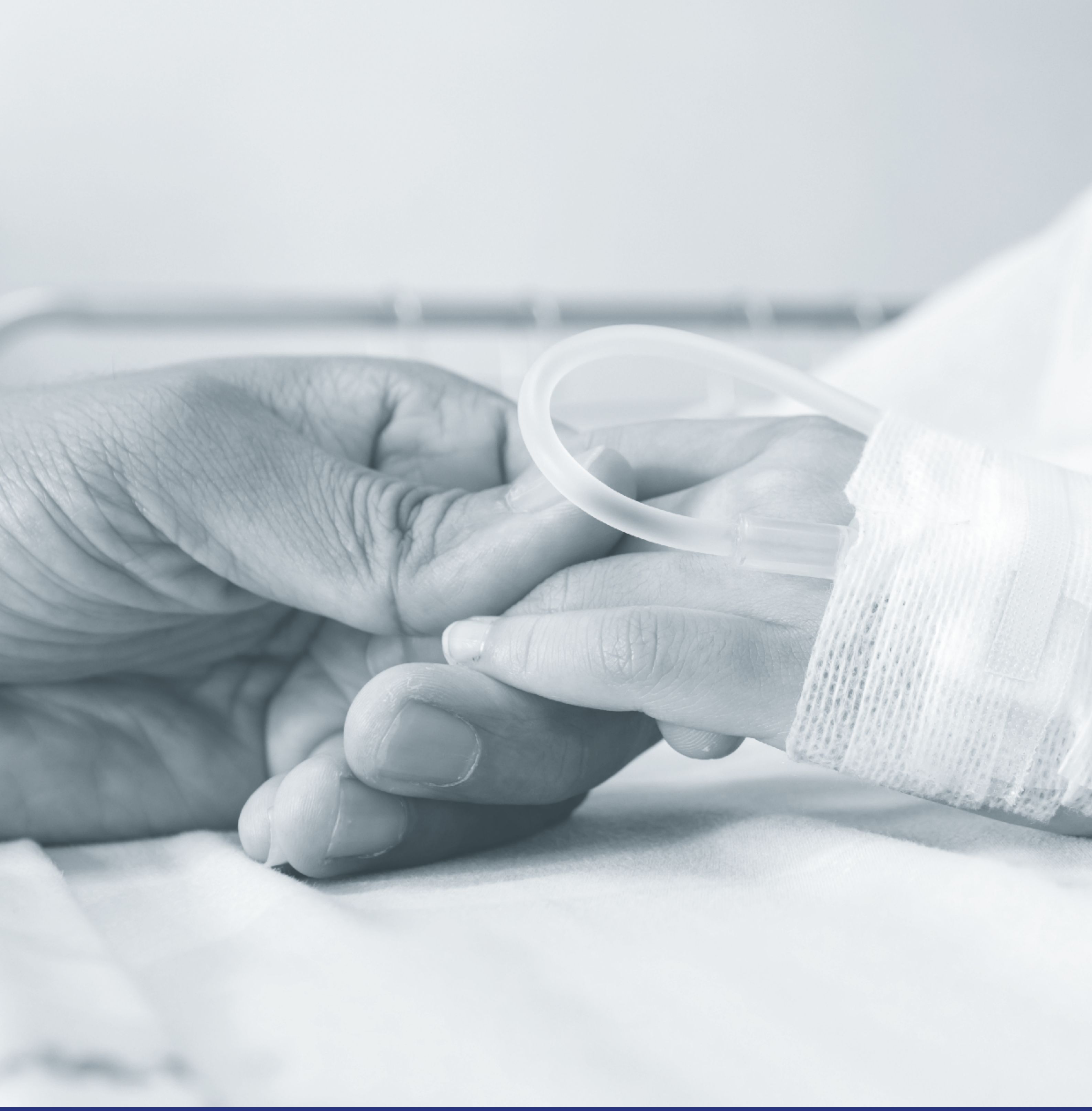

Step 1 Form your Improvement Team

Implementing any new tool or activity requires the support of a team; including the right people on the Improvement Team is crucial to success.

Your Improvement Team can vary in size and composition but at a minimum needs to include leaders and staff members with the authority, expertise, credibility and motivation necessary to drive a successful initiative.

If you are implementing change across your organisation (i.e. not on a single ward), you will first need to form an organisational-level Improvement Team and select wards to implement the PUMA Programme. These wards will then need to follow a similar process, with each then forming their own ward-level Improvement Teams.

Essential Improvement Team members

A description of the key characteristics and primary role of essential Improvement Team members can be found in Table 1. At a minimum, your team needs to include one person representing each of the essential Improvement Team member types. The worksheet on page 63 can be used to record and describe who will be taking on roles and is a useful tool for ensuring you have the right people on your team.

Table 1 Essential Improvement Team members: key characteristics and primary roles

Improvement Team Member	Key Characteristics	Primary Role(s)
**Organisational Sponsor**	• Enough "clout" in the organisation to implement new approaches to care.<br>• Authority to allocate time and resources necessary to achieve team's aim(s)<br>• Authority over all areas that will be affected by the change	• Sponsors and visibly supports Improvement Team<br>• Creates the vision of the new system for the organisation as a whole<br>• Leads the spread of specific changes throughout the organisation or system
**Clinical or Technical Experts (Champions)**	• Expert knowledge of the relevant clinical subject matter<br>• Understands the processes of care within workspace where changes will occur<br>• Good working relationship with colleagues and front-line leaders<br>• Interest in driving/leading change	• Responsible for coaching and role-modelling the team behaviours and skills<br>• Responsible for keeping the organisational sponsor updated
**Implementation Lead**	• Understands details of the organisation (unit/department)<br>• Understands effects of making changes in the organisation<br>• Able to work effectively with doctor/nurse champions	• Is the critical driving force on the team<br>• Assures that changes are tested/measured<br>• Provides oversight for data collection

Adapted from Institute for Healthcare Improvement, http://www.ihi.org/resources/Pages/HowtoImprove/ScienceofImprovementFormingtheTeam.aspx

For the clinical or technical experts, it is essential to have at least one Doctor and one Nurse.

I chose the team because I knew what skills they had and I knew they were dynamic... I knew that they would be hands on with regards to getting people to change where perhaps I couldn't get the change.

Implementation Lead in a District General Hospital

Learning from practice

The sites that have implemented the PUMA Programme to date have formed their Improvement Teams by:

- Drawing on people around who showed an interest
- Presenting at local meetings and asking for volunteers
- Involving people who want to make a difference and have relevant skills and experience

Step 2 Assess your system

You need to have formed your Improvement Team before assessing your system.

Conducting a System Assessment will help you to identify problems/opportunities for improvement in your system.

The System Assessment measures:

- Staff views (using the Staff System Assessment Tool)

- Families' views (using the Family Feedback Tool)

The System Assessment Tool helps you and your team assess each part of your system:

DETECT
Collecting, integrating and interpreting evidence of deterioration

PLAN
Review children at risk, plan, share and communicate

ACT
Escalate action in response to evidence of deterioration and evaluate the process

Completing this process will help you to evaluate your current system and identify areas for improvement.

If you are implementing change across your organisation you will need to complete ward level system assessments for each of your selected wards first. You can then use these ward level system assessments to identify frequent or shared organisational level problems.

Process for completing the system assessment

There are two tools to help you complete your System Assessment: the System Assessment Tool (Appendix 2) and the Family Feedback Tool (Appendix 3). There is a two-stage process for completing the System Assessment on your ward:

Stage 1 Individual assessments

a. One or more ward-based representatives from each of the mandatory staff groups (listed below) should complete the System Assessment Tool individually, and then return to a designated member of staff. Where relevant, staff from other groups working on the ward should also be included in this process.

Mandatory staff groups:	Plus other relevant staff, which may include:
**A** Healthcare Assistants	**H** Nursing bank/agency/locum staff
**B** Staff nurses	**I** Play specialists
**C** Senior staff nurses	**J** Relevant Allied Health Professionals
**D** Sisters / Ward managers	**K** Assistant practitioners
**E** Consultants	**L** Specialty consultants (e.g. respiratory, neurology etc)
**F** ST4-ST8 paediatric trainees or clinicians/ACPs working on a middle grade rota	**M** Consultant paediatric surgeons
**G** ST1-ST3 paediatric trainees or clinicians/ACPs working on a junior grade rota (may also be known as SHOs)	**N** Associate specialists/staff paediatrician (may also be known as trust grades)
	**O** Nursing students
	**P** Medical students

Note: Where ST3 grade clinicians are used on a middle grade rota, these staff should be counted as Category F

b. Family members of all children who are patients the ward on a given day complete the Family Feedback Tool. Provision should be made for families to fill in and return the completed questionnaires anonymously (e.g. into a designated, confidential "family feedback reply" box located at reception or another area easily accessible to family members).

Stage 2 Summary assessment

Your PUMA Improvement Team (ward level) meet to review all of the completed assessment forms, and to discuss differences between individual staff assessments and between staff and family assessments.

Don't skip this stage. Set aside some time for a dedicated meeting. Getting your full Improvement Team involved in assessing your system will increase your likelihood of successful implementation.

Don't worry if staff do not agree in their assessments; disagreements are an opportunity for you to better understand the issues that have arisen from a number of different perspectives.

Based on these discussions, you will need to populate the PUMA Summary Assessment & Radar Tool on pages 65-67 for your ward. The tool requires you to consider each of the seven elements of the PUMA Wheel in turn, summarising the ward's strengths and weaknesses in each area based on feedback from the individual assessments.

Finally, you will be asked to rate each of the seven elements of the PUMA Wheel on a 0-10 scale, based on a weighing up of the various strengths and weaknesses identified.

- A high score here would represent an area in which the individual assessments point to your ward having a number of robust, standardised processes.

- A low score would represent an area in which individual assessments point to your ward having few or no standardised processes.

Once the scores are entered for each area, the 'radar graph' (see page 67) will give a visual summary of how well the ward is currently performing in the different areas of the PUMA Wheel.

Use the System Assessment Checklist on page 64 to help you follow this process.

Learning from practice

Sites that have implemented the PUMA Programme found that they needed to set aside time to complete the system assessment and review the results.

They found that completing the system assessment made the process of improvement easier by:

- Engaging each of the staff groups in the process of improvement from an early stage
- Providing evidence of good practice
- Providing evidence of areas for improvement

Completing the system assessment was challenging, but definitely beneficial. It supported change throughout the process and enabled us to say "you said, we did". It also gave us positive feedback about some of the processes we already had in place.

Implementation Lead in a Tertiary centre

It wasn't just us plucking out what we wanted to take forward, this is what everybody on the team said needs improving.

Implementation Lead in a District General Hospital

Step 3 Plan your improvement initiatives

You need to have completed your System Assessment before planning your improvement initiatives.

Attending a PUMA Action Planning Session will help your Improvement Team to systematically plan your improvement initiatives to:

- Prioritise your problems/opportunities for improvement
- Define your goals and select your initiatives
- Plan your initiatives
- Set your timeline for implementation

If you are implementing the PUMA programme across your organisation, your organisational level Improvement Team will need to meet with your ward level Improvement Teams to discuss and agree initiatives. Ward and organisational level teams should complete separate, complementary, action plans.

The following sections will help you to prepare for the PUMA Action Planning Session. During the PUMA Action Planning Session you will be asked to complete Worksheet 4: Action Planning Template (Page 68).

Prioritise your problems/opportunities for improvement

Your system assessment may have highlighted multiple areas for improvement. Understanding how these problems occur (who is involved, and when and where they occur will help you to understand the frequency and potential harm of the problem, as well as the potential for change.

Focus your efforts on problems that occur frequently, could result in harm and for which a change is feasible.

Define your goals and select your initiative.

Successful improvement requires the identification of clear goals. Agreeing your improvement goals will help you allocate the right people and resources, and measure success.

When selecting your improvement initiative(s) you need to consider how it will help you to reach your goal. You will also need consider whether each initiative matches the following criteria:

- **Targets a specific, defined area for improvement identified during your system assessment**
- **Consistent with current processes and system operating procedures**

- **Parent, family and child focused**
- **Acceptable to staff and families**
- **Sustainable**
- **Can be integrated into normal routine practice**

Measurable

- Desired result can be measured
- Data is accessible, complete, and accurate
- Effectiveness can be monitored over time for continuous improvements

Feasible in terms of:

- Costs
- Staffing and staff skills
- Equipment and other resources available

You will also need to consider:

- Leadership
- Staff turnover
- Training and induction

For example, an intervention is more likely to be successful if it:

- Simplifies procedures and protocols
- Standardises equipment, procedures, protocols
- Minimises reliance on memory
- Clarifies responsibilities and details task descriptions
- Ensures a suitably qualified person performs each task
- Improves communication and information transfer between staff and between patients and staff
- Avoids significantly increasing workloads

Adapted from the Agency for Healthcare Research and Quality, https://www.ahrq.gov/teamstepps/instructor/essentials/implguide2.html

Implementation efforts are often thwarted by attempts to integrate an intervention that doesn't 'fit' the local setting (even though it worked elsewhere). Thinking about exactly how a proposed intervention is going to help address your problem in practice will help you consider whether it will be feasible, and if it is not, will help you identify alternative methods for addressing the problem within your local context.

See Appendix 4 for practical examples of what others have done, but remember that there may be other ways of improving your system that are better suited to your local setting. Most teams do not select off-the-shelf interventions as these have not been tailored to their local requirements, and improvements can often be achieved based on many small changes, rather than radical changes.

Learning from practice

Improvement Teams from different hospitals came together for the Action Planning Sessions.

By the end of the sessions, each site had completed an Action Plan, detailing the overall results of their System Assessment, the key issues identified, and the key initiatives proposed to address the lowest scoring areas (Figure 3).

Understand how your initiative is going to address the area for improvement

It is important that the area of improvement is underpinned by more detail:

- Where does the problem occur?
- How does it occur?
- Why does it occur?

There may be more than one solution for this issue. You will need to carefully think through:

- How the proposed solution might work
- How you will know whether it has worked (i.e. What can you measure before you start implementing a change that will help you to assess whether that change has had an effect?)
- How you are going to implement and embed this change

Example driver diagram for Action Planning

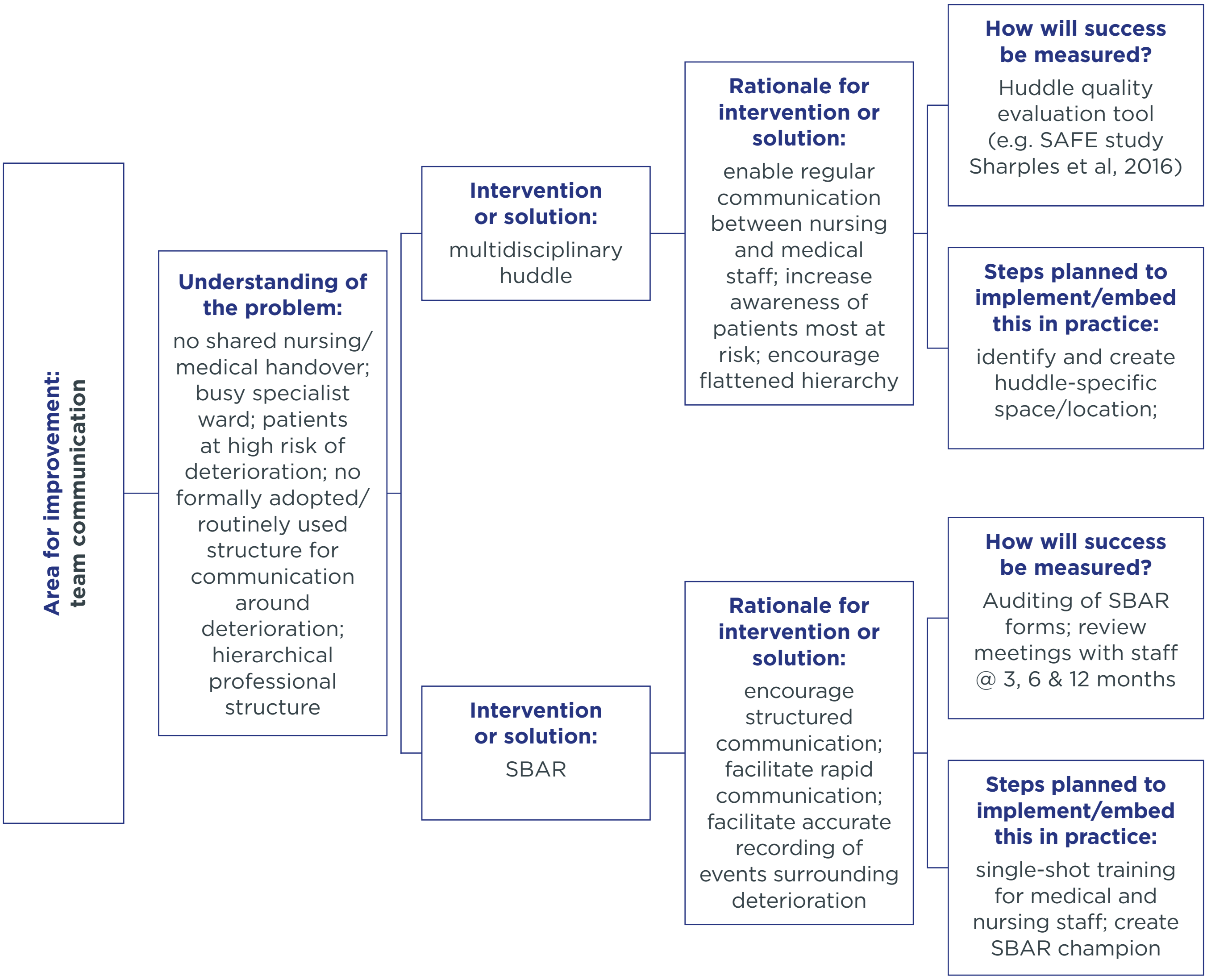

Figure 3 Example of System Assessment Review and Selected Initiatives from Site 2

What were your overall scores for each area of the system (from the system assessment)	What were the key issues identified for the lowest scoring areas?	What were the key initiatives identified to address the lowest scoring areas	What do you think the initiative will achieve?
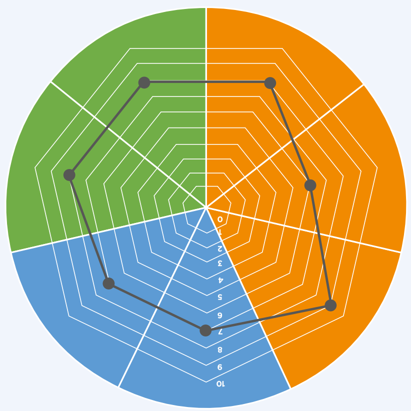	**Families**<br>• Family concern not on PEWS score<br>• Could be more formalised processes for encouraging parent input<br>• Parents may not understand why observations are important<br>**Review relevant information**<br>• Separate doctors and nurses handover<br>• Different handover sheets used for each group<br>• Single occupancy makes it difficult to have overview<br>• Separate ward and assessment unit – communication challenges<br>**Identify and plan for risk**<br>• Staff in assessment unit don't attend ward round or safety huddle<br>• Staffing levels – no supernumerary, has bleep	**1** Introducing modified Shine leaflet and posters<br>**2** Establishing monthly nurse education session<br>**3** Establishing second daily huddle (pm)<br>**4** Joint handover sheet to share information between nursing/medical staff<br>**5** Changing to electronic PEWS	**1** The leaflets and poster will provide staff with a more structured approach to involving family members. The leaflets and posters will provide family members with clear instructions on how to raise concerns, and also act to reinforce the message that their input is welcome and important<br>**2** A monthly education session for all nurses – roughly 3hr slot – would create a more structured approach to training nurses in topics related to identifying potential deterioration on the wards, and providing background information on the purpose and use of the PEWS<br>**3** Increasing opportunities for staff to identify and plan for risk<br>**4** A joint handover sheet – used by both nurses and doctors – would be useful in combining information on patients to be passed on from day shift to night shift. Using SBAR format would ensure key information was captured

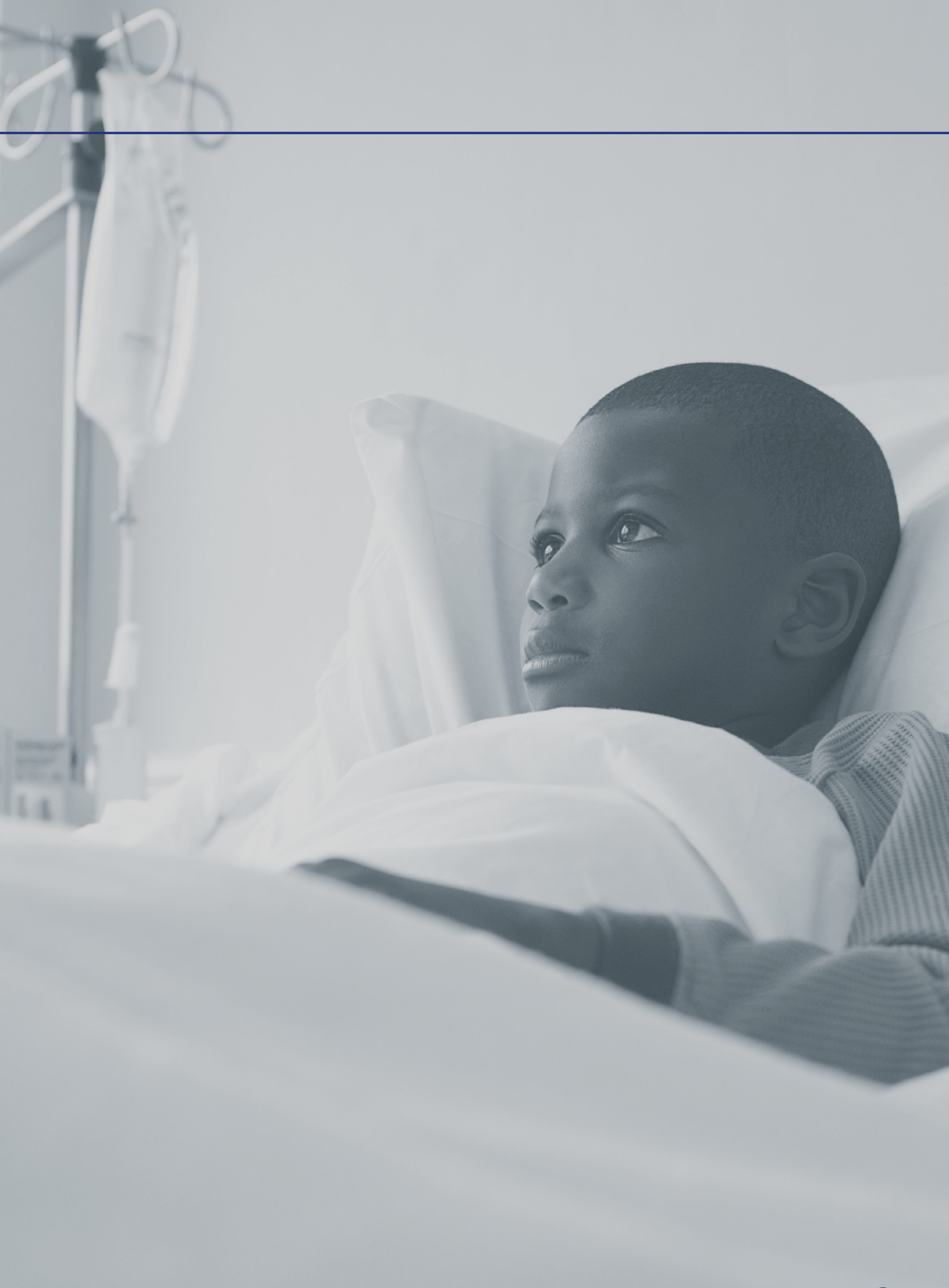

The initiatives chosen to improve each segment of the system varied between teams. For more information about what others have done see Appendix 4.

The core components of a Paediatric Early Warning System: The PUMA Wheel

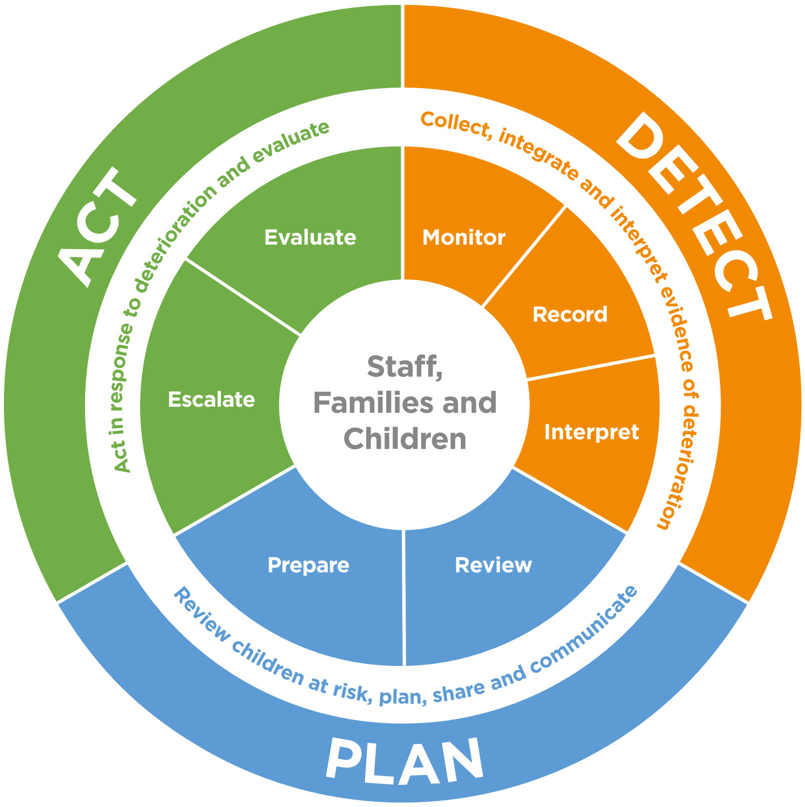

DETECT	PLAN	ACT
• Update observation charts to include normal thresholds [site 4]<br>• Update and disseminate observation policy [site 4]<br>• Introduce modified SHINE leaflet/posters [all sites]<br>• Ward nursing staff to spend time observing HDU staff [site 4]	• Establish a second daily huddle [site 2]<br>• Develop a standard operating procedure for ward rounds [site 1]<br>• Introduce a joint handover sheet for medical/nursing staff [site 2]<br>• Introduce an electronic site board at nursing stations to highlight sickest children [site 3]	• Develop and introduce a formal escalation policy [site 3]<br>• Review and disseminate to all staff the escalation policy [site 4]<br>• Review communication tools for escalation of care [site 4]<br>• Set up monthly critical deterioration review [site1]

Plan your initiative

Before you move ahead with implementing your initiative(s) you need to consider how this change will affect different staff groups, who will lead the initiative(s) and what resources will be required for success.

Identifying potential barriers in advance of implementation and developing strategies to overcome them is an essential part of the implementation process.

To determine whether your improvement initiative(s) have achieved your stated goals, you will need to define your process, outcome and balancing measures before you make any changes:

- **Process measures** monitor quantity and quality (how much did we do, and how well did we do it?)

- **Outcome measures** monitor impact (is anyone better off as a result of the change?)

- **Balancing measures** monitor the impact on other parts of the system (has anything happened elsewhere, as a result of the change?)

See table 2 for examples. Once you have defined your measures, you can assess current practice, before you implement the change.

Table 2 Examples of process, outcome and balancing measures for different initiatives

Initiative	Process	Outcome	Balancing
**Huddles**	• Frequency<br>• Length of huddle	• Incidence of emergency transfer to ICU/HDU<br>• Increased staff perception of safety	• Duration of ward round<br>• Number of patient escalations/red calls
**Introduction of Scoring Chart**	• Incidence of correct completion	• Incidence of emergency transfer to ICU/HDU<br>• Incidence of red calls	• Incidence of escalation with no change in management
**Parent Activation Tool**	• Incidence of parent triggered events	• Incidence of emergency transfer to ICU/HDU<br>• Incidence of red calls	• Incidence of escalation with no change in management<br>• Parental satisfaction

Don't try to measure too much. One or two outcome measures, a few process measures and one balancing measure will usually be enough.

Don't skip this, otherwise you will not know whether your initiative has led to an improvement or had an unintended consequence.

Step 4 Implement and review initiatives

You need to have planned your improvement initiatives before you implement and review them.

Processes for implementing and reviewing your initiative(s) are intertwined; they should take place alongside each other and should be continuous. Implementation is not an easy process, but it can be made easier if:

- Your Improvement Team/organisation agree that there are opportunities for improvement
- Your Improvement Team/organisation agree that the proposed initiative(s) may help
- The work of implementing the initiative(s) is appropriately allocated
- Staff understand their roles and responsibilities in implementing the initiative(s)/changing the way they work
- The improvement initiative(s) does not add to or duplicate workload
- The improvement initiative(s) is supported by your organisation
- Collection of process and outcome measurement data is feasible
- Staff are involved in the collection and/or assessment of process and outcome measurement data
- Process and measurement data is used to monitor and/or modify initiatives

Use worksheet 5 to record how you plan to overcome these issues. Tips and links to supporting resources are also provided.

Reviewing your initiatives

Reviewing your initiatives is an essential aspect of the improvement process. To conduct a proper review of your initiatives you will need to know:

- What you have done
- What has and has not worked
- The reasons that something has or has not worked

Use worksheet 6 to review your initiatives.

Use your process, outcome and balancing measures to help you decide which initiatives need to stop, be amended or continue as planned.

Change is an inevitable part of the implementation process; it is however, difficult to fully control. A new or unexpected policy or organisational change for instance may prevent you from successfully or completely implementing your initiative. You may also find that your initiative does not result in an improvement, or has an unintended consequence. If such a situation occurs, first consider whether it is possible to amend the initiative. If it is not, consider implementing a different initiative to help achieve your goal. You will need to continue to review the impact of any new or amended initiatives.

Learning from practice

Each of the Improvement Teams involved in implementing the PUMA Programme wrote a plan of how they intended to implement each initiative.

Each plan changed as the implementation process progressed. Table 3 shows one iteration of planning and change for some initiatives in one of the hospitals.

Table 3 Implementing and reviewing initiatives; an example

	Initiative 1	Initiative 2	Initiative 3
**Description of initiative**	Introducing modified Shine leaflet and posters	Establishing monthly nurse education session	Establishing second daily huddle
**Who else is contributing to each initiative (name and job title)?**	Senior Nurse children's ward	Advanced Nurse Practitioner x2	SPR - has been doing audit
**What are the tasks (and timescales) associated with each initiative?**	• Review existing Shine tools and amend where necessary (end of May)<br>• Print posters and leaflets (beginning of June)<br>• Distribute posters on wards, and embed process of staff giving family members leaflets on child's admission (end of June)	• Getting approval from ward manager for nursing attendance on days off and claim back (May)<br>• Agreeing the structure of the sessions (May)<br>• Establishing a regular timeslot for the sessions, 3hrs, 9:30-12:30 (May)<br>• Developing the resources required for the training sessions (May)<br>• Deciding on appropriate topics for the sessions, with nurse input (May)<br>• Inviting suitable speakers to deliver training sessions (May)<br>• Booking room<br>• Booking speakers	Initial Implementation has been unsuccessful as audit done showed. Following discussion among the consultants and nurses we have decided to change the format to a "mini huddle". Consultant on from 5pm will after handover, on the assessment ward go to the children's ward and find nurse in charge. They will have an informal discussion about staffing, beds and acutely ill children. Consultant will then feed back to registrar on assessment ward and assessment ward nurses.
**How are you measuring process?**	Audit of number of parents receiving leaflet	Attendance (name and job role)	Audit of how often it happens and who attends

	Initiative 1	Initiative 2	Initiative 3
**How are you measuring outcome?**	Feedback from parents either informally or through more formal questionnaire.	End of session questionnaire (what have they learned?) and Follow-up questionnaire (how has their practice changed?)	
**What have you learnt so far?**	Process has taken a little longer than expected. Need to keep up the momentum.	Things have come together really well and what started out as an idea for monthly sessions has turned into weekly sessions	Second daily huddle not occurring routinely (based on simple audit); poorly attended – held off the ward, so nurses struggled to get there
**Do you plan on making any changes to your initiatives in light of learning?**	Need to address why implementation has been slow.	Initial feedback will guide this	Trying more informal mini-huddle (just consultant x2 and senior nurse), go through brief checklist

Step 5 Sustain progress

Improving your system is a continuous process. Change will continue to occur in policies, organisations, processes, technologies and staff, which will all impact the way in which your system for detecting and responding to deterioration functions.

To maintain your system and continue to progress, it is necessary to embed processes that enable you to continue:

- Assessing your system and identifying opportunities for improvement
- Selecting and implementing initiatives that will function in your setting
- Embedding initiatives in routine practice
- Reviewing progress

Formalise the work of your Improvement Team by setting up regular meetings.

Repeat the System Assessment at set periods (e.g. every two years).

Good luck!

Appendices

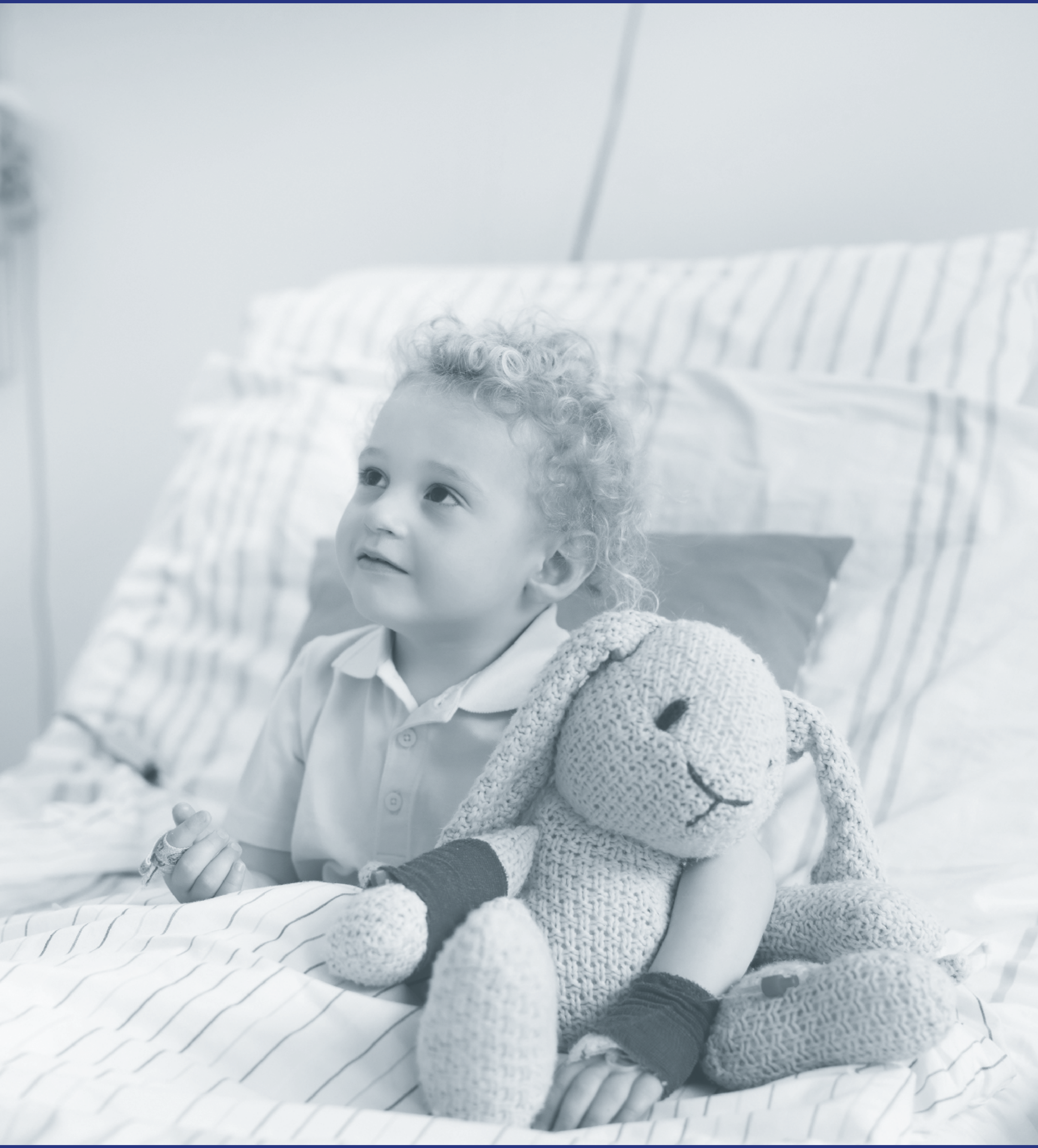

Appendix 1 The core components of a Paediatric Early Warning System: The PUMA Standard

	Proposition	Conceptual requirements
DETECTION	**Detection of deterioration depends on timely and appropriate monitoring of vital signs and relevant risk factors.**	**At a minimum, this requires:**<br>• Staff are aware of which vital signs need to be monitored<br>• Staff are aware of the minimum frequency of observations required for the children in their care<br>• Staff are aware of the need to review the frequency of observations for children in their care<br>• Staff are aware of additional clinical assessments required for children with prior risk factors<br>• Monitoring tasks are allocated to staff members with appropriate skills to conduct them<br>• Staff have access to appropriate equipment to accurately monitor vital signs, and conduct other clinical assessments<br>• Staff are aware of roles and responsibilities for monitoring<br>• Staff have time to conduct accurate timely and appropriate monitoring of vital signs, alongside other work commitments<br>• Staff concern is formally recognised as a valid indicator of deterioration<br>• Staff are supported to develop and use their intuition in detecting signs of deterioration<br>• Staff understand the value of family concerns in the detection of deterioration<br>• Families are involved with defining normal physiological parameters for their child<br>• Families receive guidance about what to do if they are concerned that their child's condition is deteriorating<br>• Staff keep families informed about developments in their child's care and treatment

	Proposition	Conceptual requirements
DETECT	Detection of deterioration depends on timely and appropriate **recording** of signs of deterioration	**At a minimum this requires:**<br>• Staff are aware of the need to record vital signs, family concern and staff concern promptly and accurately<br>• Staff are aware of roles and responsibilities for recording vital signs, family concern and staff concern<br>• Staff have appropriate skills to accurately record vital signs, family concern and staff concern<br>• Staff have access to appropriate equipment to accurately record vital signs, family concern and staff concern<br>• There are an appropriate number of staff to carry out required tasks
	Detection of deterioration depends on timely and appropriate **interpretation** of signs of deterioration	**At a minimum this requires:**<br>• Staff are aware of prior factors that increase children's risk of deterioration (e.g. premature birth)<br>• Staff are aware of roles and responsibilities for interpreting signs of deterioration<br>• Staff take into account, vital signs, family concern and staff concern in assessing the condition of children in their care<br>• Teams have appropriate skills to discern patterns and trends of signs and symptoms<br>• Staff have the opportunity to learn how to interpret signs of deterioration from shadowing more senior staff<br>• Care is organised to enable staff to recognise patterns and trends for children<br>• Families are in a position to discern patterns of signs and symptoms in their child

	Proposition	Conceptual requirements
PLAN	**Planning depends on reviewing indicators of deterioration for each patient.**	**At a minimum this requires:**<br>• For each child, all indicators of deterioration are brought together and kept up to date<br>• There is a regular mechanism for reviewing the status of all children in the ward to identify those children who are a concern<br>• The is a regular mechanism for reviewing staffing levels and skills mix, workload, acuity and admissions
	**Planning depends on staff being aware at ward level of the status of individual patients and the availability of skills and resources, and preparing an appropriate response.**	**At a minimum this requires:**<br>• There is a regular mechanism for communicating the review of all children, staffing levels and other resources to the rest of the team and senior managers<br>• There is a regular mechanism for planning appropriate response to deterioration<br>• Senior staff members are allocated responsibility for managing demand and resources<br>• Senior staff members are allocated responsibility for communicating response plans<br>• There is an action plan for children at risk of deterioration which is shared with families and staff caring for them

	Proposition	Conceptual requirements
ACT	Action depends on clear **escalation** and response processes	**At a minimum this requires:**<br>• A trigger or prompt to act from detection or planning phases<br>• Clearly defined graded escalation and response procedures – agreed at organisational level<br>• Staff receive guidance about how to escalate and respond<br>• Staff understand their roles and responsibilities in the escalation procedure as activators and responders<br>• Staff are encouraged and supported in raising concerns<br>• Families are encouraged and supported in raising concerns<br>• Staff are able to communicate information across professional hierarchies using a structured approach to sharing information<br>• Clear structures to support action, including the use of a 'no false alarms' policy so staff are not deterred from escalating care
	Action depends on **evaluation**	**At a minimum this requires:**<br>• Escalation and response processes are reviewed to promote learning<br>• There is opportunity for staff to discuss differences of opinion in the need for escalation<br>• No blame is assigned to those who escalate

Appendix 2
PUMA: Staff System Assessment Tool

The PUMA Programme

In 2011 a research study compared the child health outcomes and death rate in the UK with other European countries. It was worrying that UK measures of child health were amongst the worst in Europe. Failure to detect and respond to deterioration was one of the factors identified as a contributing factor. The PUMA Programme is an evidence-based approach to improving the detection and response to deterioration in children in UK hospitals.

The aim of the Programme is to improve your system for detecting and responding to deterioration by helping you to:

- Select and implement initiatives that will function in your setting
- Embed initiatives in routine practice
- Review initiatives
- Sustain progress

The PUMA Wheel is a visual summary of PUMA's evidence-based model of the PUMA Standard.

The core components of a Paediatric Early Warning System: The PUMA Wheel

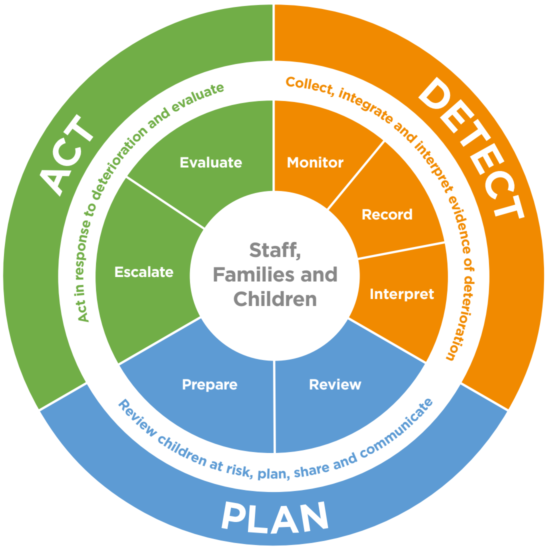

DETECT
Collect, integrate and interpret evidence of deterioration

Monitor	Record	Interpret
Mechanisms to ensure key vital signs (core indicators appropriate for all children and additional indicators for patients specific groups) are monitored reliably with appropriate frequency and accuracy.	Mechanisms to ensure key vital signs and other indicators of deterioration are recorded reliably with appropriate frequency and accuracy.	Mechanisms to ensure key vital signs and other indicators of deterioration are interpreted reliably with appropriate frequency and accuracy.

PLAN
Review children at risk, plan, share and communicate

Review
Mechanisms to ensure indicators of deterioration are reviewed to identify children who are a concern.

Prepare
Mechanisms to ensure staff are aware at ward level of the status of in individual patients and the availability of resources to plan an appropriate response.

ACT
Act in response to deterioration and evaluate

Escalate
Mechanisms to ensure clear escalation and response processes.

Evaluate
Mechanisms to ensure escalation and response processes are evaluated.

Assessing your system

The following questionnaire will help you and your team assess each part of your system:

DETECT	PLAN	ACT
Monitoring, recording and interpreting signs of deterioration	Reviewing children at risk and planning an appropriate response	Escalation and evaluation

Completing this process will help you to evaluate your current system and identify areas for improvement for detecting and responding to deterioration.

Thank you for agreeing to complete the System Assessment Tool.

The aim of the System Assessment Tool is to help us to:

- Review our current system for identifying and responding to deterioration in children on the ward
- Assess what is working well
- Identify parts of the system that could be improved

It is important to answer questions honestly.

No names are collected, and the answers are intended to be anonymous.

About you

What is your job role?

Tick all that apply

- Healthcare assistant ☐
- Staff nurse ☐
- Senior staff nurse ☐
- Sister/ward manager ☐
- Consultant general paediatrician ☐
- Middle grade staff (acting at registrar level) ☐
- Junior doctor (acting below middle grade level) ☐
- Nursing bank/agency/locum staff ☐
- Play specialist ☐
- Relevant allied health professional ☐
- Assistant practitioner ☐
- Consultant intensivist ☐
- Specialty consultants (e.g, respiratory) ☐
- Consultant paediatric surgeon ☐
- Associate specialist/staff paediatrician ☐
- Nursing student ☐
- Medical student ☐
- Other (please specify below) ☐

What type of ward do you work on?

Tick all that apply

- General medical surgical ☐
- Medical ☐
- Surgical ☐
- Specialist (please specify below) ☐

Part 1 DETECT

The following section asks you about your views on how well we currently **monitor**, **record** and **interpret** indicators of deterioration.

Monitor

What mechanisms are in place to ensure that key vital signs, and other indicators of deterioration, are monitored reliably with appropriate frequency and accuracy?

1 **To your knowledge, is there a written policy that explains**

Tick one box per row

	Yes	No	Don't know
Which core vital signs should be monitored for all patients	☐	☐	☐
Which additional signs should be monitored for some patients	☐	☐	☐
How often observations should be carried out with accepted ranges and exceptions	☐	☐	☐
What other clinical assessments should be conducted	☐	☐	☐
Which members of staff are responsible for monitoring observations	☐	☐	☐
That staff concern is an independent and valid indicator of deterioration	☐	☐	☐
That family concerns in an independent and valid indicator of deterioration	☐	☐	☐
How to engage family/parents to establish normal parameters for their child	☐	☐	☐

2 **Have you received formal training (e.g. classroom based) on**

Tick one box per row

	Yes	No	Don't know
Monitoring children's vital signs	☐	☐	☐
Identifying other signs of deterioration not necessarily related to observations	☐	☐	☐
Engaging with family members to help them identify and communicate concerns about their child	☐	☐	☐

Please describe the sort of training you have received in these areas and when/how often

3 Are monitoring tasks allocated to staff members with appropriate skills to conduct them

Tick one box only

Always	☐	Rarely	☐
Usually	☐	Never	☐
Sometimes	☐	Don't know	☐

4 How often is equipment required to monitor vital signs readily available and functioning as it should?

Tick one box only

Always	☐	Rarely	☐
Usually	☐	Never	☐
Sometimes	☐	Don't know	☐

Please list any equipment that is not always available and functioning:

5 Do staff engage with families/parents to establish normal baseline vital signs and behaviours for their child at admission?

Tick all that apply

- Yes – there is a recording of a child's vital signs/behavioural state in the medical notes ☐
- Yes – there is a recording of a child's vital signs/behavioural state in the nursing notes ☐
- No ☐
- Don't know ☐

6 What opportunities other than scheduled ward rounds are there for staff to feedback management plans to families?

Tick all that apply

- There is a specific management session for families at a set time of day ☐
- Its part of the observation process (i.e. plan discussed immediately after vital signs observations) ☐
- There is a link nurse/doctor for each parent to ask questions ☐

Record

What mechanisms are in place to ensure key vital signs and other indicators of deterioration are recorded reliably with appropriate frequency and accuracy?

7 To your knowledge, is there a written policy that explains

Tick one box per row

	Yes	No	Don't know
Which members and grades of staff are responsible for recording observations	☐	☐	☐
Minimum staffing levels	☐	☐	☐

8 Have you received formal training (e.g. classroom based) on recording children's vital signs?

Tick one box only

Yes ☐

No ☐

Don't know ☐

Please describe the sort of training you have received in these areas and when/how often

9 To what extent are scheduled observations immediately documented in the patient's observation chart?

Tick one box only

Always	☐	Rarely	☐
Usually	☐	Never	☐
Sometimes	☐	Don't know	☐

10 How often is equipment required to record vital signs readily available and functioning as it should?

Tick one box only

Always	☐	Rarely	☐
Usually	☐	Never	☐
Sometimes	☐	Don't know	☐

11 If a member of staff is concerned about a child is this information formally recorded?

Tick one box only

Always	☐	Rarely	☐
Usually	☐	Never	☐
Sometimes	☐	Don't know	☐

Interpret

What mechanisms are in place to ensure key vital signs and other indicators of deterioration are interpreted reliably with appropriate frequency and accuracy?

12 Is there a written policy that highlights the responsibility of each grade to escalate and action?

Tick one box only

Yes	☐
No	☐
Don't know	☐

13 Have you received formal training (e.g. classroom based) on

Tick one box per row

	Yes	No	Don't know
Prior factors that increase children's risk of deterioration	☐	☐	☐
Interpreting indicators of deterioration, including vital signs, family concern and staff concern	☐	☐	☐
Managing variation in observations over time (as opposed to escalation for one set of observations)	☐	☐	☐

Please describe the sort of training you have received in these areas and when/how often

14 For a given shift, how often are nurses assigned to a patient they have previously looked after?

Tick one box only

- Always ☐
- Usually ☐
- Sometimes ☐
- Rarely ☐
- Never ☐
- Don't know ☐

15 Are features of deterioration highlighted and explained to families?

Tick all that apply

- They receive written information ☐
- They receive verbal information ☐
- They receive an initial briefing on arrival to ward ☐
- They do not receive any formal information ☐
- Don't know ☐

16 Are junior staff given the opportunity to learn how to interpret signs of deterioration by shadowing more senior staff?

Tick one box only

- Yes ☐
- No ☐
- Don't know ☐

Part 2 PLAN

The following section asks you about your views on how well we currently **review** indicators of deterioration at ward level and the availability of resources **to plan** an appropriate response.

Review

What mechanisms are in place to ensure indicators of deterioration are reviewed to identify children who are a concern?

1 Do your observation charts bring together vital signs and other assessments into an overall score (e.g. an Early Warning Score)

Tick one box only

- Yes ☐
- No ☐
- Don't know ☐

2 Do you attend a handover at the beginning/end of each shift that highlights patients most at risk of deterioration?

Tick one box only

- Yes ☐
- No ☐
- Don't know ☐

2a If yes, who typically takes part in handovers?

Tick one box only

- Just medical staff ☐
- Just nursing staff ☐
- Medical and nursing together ☐
- Medical and separately ☐
- Don't know ☐

2b If yes, what information is included in these handovers?

Tick all that apply

- Vital signs/observations ☐
- Other clinical assessments ☐
- PEW score (if a score exists) ☐
- Staff concern ☐
- Family concern ☐

2c If yes, what information is included in these handovers?

Tick one box only

Yes ☐
No ☐
Don't know ☐

If yes, please name the tool you use (if known)

3 Is there typically a nurse in charge for each shift?

Tick one box only

Yes ☐
No ☐
Don't know ☐

3a If yes, does this nurse in charge typically have a patient case load?

Tick one box only

Yes – a full patient load ☐
Yes – a partial patient load ☐
No ☐
Don't know ☐

3b If yes, to what extent is the nurse in charge typically aware of the children at most risk of deterioration on the ward?

Tick one box only

Always aware of the children most at risk of deterioration ☐
Reliant on handover to be aware of the children most at risk of deterioration ☐
Rarely aware of the children at most risk of deterioration ☐

Prepare

What mechanisms are there to ensure staff are aware at ward level of the status of in individual patients and the availability of resources to plan an appropriate response?

4 To you knowledge, is there a written policy that describes

Tick one box per row

	Yes	No	Don't know
Which members and grades of staff are responsible for communicating response plans	☐	☐	☐
Which members and grades of staff are responsible for assessing and managing demand and resources at ward level	☐	☐	☐

5 Do staff who act in 'in charge' roles on the ward received training on identifying and planning for risk?

Tick one box only

Yes ☐
No ☐
Don't know ☐

6 To what extent is the doctor/nurse in charge typically aware of staffing levels on the ward?

Tick one box only

Always	☐	Rarely	☐
Usually	☐	Never	☐
Sometimes	☐	Don't know	☐

7 How often are the doctor/nurse in charge able to effectively communicate plans about how they are going to manage and demand resources on the ward?

Tick one box only

Always	☐	Rarely	☐
Usually	☐	Never	☐
Sometimes	☐	Don't know	☐

8 Action plans are routinely developed for children at risk of deterioration and shared with families and staff caring for them.

Tick one box only

Always	☐	Rarely	☐
Usually	☐	Never	☐
Sometimes	☐	Don't know	☐

Part 3 ACT

The following section asks you about your views on how well **escalate** and **evaluate** escalation and response procedures.

Escalate

What mechanisms are in place to ensure clear escalation and response processes?

1 To your knowledge, is there a written policy that explains:

Tick one box per row

	Yes	No	Don't know
Graded escalation and response processes	☐	☐	☐
Which members of staff are responsible for escalating and responding	☐	☐	☐
How staff should raise concerns about patient deterioration not necessarily related to observations	☐	☐	☐

2 Have you received formal training (e.g. classroom based) on

Tick one box per row

	Yes	No	Don't know
When and how to escalate care for a child who you suspect to be deteriorating	☐	☐	☐
Communicating critical information about a deteriorating child	☐	☐	☐

Please describe the sort of training you have received in these areas and when/how often

3 Do your observation charts provide guidance on what might be considered 'normal' ranges for different vital signs/observations?

Tick one box only

Yes ☐

No ☐

Don't know ☐

4 How are families/parents typically informed about how to escalate concerns about their child?

Tick all that apply

- They receive written information ☐
- They receive verbal information ☐
- They receive an initial briefing on arrival to ward ☐
- They do not receive any formal information ☐
- Don't know ☐

5 How are families/parents typically informed about how to voice their concerns if they feel they have not been listened to by their designated team?

Tick all that apply

- They receive written information ☐
- They receive verbal information ☐
- They receive an initial briefing on arrival to ward ☐
- They do not receive any formal information ☐
- Don't know ☐

6 How often do staff on the ward use a structured method or tool (e.g. SBAR) to communicate information about a deteriorating child?

Tick one box only

- Always ☐
- Usually ☐
- Sometimes ☐
- Rarely ☐
- Never ☐
- Don't know ☐

7 In your experience, to what extent are staff on the ward able to communicate information across professional, disciplinary and hierarchical boundaries?

Tick one box only

- Always ☐
- Usually ☐
- Sometimes ☐
- Rarely ☐
- Never ☐
- Don't know ☐

8 To what extent would you agree with the following statement: "If a patient's observations/vital signs appeared normal but I felt they were becoming unwell, I would feel confident about raising concerns with more senior colleagues"

Tick one box only

Strongly agree	☐	Disagree	☐
Agree	☐	Strongly disagree	☐
Neither agree or disagree	☐		

9 In your experience, to what extent are staff concern about a child positively received by senior colleagues, regardless of the outcome?

Tick one box only

Always	☐	Rarely	☐
Usually	☐	Never	☐
Sometimes	☐	Don't know	☐

Evaluate

What mechanisms are in place to ensure escalation and response processes are evaluated?

10 How often are your escalation and response processes reviewed to promote learning?

Tick one box only

6 monthly	☐	Every three years	☐
Annually	☐	Other	☐
Every two years	☐		

11 There is a forum for staff to discuss escalation processes, share good practice and learn from different approaches?

Tick one box only

Yes	☐
No	☐
Unsure	☐

12 There is a written 'no blame' safety culture

Tick one box only

Yes ☐

No ☐

Unsure ☐

Appendix 3 Family Feedback Form

We are looking at our ward and thinking about what could be done better. Your views as family members are important to us. Please take a moment to answer the following questions. Your answers are anonymous and will not have any impact on the care that you and your child receive.

1 Please tell us about your experience of getting to know the ward: did you receive any kind of introduction/orientation when you arrived? If so, what was it like? What other information would you like to receive about the ward, and how?

2 When you arrived on the ward, did staff ask you about what is normal for your child's health (e.g. their usual colour, energy levels, feeding, breathing, behaviour)?

- Yes ☐
- No ☐
- Don't remember/Don't know ☐

3a Have you been told about what to do if you think that your child's health is getting worse?

- Yes ☐
- No ☐
- Don't remember/Don't know ☐

If no, please go to question 4. If don't remember/don't know, please go to question 4.

3b How were you told about this?

Please tick all that apply

- I was given a leaflet or booklet ☐
- I was told when I arrived on the ward ☐
- I saw a poster ☐
- I asked a nurse or doctor ☐

4a Have you been told about who to talk to if you feel you have not been listened to by the team caring for your child?

- Yes ☐
- No ☐
- Don't remember/Don't know ☐

If no, please go to question 5. If don't remember/don't know, please go to question 5.

4b How were you told about this?

Please tick all that apply

- I was given a leaflet or booklet ☐
- I was told when I arrived on the ward ☐
- I saw a poster ☐
- I asked a nurse or doctor ☐

4c How able would you feel to talk to or approach the team caring for your child about this? (please circle one of the numbers below)

**Not able at all**	0	1	2	3	4	5	6	7	8	9	**Very able**

5 Please tell us about any other ways you have been encouraged to recognise when your child's health is getting worse and/or share your concerns with staff

6 Finally, thinking about how we tell you about what to do when you think your child's health is getting worse – what do you think we do well? What do you think we could do better?

Things we are doing well:	Things we could do better:

Thank you for taking the time to complete this for us! Please put your completed questionnaire in the "family questionnaire" box located at reception.

Appendix 4 What others have done

A range of different initiatives have been used to improve the different parts of the system: Detect, Plan and Act.

Information about the initiatives and their aim came from two different sources:

- Three systematic reviews conducted during the early stages of PUMA

- The experience of clinical teams who have implemented the PUMA Programme in practice

Figure 4 shows all the initiatives proposed in the literature and all those proposed by some of the teams who have been through the PUMA improvement process.

> Remember it is very likely that there are other initiatives that may be better suited to your setting.

Figure 4 Initiatives used to improve detection, planning and action in paediatric early warning systems

	Proposition	Improvement initiatives proposed in the literature	Improvement initiatives proposed by teams who have implemented PUMA in practice
DETECTION	Detection of deterioration depends on timely and appropriate **monitoring, recording** and **interpretation** of signs of deterioration	**Skills training: accurate, timely and appropriate monitoring**<br>• Goal: Staff are aware of which core and additional indicators need to be monitored<br>• Goal: Staff have the skills to undertake observations<br>• Goal: Staff are aware of the need to review the frequency of observations for children in their care<br>**Focused education: recognising signs of deterioration**<br>• Goal: Staff have appropriate skills to regularly monitor and accurately record key physiological parameters<br>**Induction:**<br>• Goal: Staff are aware of monitoring processes in a specific ward<br>**Observations policy**<br>• Goal: Staff are aware of the frequency of observations required for the children in their care<br>• Goal: Staff are aware of roles and responsibilities<br>**Regular equipment checks**<br>• Goal: Staff have access to appropriate equipment to monitor and accurately record vital signs<br>**Track and Trigger Tool**<br>• Goal: Observations of key physical parameters are structured, accumulated and displayed so that patterns and trends can be readily detected and staff are prompted to make observations at the required frequency	**Formally establish the Deteriorating Child Study Day across the health board (Site 4)**<br>• Goal: Ensure staff have the skills for for monitoring etc. to identify and detect the deteriorating child<br>**Posters & cards to nursing/medical staff with abnormal thresholds for vital signs (Site 4)**<br>• Goal: Improve staff awareness of normal ranges<br>**Update observation charts to include age-related abnormal thresholds (Site 4)**<br>• Goal: Improve staff awareness of normal ranges<br>**Update and disseminate existing observation policy (Site 4)**<br>• Goal: Improve awareness of policy, particularly around frequency and type of observations<br>**Equipment inventory (Site 4)**<br>• Goal: Ensure necessary equipment is available and functioning

	Proposition	Improvement initiatives proposed in the literature	Improvement initiatives proposed by teams who have implemented PUMA in practice
**DETECT**		**Routine opportunities for more experienced staff to coach those less experienced (clinical supervision)**<br>• Goal: Less experienced staff learn pattern recognition from more experienced staff<br>**Skills training: working as a team**<br>• Goal: Staff respect and value each other's opinion<br>**Track and Trigger Tool: staff concern a core parameter**<br>• Goal: Staff recognise concern as a valid indicator of deterioration<br>• Goal: Staff concern included as a key parameter in the detection of deterioration<br>**Parent information leaflets and posters**<br>• Goal: Parents are informed that their views matter, and provided guidance about how to share their concerns with staff<br>**Skills training: family engagement**<br>• Goal: Staff respect and value family engagement<br>• Goal: Staff recognise family concern as a valid indicator of deterioration<br>• Goal: staff have skills to involve parents in child's care<br>**"Planning care together" forms**<br>• Goal: Parents and staff can share, discuss and document parental concerns.<br>• Goal: structured mechanism of involvement<br>**"What to expect" script**<br>• Goal: Parents receive guidance about how to articulate their concerns<br>**Track and Trigger Tool: parent concern a core parameter**<br>• Goal: Family concern included as a key parameter in the detection of deterioration	**Ward nursing staff to spend time observing HDU staff (Site 4)**<br>• Goal: Enhance skills of nursing staff<br>**Introduce parent information and leaflet (modified from SHINE) and posters (Sites 1, 2, 3, 4)**<br>• Goal: Improve parent empowerment and involvement

	Proposition	Improvement initiatives proposed in the literature	Improvement initiatives proposed by teams who have implemented PUMA in practice
PLAN	Planning depends on **reviewing** indicators of deterioration for each patient staff being aware at ward level of the status of individual patients and the availability of skills and resources, and **preparing** an appropriate response.	**Skills training: working as a team**<br>• Goal: Staff develop the skills and behaviours required for effective communication, coordination and collaboration and understand their purpose and importance<br>**"Watcher"**<br>• Goal: formalise process where the bedside nurse, and clinician, proactively identify risk, which includes assessment of information from children, families and staff<br>**Common information space**<br>• Goal: Information on deterioration from children, staff and families are brought together, displayed together, regularly updated and easily accessible<br>**Structured and standardised handover**<br>• Goal: Key information from children, staff and families is systematically shared in a structured method, during handovers, between shifts or between staff in the same or different clinical areas<br>**Huddles**<br>• Goal: Senior staff allocated responsibility for knowing which patients are at high risk of deterioration meet with those responsible for conducting observations and liaise with families to discuss plans for individual patient management	**Introduce joint handover sheet to share information between nursing/medical staff (Site 2)**<br>• Goal: To combine information on patients to be passed on from day to night shift, using SBAR format<br>**Use identifiable markers in huddles, board rounds and handover sheets for at risk children (Site 4)**<br>• Goal: Increase shared awareness of at risk children in order to direct resources appropriately<br>**Introduce electronic site board at nursing stations to highlight sickest children (Site 3)**<br>• Goal: Improve the multi-disciplinary communication on the medical wards so that children at risk of deterioration are identified on one system that all nurses/AHP and medics have access to<br>**Review and adjust existing communication mediums (handovers, safety briefings, huddles, ward rounds, etc.) (Site 3)**<br>• Goal: Improve the multi-disciplinary communication on the medical wards so that children at risk of deterioration are identified<br>**Review handover content and possibility of including nursing staff in medical handover (Site 4)**<br>• Goal: Improve communication between staff<br>**Re-establishing a watch-stander/nurse in charge role (Site 4)**<br>• Goal: Improve ability to identify at-risk children and increase overall situational awareness

	Proposition	Interventions	
PLAN	**Planning depends on staff being aware at ward level of the status of individual patients and the availability of skills and resources, and being able to plan appropriate response**	**Skills training: working as a team**<br>• Goal: Staff develop the skills and behaviours required for effective communication, coordination and collaboration and understand their purpose and importance<br>**Common information space**<br>• Goal: Information on deterioration (physiological, staff concern, family concern) are brought together, displayed together, regularly updated and easily accessible<br>**Structured and standardised handover**<br>• Goal: Key information about patients, staffing levels and other resources is systematically recorded and shared<br>**Huddles**<br>• Goal: Senior staff allocated responsibility for knowing which patients are at high risk of deterioration meet with those who oversee patient flow and staffing to discuss plans for overall patient management<br>**Watch-stander/nurse in charge**<br>• Goal: Senior staff member, without a patient load, responsible for having overview of all children, staff levels and skills	**Establish second daily huddle (pm) (Site 2)**<br>• Goal: Increase frequency of communication about risk between staff<br>**Moving to 3x daily huddles / board rounds (Site 4)**<br>• Goal: Increase opportunities for communication<br>**Establish a training course for staff on situational awareness (Site 4)**<br>• Improve staff situational awareness skills<br>**Develop standard operating procedures (SOP) for ward rounds (Site 1)**<br>• Goal: Improve variable quality of ward rounds by increasing nurse and parent involvement

	Proposition	Interventions	
ACT	Action depends on clear **escalation** and response processes	**Activation policy**<br>• Goal: All staff and parents are aware of escalation and response criteria<br>**Activation criteria displayed throughout hospital**<br>• Goal: All staff and parents are aware of escalation and response procedures and roles and responsibilities<br>**Staff induction**<br>• Goal: All staff are aware of escalation and response procedures and roles and responsibilities<br>**Track and Trigger Tool**<br>• Goal: Alerts staff to signs of deterioration and prompts the appropriate response	**Introduce formal, written escalation policy (Site 3)**<br>• Goal: To ensure a more consistent approach to empower staff to call for help, and to make clear the roles and expectations of senior staff in responding to escalation requests.<br>**Review and disseminate escalation policy (Site 4)**<br>• Goal: Improve staff awareness of policy and roles and responsibilities in escalation<br>**Roll-out own in-house e-learning package for nursing/medical staff (focused on spotting the sick child) (Site 4)**<br>• Goal: Ensure staff receive training on communicating critical information<br>**Review communication tools to aid escalation of care (Site 4)**<br>• Goal: Improve staff skills in communicating critical information
	Action depends on **evaluation** of escalation and response processes	**'No false alarms' policy**<br>Goal: clear structures to support action and a supportive culture that does not penalise individual decision-making<br>**Escalation policy linked with administrative arm**<br>Goal: Reinforce the system and measure outcomes	

Worksheets

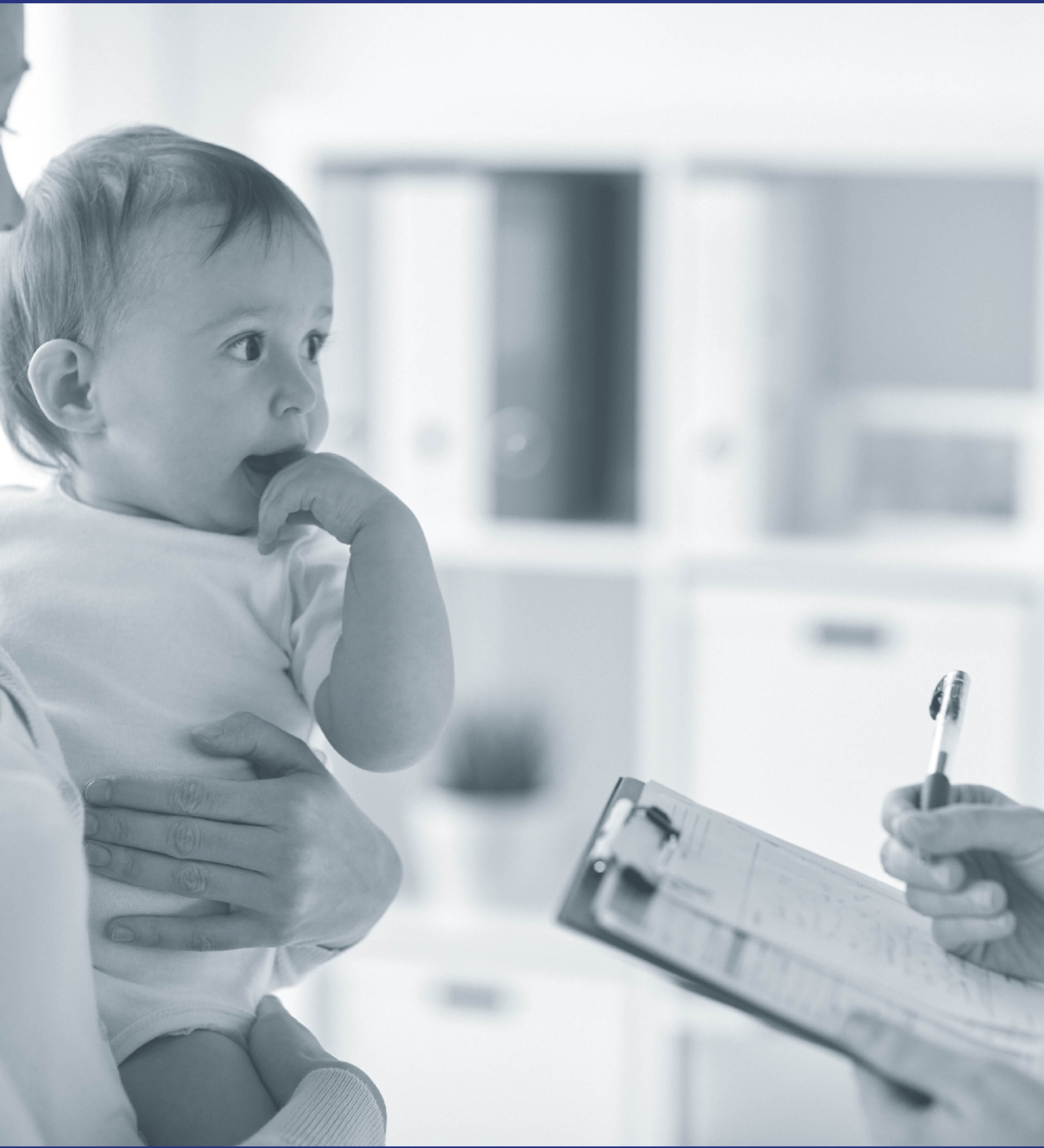

Worksheet 1 Your essential Improvement Team members

Improvement Team Member (add others if required)	Name (staff position)
Organisational sponsor	
Clinical and technical expert – Doctor	
Clinical and technical expert – Nurse	
Implementation Lead	

Worksheet 2 System assessment checklist

There are a number of tasks that are essential for ensuring the System Assessment is completed. Use the following table to record who is responsible for each task and when it needs to be completed.

Task	Person responsible	Deadline/agreed date
Agree a date for Improvement Team to meet to review completed tools and complete summary assessment		
Agree date for administering Family Feedback Tool		
Designated staff member ensures a minimum of 10 families complete Family Feedback Tool		
Designated staff member ensures representatives from each of the mandatory staff groups complete System Assessment Tool		
Designated staff member collects and stores completed System Assessment Tools		
Designated staff member collects and stores completed Family Feedback Tools		
Local Improvement Team meet to review completed tools and complete summary assessment		
Organisation Improvement Team meet to review completed tools and complete summary assessment		

Worksheet 3 Summary assessment and radar

DETECT

Area	Strengths	Weaknesses
Describe strengths and weaknesses for ensuring timely and appropriate **monitoring** of vital signs and relevant risk factors		
Describe strengths and weaknesses for timely and appropriate **recording** of signs of deterioration		
Describe strengths and weaknesses for timely and appropriate **interpretation** of signs of deterioration		

	Area	Strengths	Weaknesses
PLAN	Describe strengths and weaknesses for **reviewing** indicators of deterioration for each patient.		
	Describe strengths and weaknesses for ensuring staff are aware, at ward level, of the status of individual patients and the availability of skills and resources, and are able to prepare appropriate response		
ACT		Strengths	Weaknesses
	Describe strengths and weaknesses for clear escalation and response processes		
	Describe strengths and weaknesses for evaluating escalation and response processes		

Use the information in your strengths and weakness table above to rate each of the seven elements of the PUMA Wheel on a 0-10 scale:

- A high score here would represent an area in which the individual assessments point to your ward having a number of robust, standardised processes
- A low score would represent an area in which individual assessments point to your ward having few or no standardised processes.

The radar below will give you a visual summary of how well the ward is currently performing in the different areas of the PUMA Wheel.

Figure 5 Example radar

Evaluate
To what extent do you have standardised processes in place to evaluate escalation and response processes

Escalate
To what extent are there standardised processes in place to ensure timely escalation and response

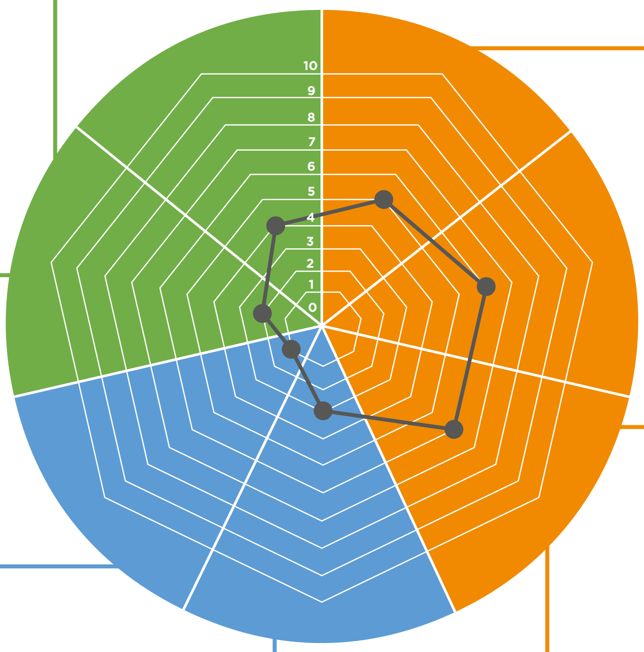

Prepare
To what extent do you have standardised processes in place for ensuring staff are aware, at ward level, of the status of individual patients and the availability of skills and resources, and are able to prepare appropriate responses

Review
To what extent do you have standardised processes in place for reviewing indicators of deterioration for each patient

Monitor
To what extent do you have standardised processes in place to ensure key vital signs (core indicators appropriate for all children and additional indicators for specific patient groups) and relevant risk factors are monitored reliably with appropriate frequency and accuracy?

Record
To what extent do you have standardised processes in place that support and enable families to ensure timely and appropriate recording of signs of deterioration

Interpret
To what extent do you have standardised processes in place that ensure timely and appropriate interpretation of signs of deterioration

Worksheet 4 Action planning

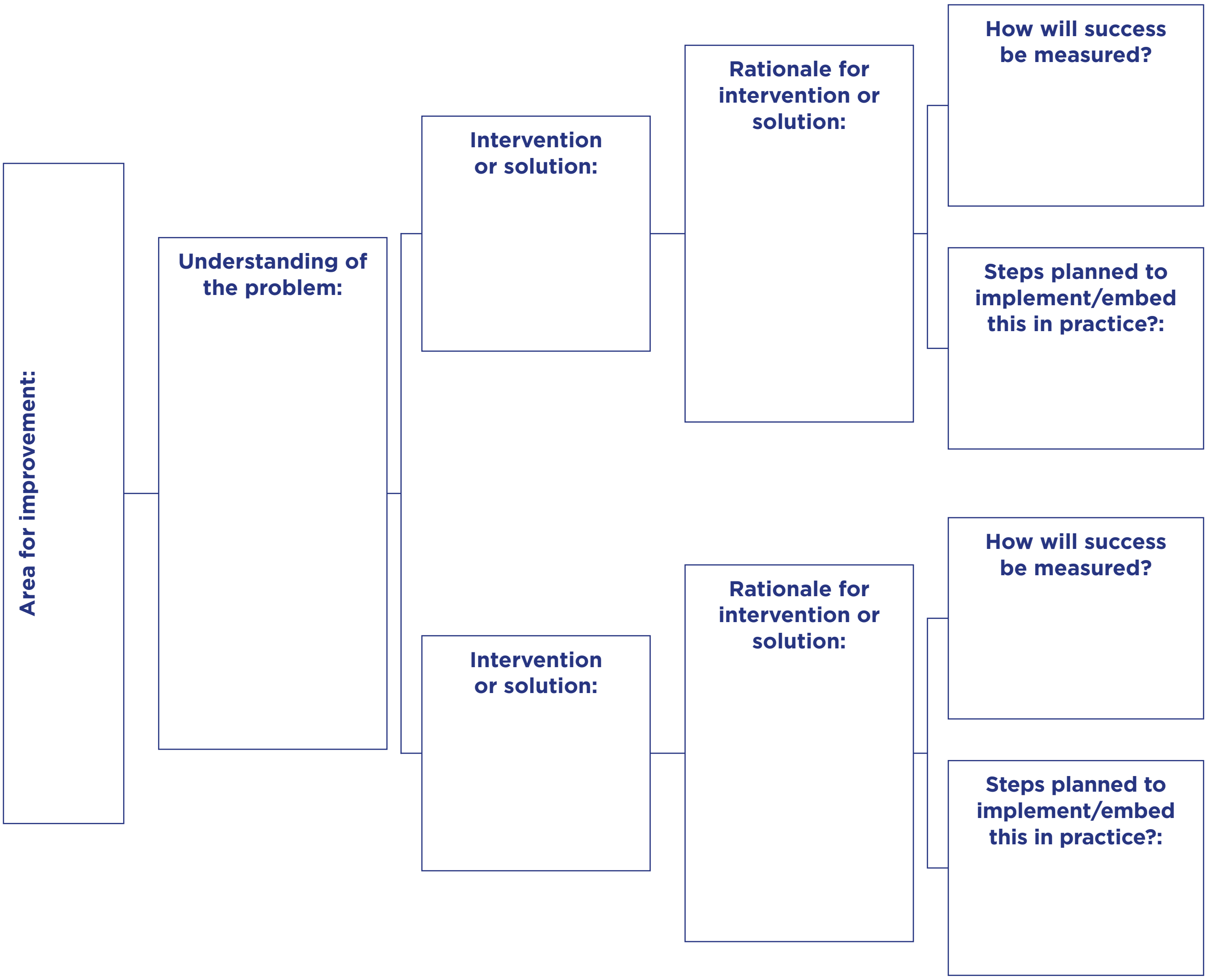

Worksheet 5 Implementing your initiatives

Question	Notes	Tips/supporting resources
**Does your team agree that there are areas for improvement?**		• PUMA slide set: the PUMA Programme: an introduction<br>• Feedback findings of the System Assessment (Radar)
**Does your team/ organisation agree that the proposed initiatives may help?**		• Appendix 4: what others have done<br>• Engage staff at all levels in the *System Assessment*<br>• Share *Action Plan* with staff
**Is the work of the initiative appropriately allocated to staff?**		• Discuss *Action Plan* with staff
**Do staff understand their roles and responsibilities in implementing the initiative?**		• Discuss *Action Plan* with staff
**Does it add work or duplicate effort?**		• Monitor implementation process using Plan Do Study Act (PDSA) cycles<br>• http://www.ihi.org/resources/Pages/HowtoImprove/ScienceofImprovementTestingChanges.aspx
**Are the improvement initiatives adequately supported by your host organisation?**		• Share *System Assessment* results (Radar) with organisational sponsor
**Are staff involved in the collection and/or assessment of process and outcome measurement data?**		• **Measurement Plan Framework** http://www.qihub.scot.nhs.uk/media/340181/2012-06-15_measurement_improvement_journey_process.pdf<br>• **Measurement Plan Summary Form** http://www.qihub.scot.nhs.uk/knowledge-centre/quality-improvement-tools/measurement-plan-summary-form.aspx<br>• **Data Collection Plan Form** http://www.qihub.scot.nhs.uk/knowledge-centre/quality-improvement-tools/data-collection-plan-form.aspx
**Are the process and measurement data used to monitor and/or modify initiatives?**		• See links to measurement documents above

Adapted from the Normalisation Process Theory Toolkit, http://www.normalizationprocess.org/npt-toolkit/

Worksheet 6 Reviewing your initiatives

	Initiative 1	Initiative 2	Initiative 3
**Description of initiative**			
**Who else is contributing to each initiative (name and job title)**			
**What are the tasks (and timescales) associated with each initiative?**			
**How are you measuring process?**			
**How are you measuring outcome?**			
**What have you learnt so far?**			
**Do you plan on making any changes to your initiatives in light of learning?**			

For more information please contact:

Dr. Amy Lloyd
Research Fellow at the Centre for Trials Research,
Cardiff University

Email: lloyda1@cardiff.ac.uk
