# Supplementary material for: Development, implementation and evaluation of an evidence-based paediatric early warning system improvement programme: the PUMA mixed methods study
Source: BMC Health Serv Res. 2022 Jan 2;22:9. doi: 10.1186/s12913-021-07314-2 (PMC8722056; doi:10.1186/s12913-021-07314-2)
Supplement: Supplementary file 5 — Additional file 5. Summary of paediatric early warning system improvement initiatives across all case studies. [file 12913_2021_7314_MOESM5_ESM.docx]

Additional Material 5: Summary of paediatric early warning system improvement initiatives across all case studies

| **Site** | **Proposed Initiative** | **Element of system being addressed** | | **Understanding/source of the problem** | **Implemented**  **Y/N/Initiative changed** | | **Embedded**  **Y/N** | | - **Promoting/hindering factors** |
| --- | --- | --- | --- | --- | --- | --- | --- | --- | --- |
| 1 | Develop a tool to encourage family engagement | Detect | | - Fairly poor at empowering parents – no formal process - Cubicles – isolated from finding staff, visibility of staff - No formal involvement of parents in ward rounds | Y | | Y | | - Low-tech approach adopted on HDU: ‘mypad’ marker board used by staff and families to record key information and ask/respond to questions. Other wards waiting for Trust level development of a parental information platform. |
| 1 | Retraining on PEWS recognition and response to deterioration including NICE sepsis screening for front-line clinical staff | Detect, Plan, Act | | - Need to improve recognition and response to deteriorating patients… Evidence that signs of deterioration including sepsis have not always been managed as quickly as desired | Y | | Y | | - TRUST-mandated change: significant institutional support/pressure to implement   *“there have been a lot of other sort of things going on within the Trust that have taken quite a bit of priority. A lot of work has gone on around sepsis, and particularly getting that embedded into the whole Trust really, not just our unit. So we've done a lot of work around sepsis, to do with competencies, and training.”* |
| 1 | Implement SOP for out of hours working for on-call medical teams – prioritising sickest children (hospital-wide) | Detect, Plan, Act | | - Need to improve how on-call junior medical team prioritise workload to identify and respond to the sickest patients across the hospital... the weekend ward round is often still ongoing at night time, with all patients being seen and no structured focus on the sickest patients | Y | | Y | | - Medic out of hours/night shift working patterns and routines altered; evening ward round condensed to enable medical staff to prioritise review of sickest children. |
| 1 | Establish a monthly “Critical Deterioration Review Panel” to learn lessons about which aspects of the system need improvement | Detect | - Need to tighten process of identifying and responding to sick children in the hospital… there is occasionally some complacency re. increased PEW score and response is less than adequate… We want to have a review process for all cases of critical deterioration and look for opportunities for prevention which can be fed back to teams in real time. The goal is to learn and continually improve | | Y | N | | - Valued and productive process with institutional support but not enough time required to organise meetings and gather relevant information:   *“there was agreement to do that, but we haven’t got the process running because we haven’t got the, the time”*  *“the preparation of the cases was like probably two or three days work”*   - Difficulty of getting senior/busy staff together to for face to face meetings. - Volume of work unmanageable:   *“on the days that we did it it took Ian and me a whole day to review the cases… in a one month period you could be looking at 25 to 30 cases* | |
| 1 | Implement SOP for ward 1C ward round structure | Plan | - Wide variation in approach to ward round – depending on medical team/lead consultant. - Nursing and medical handovers fragmented, info not available to everyone | | N | N | | - Wide variety of approaches to ward round at present; challenge of discussing and securing agreement across team   Challenge of securing dedicated staff time to drive project forward. | |
| 2 | Introduction of a second daily huddle | Plan | | - Communication between senior nurses and Doctors is more challenging in the afternoon/evening when medical staff are located away from the ward on PAU. | Changed | | Y | | Whilst a formal huddle was not deemed possible, there was increased awareness of the need to improve communication between the two areas. Telephone calls between the ward and PAU now occur more frequently. In addition, the two areas have been brought together through a rotation of band 6s working on the PAU. A safety huddle that takes place at 9am on the main ward seems to have taken on the momentum for addressing what the second daily huddle initially set out to do. |
| 2 | Joint handover sheets, using SBAR | Plan | | - Currently, nursing and medical handovers are conducted separately (although nurses occasionally attend medical handover). However, there is a feeling that the doctors handover sheets contain information that would be useful for the nurses – and vice versa | Changed | | Y | | •Whilst changes have been made to both handover sheets, a joint one was not progressed.  •“…*that still hasn’t happened the joint handover sheets because it's the logistics of how you get everything on that’s relevant to nursing and the medical teams for all the patients on the ward. We’re getting closer because now, we used to just have the paediatric patients on the medical handover sheets whereas actually the nurses need all the beds on the sheets, so actually the medical sheets, I think it's gradually merging*  •Nurses’ handover sheet has changed to the SBAR. They largely accepted the reasons why it was being implemented and could see the benefits from previous ways of working;  *“You tend to go off your, like a story as in like, oh, and it go, like an SBAR is probably a better way to do it if you can stay focused on like at that mo, you know like it has more of, erm, oh, I don’t know how to describe it. More of a structure” (Interview 4, SN)*  •Importantly, the handover sheet is not a static artefact.  •Senior staff nurse had approval from the ward manager to change the sheet.  *“When I altered the old SBAR and I took it to the ward manager and said these are the suggestions. She said yeah great, just do it, so I changed it” (Interview 9)* |
| 2 | Nurse education | Detect | - At present, there is no structured approach to ongoing nurse education – particularly with regard to PEWS, and identifying potential deterioration on the ward. | | Y | N | | - Staff were being asked to attend in their time-off. | |
| 2 | Introduction of the ‘SHINE’ leaflets and poster | Detect | - Feeling that there is currently no formal process for encouraging family members to input their concerns about possible deterioration | | Y | N | | - Staff consider themselves to carry out tasks already – do not distinguish the tool from previous ways of working;   “I always explain, you know, that um you are your own child’s expert, you know, I don’t know what they’re normally like. So you need to tell me if you think they’re getting worse or whatever, so I always ask, I always include the parents, always do yeah” (Interview 9, SSN).     - Staff consider parents to already raise concerns effectively - Tool considered time consuming and does not fit into routine practice - Lack of awareness of the rationale behind the tool. | |
| 3 | Introduction of electronic site board | Plan | | - No clear mechanisms for highlighting and communicating the most at-risk children between teams. As a result, clinical staff are not always aware of the most at-risk/sick children in their area – so not as efficient as could be at allocating of resources / prioritising high-risk children | Y | | Y | | - Introduced; use dependent on Registrar or SHO handing over. However, has changed the communication between senior nurses and doctors, with them phoning through to doctors handover if they have any concerns about a particular patient |
| 3 | Introduction of new escalation policy | Act | | - No formal escalation policy – and a lack of clarity among clinical staff as to escalation procedures when a child is deteriorating. Currently inconsistent approach. - Critical incident reviews have highlighted difficulties around timely escalation as contributing factor. Escalation during night shifts particularly problematic. | Y | | Y | | - Policy drafted, agreed, finalised and shared. However, awareness on the ward is still low. |
| 3 | Introduction of parent posters (based on SHINE tool, designed to inform parents about how to communicate concerns) | Detect | - Inconsistency in information given to parents/family members when children are admitted. Perception that some family members do not feel empowered to report deterioration of child’s condition when it happens | | Y | N | | - Staff consider themselves to carry out tasks already – do not distinguish the tool from previous ways of working;   “I’ve always told parents, you know, any concerns, let me know, and you know, explained what we’re looking out for because they’re going to be there all the time, so if you’ve got a child with breathing problems and that’s what you’re watching, they usually know. And just make sure that they know and they can come and let you know if there is concerns”.   - Resistance to the idea behind the initiative   I think that's a lot of responsibility for a parent to recognise ... you know it's different saying I think his breathing has changed, can you have a look, I'm a bit worried. Than actually sort of rely on the parents to look at the rolling signs that their child's ... I don't think I agree with that (Interview 8, deputy ward manager) | |
| **Site** | **Proposed Initiative** | **Element of system being addressed** | **Understanding/source of the problem** | | **Implemented**  **Y/N/Initiative changed** | **Embedded**  **Y/N** | | - **Promoting/hindering factors** | |
| 4 | Create posters and cards for staff to signpost abnormal thresholds for vital signs | Detect | | - No normal ranges on current observation charts; need to be clearer, and signpost staff to escalation of care. | Y | | Y | | Staff ownership of initiative and team support: role of task lead delegated to Clinical Educator, working with support of five additional team members.  Cards and posters easily produced – achievable task, manageable without additional resources. Staff carry the cards and refer to them  Credit-card sized tool easily distributed and stored – this has facilitated positive attitude from staff, and sustainable use in practice. Fitted into daily use/routines |
| 4 | Update observation charts to include normal age-related thresholds | Detect | | - Existing obs charts outdated. Need more clarity, for ease of use as a signpost to escalation | Y | | Y | | Required to get institutional support/buy in/sign off/ on changes – lengthy process and outside stakeholders |
| 4 | Update and disseminate observation policy | Detect | | - Lack of awareness of policy. - Some in-house guidelines for frequency for some conditions, but not for all patients - no pro forma. - No definition of ‘routine’ | Y | | N/A | | Emailed and staff requested to sign to say they had received and read |
| 4 | Conduct inventory of equipment | Detect | | - Not enough suitable equipment to enable staff to conduct observations effectively. | Y | | N/A | | Inventory conducted and new equipment ordered |
| 4 | Establish a staff training course on situational awareness | Plan | | - There is no regular training on risk management, staff not routinely trained in situational awareness | Changed | | Y | | - Situational awareness included in statutory training days. |
| 4 | Review and disseminate existing escalation policy | Act | | - Lack of awareness of policy. Some staff unsure of roles and responsibilities around escalation | Y | | Y | | - Updated and shared; staff signed to say they had received it |
| 4 | Explore tools for family/parental involvement | Detect | - Not sure if parents always receive/understand information - Buzzer not often used | | Y | N | | - Ongoing | |
| 4 | Introduce process for identifying ‘watchers’ at each ‘huddle’ and handover. Markers on whiteboard | Plan | - Board rounds and ward rounds could be improved. Increase and maintain staff awareness of children at risk | | Y | N | | Introduced idea of using Identifiable marks on whiteboards and handover sheets to highlight patients at risk – still trying to ensure it is routinely used | |
| 4 | Formally establish Deteriorating Child Study Day across health board | Plan | - Staff not always able to go to training for identifying risk due to staffing issues. Desire to formalise course with Health Board approval, make a bi-annual event. | | N | N | | - Trying to get stamp of approval from RCN | |
| 4 | Roll out in-house e-learning package for nursing and medical staff | Plan | - Staff not always able to go to training for identifying risk due to staffing issues. - Staff feel they need more training on communication of critical information | | N | N | | - Developed but awaiting institutional approval | |
| 4 | Ward nursing staff to spend more time observing HDU staff | Plan | - Inexperienced staff to gain more knowledge, enhance their learning about critically ill children | | N | N | | Not implemented; issues with lack of staffing | |
| 4 | Move to adopt 3x daily ‘ huddles’/board rounds | Plan | - Current board round felt to be very useful for communication and increased situational awareness. - Greater frequency to improve and update patient reviews; plan for AM, 16.30, and 21.00 | | N | N | | Not implemented; not all staff agree it is necessary | |
| 4 | Review handover content. Possibility of including nursing staff in medical handover | Plan | - Handover content could be standardised to aid identification of potential deterioration. Opportunity for information sharing, improved situational awareness, less chance of missing information in separate handovers | | N | N | | Beyond scope of ward, involves other external stakeholders. Possible higher level change to nurse and doctor shift patterns required. | |
| 4 | Re-establish a nursing supernumerary role | Plan | - Compliance with RCN standards. - Widespread agreement on advantages of supernumerary role (advocate for patients and families, greater situational awareness & ward acuity awareness) - Increased ability to identify clinical risk, less patient and family information ‘lost’ from board round | | N | N | | Beyond scope of the ward; involves other external handovers. | |
| 4 | Review communication tools to aid escalation of patient care | Act | - Staff feel they need more training on communicating critical information and junior level communication could be improved | | N | N | | Site lead off sick. | |
